# Comparative analysis of non-coding RNAs in the antibiotic-producing *Streptomyces* bacteria

**DOI:** 10.1186/1471-2164-14-558

**Published:** 2013-08-16

**Authors:** Matthew J Moody, Rachel A Young, Stephanie E Jones, Marie A Elliot

**Affiliations:** 1 Department of Biology and Michael G. DeGroote Institute for Infectious Disease Research, McMaster University, 1280 Main Street West, Hamilton, ON L8S 4K1, Canada

**Keywords:** *Streptomyces*, Non-coding RNA, sRNA, Antisense RNA, Secondary metabolic gene cluster, Antibiotic, RNA degradation

## Abstract

**Background:**

Non-coding RNAs (ncRNAs) are key regulatory elements that control a wide range of cellular processes in all bacteria in which they have been studied. Taking advantage of recent technological innovations, we set out to fully explore the ncRNA potential of the multicellular, antibiotic-producing *Streptomyces* bacteria.

**Results:**

Using a comparative RNA sequencing analysis of three divergent model streptomycetes (*S. coelicolor*, *S. avermitilis* and *S. venezuelae*), we discovered hundreds of novel *cis*-antisense RNAs and intergenic small RNAs (sRNAs). We identified a ubiquitous antisense RNA species that arose from the overlapping transcription of convergently-oriented genes; we termed these RNA species ‘cutoRNAs’, for convergent untranslated overlapping RNAs. Conservation between different classes of ncRNAs varied greatly, with sRNAs being more conserved than antisense RNAs. Many species-specific ncRNAs, including many distinct cutoRNA pairs, were located within antibiotic biosynthetic clusters, including the actinorhodin, undecylprodigiosin, and coelimycin clusters of *S. coelicolor*, the chloramphenicol cluster of *S. venezuelae*, and the avermectin cluster of *S. avermitilis*.

**Conclusions:**

These findings indicate that ncRNAs, including a novel class of antisense RNA, may exert a previously unrecognized level of regulatory control over antibiotic production in these bacteria. Collectively, this work has dramatically expanded the ncRNA repertoire of three *Streptomyces* species and has established a critical foundation from which to investigate ncRNA function in this medically and industrially important bacterial genus.

## Background

Over the last decade, there has been a growing appreciation for the multifaceted roles played by regulatory RNAs in organisms ranging from bacteria to mammals. In bacteria, regulatory non-coding RNAs (ncRNAs) come in many forms, and can impact protein function, transcription initiation, mRNA stability and translation initiation/elongation [1]. Independent ncRNA transcripts can be broadly divided into two categories: *cis-*antisense RNAs (asRNAs) and *trans*-encoded small RNAs (sRNAs) [2]. asRNAs are expressed from the strand opposite their target protein-coding gene, and can negatively or positively impact transcription, translation or mRNA stability [3]. In contrast, most sRNAs, which typically range in size from 40–300 nucleotides, are expressed from intergenic regions. While a small subset of characterized sRNAs affect protein function (*e.g.* 6S RNA [4]), the majority of sRNAs studied to date target one or more mRNAs, influencing transcript stability or translation [1]. A notable difference between asRNAs and sRNAs is that asRNAs share complete complementarity with their mRNA targets, whereas the *trans*-encoded sRNAs have much shorter complementary regions, and different sequences within a sRNA may bind different mRNA targets. ncRNA-mediated regulation has been implicated in a multitude of cellular processes, including stress responses [5], quorum sensing [6] and pathogenicity [7].

The ncRNA potential of bacteria has been explored most thoroughly in *Escherichia coli*[8-11] but in recent years, technological advances in the form of tiling microarrays [12] and RNA sequencing [13-15] have begun to reveal the extent - and the complexity - of ncRNAs in a wide range of bacteria.

The non-coding RNA capacity of *Streptomyces* bacteria is expected to be extensive. The streptomycetes are predominantly soil-dwelling bacteria, and as such must have the means of coping with diverse environmental stresses. They also have a large chromosome (>8 Mb), and a complex life cycle that involves progression through distinct developmental and metabolic stages - processes that are subject to multi-level regulation. During growth on solid culture, the *Streptomyces* life cycle begins with spore germination and hyphal outgrowth. Hyphal tip extension and branching ensue, leading to the formation of an intricate network of vegetative hyphae known as the vegetative mycelium. From these vegetative cells emerge reproductive structures that extend into the air and are termed aerial hyphae. The aerial hyphae then undergo synchronous septation and chromosome segregation, subdividing them into prespore compartments that ultimately develop into chains of dormant exospores [16]. Most streptomycetes grow vegetatively in liquid culture, although several species including *Streptomyces venezuelae*, sporulate under these conditions. Along with their morphological complexity, the streptomycetes are best known for their ability to produce a vast array of secondary metabolites having medical and agricultural importance, including the majority of naturally synthesized antibiotics. Secondary metabolism is co-ordinately regulated with development, initiating during the transition from vegetative to aerial growth (or vegetative to ‘mycelial fragmentation’, for those species that sporulate in liquid culture); in liquid culture, secondary metabolism initiates during entry into stationary phase [16] for the majority of (nonsporulating) streptomycetes.

**Figure 1:**
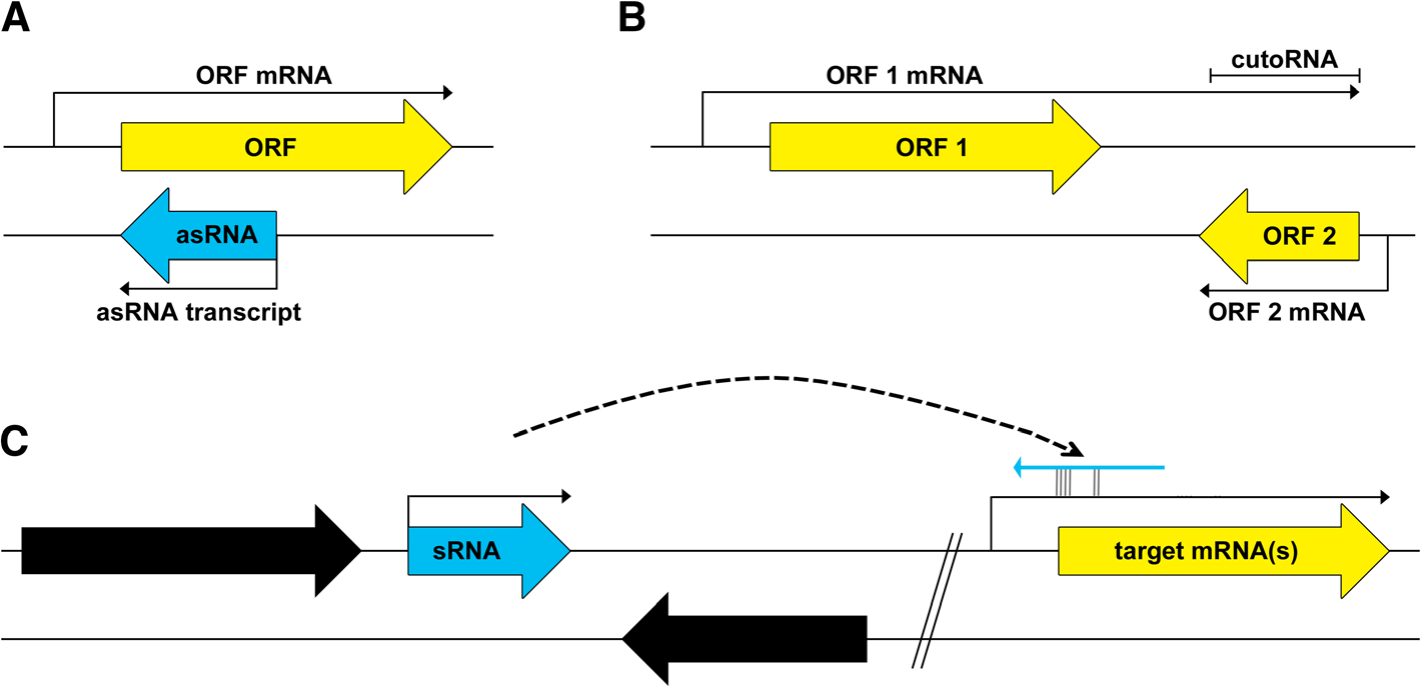
**Schematic illustration of the different classes of non-coding RNAs identified.** Genes are depicted as thick arrows, with protein-coding genes shown in yellow and labeled as ‘ORF’s (open reading frame), non-coding RNAs shown in blue, and black depicting genes of either type. RNA transcripts are shown above their corresponding gene, with transcription initiating at the vertical line, and terminating at the small arrowhead. **(A)** Antisense RNAs (asRNAs) are expressed from a promoter on the strand opposite a protein-coding gene. **(B)** cutoRNAs occur when a long 3′ UTR of an mRNA overlaps with a downstream, convergently transcribed gene. The region of overlap is indicated with a bracketed line. **(C)** Small RNA (sRNA) genes are most commonly found in the intergenic region between genes, and typically target (by imperfect complementary base-pairing) one or more mRNAs expressed from disparate chromosomal locations.

We were interested in exploring the ncRNA potential of *Streptomyces* bacteria throughout the course of their developmental and metabolic cycles. A series of initial investigations had confirmed the existence of ncRNAs in these bacteria [17-20], and this ncRNA repertoire was expanded considerably by an early RNA sequencing study undertaken by Suess and colleagues [21], who identified many asRNAs and sRNAs in the model species *Streptomyces coelicolor*. This pioneering study focused on RNA expression at a single time point during *S. coelicolor* growth in liquid culture. To gain a more comprehensive view of the ncRNA potential of *Streptomyces* bacteria, we undertook a comparative genomics investigation into the transcriptomes of three evolutionarily divergent *Streptomyces* species [22] – *S. coelicolor*, *Streptomyces avermitilis*, and *S. venezuelae* - using RNA harvested at distinct metabolic and developmental stages. *S. coelicolor* and *S. venezuelae* represent classic and emerging model species, respectively, while *S. avermitilis* has been well studied in part due to its production of avermectin, a commercially important insecticidal and anti-parasitic compound. We identified dozens of new conserved sRNAs and asRNAs, including a distinct group of asRNAs termed ‘cutoRNAs’ that resulted from overlap of the 3′ ends of convergently transcribed mRNAs (Figure 1). We also detected an abundance of unique ncRNAs, including many that featured prominently in secondary metabolic biosynthetic clusters.

## Results and discussion

To probe the ncRNA potential of *S. avermitilis, S. coelicolor* and *S. venezuelae*, we performed RNA-Seq using species-specific RNA pools. Each species was grown on the same medium (maltose, yeast extract, malt extract, or MYM), so as to effectively compare their RNA profiles, with the only difference being that *S. avermitilis* and *S. coelicolor* were grown on MYM agar, while *S. venezuelae*, which sporulates in liquid culture, was grown in liquid MYM. For each species, RNA was isolated from cells at major developmental stages (vegetative; aerial hyphae/fragmentation (in the case of *S. venezuelae*); spores). The RNA samples for each species were then pooled and used to generate two libraries for sequencing: one enriched for full-length transcripts, and a second enriched for shorter transcripts (*e.g.* sRNAs and stable RNA degradation products).

### Antisense RNAs are abundant in the streptomycetes, and are largely species-specific

Previous RNA-Seq analyses in diverse bacterial species have revealed extensive asRNA expression [15,23]. Consistent with these observations, we detected abundant asRNAs in all three *Streptomyces* species: 680, 592 and 536 asRNAs were identified in *S. coelicolor, S. venezuelae* and *S. avermitilis,* respectively. These asRNAs could be further subdivided into three categories, on the basis of their coverage profiles and their genomic context: (i) asRNAs expressed from a dedicated promoter within a protein-coding gene (referred to here as simply ‘asRNAs’); (ii) asRNAs that arose from the overlap of 3′ untranslated regions (UTRs) from convergently oriented genes, an RNA species that we have termed ‘cutoRNA’ (see below); and (iii) asRNAs that resulted from divergent transcription, where promoters of divergently expressed genes overlapped (Additional file 1: Table S1).

asRNAs expressed on the strand opposite that of a protein-encoding gene [Class (i)], did not comprise a majority of the asRNAs identified here, with fewer than 100 identified in any of the three species. As has been observed for comparative analyses conducted in other bacteria [24], the majority of the 99 (*S. coelicolor*), 59 (*S. avermitilis*), and 79 (*S. venezuelae)* asRNAs identified were species specific (Additional file 1: Table S1). We considered the possibility that this species specificity resulted from asRNA association with coding sequences confined to a single species. This turned out not to be the case: 129 broadly conserved genes (genes with homologues in all three species) were associated with asRNAs in at least one species, but only 11 (or 8.5%) of these genes exhibited antisense expression in all three species (Table 1). This level of asRNA conservation is slightly less than that reported for *E. coli* and *Salmonella,* where ~14% of antisense transcripts were conserved between species [24]. Within bacteria, the regulatory impact of apparently unique asRNAs encoded opposite conserved open reading frames remains to be elucidated.

**Table 1:** Homologous genes with conserved asRNAs

***S. coelicolor***	***S. avermitilis***	***S. venezuelae***	**Predicted product of sense gene**
*sco2364*	*sav5807*	*sven2178*	Conserved hypothetical protein
*sco2685*	*sav5363*	*sven2472*	Putative ATP-binding protein
*sco3318 - sco3317*	*sav4741 - sav4740*	*sven3179 - sven3180*	Putative porphobilinogen deaminase (HemC) / uroporphyrinogen-III synthase (HemD)
*sco3408*	*sav4662*	*sven3260*	D-Ala-D-Ala carboxypeptidase
*sco3671*	*sav4484*	*sven3433*	DnaK - heat shock protein
*sco4137*	*sav4077*	*sven3895*	Pit-accessory protein (phosphate transport)
*sco4440*	*sav3782*	*sven3077*	Hypothetical protein
*sco4566 - sco4567*	*sav4841 - sav4842*	*sven4269 - sven4270*	NuoF / NuoE
*sco4606 - sco4607*	*sav4888 - sav4889*	*sven4315 - sven4316*	NuoL2 / NuoM2
*sco4829*	*sav3433*	*sven0126*	Putative oxidoreductase
*sco4848*	*sav3412*	*sven4521*	Putative integral membrane protein

**Figure 2:**
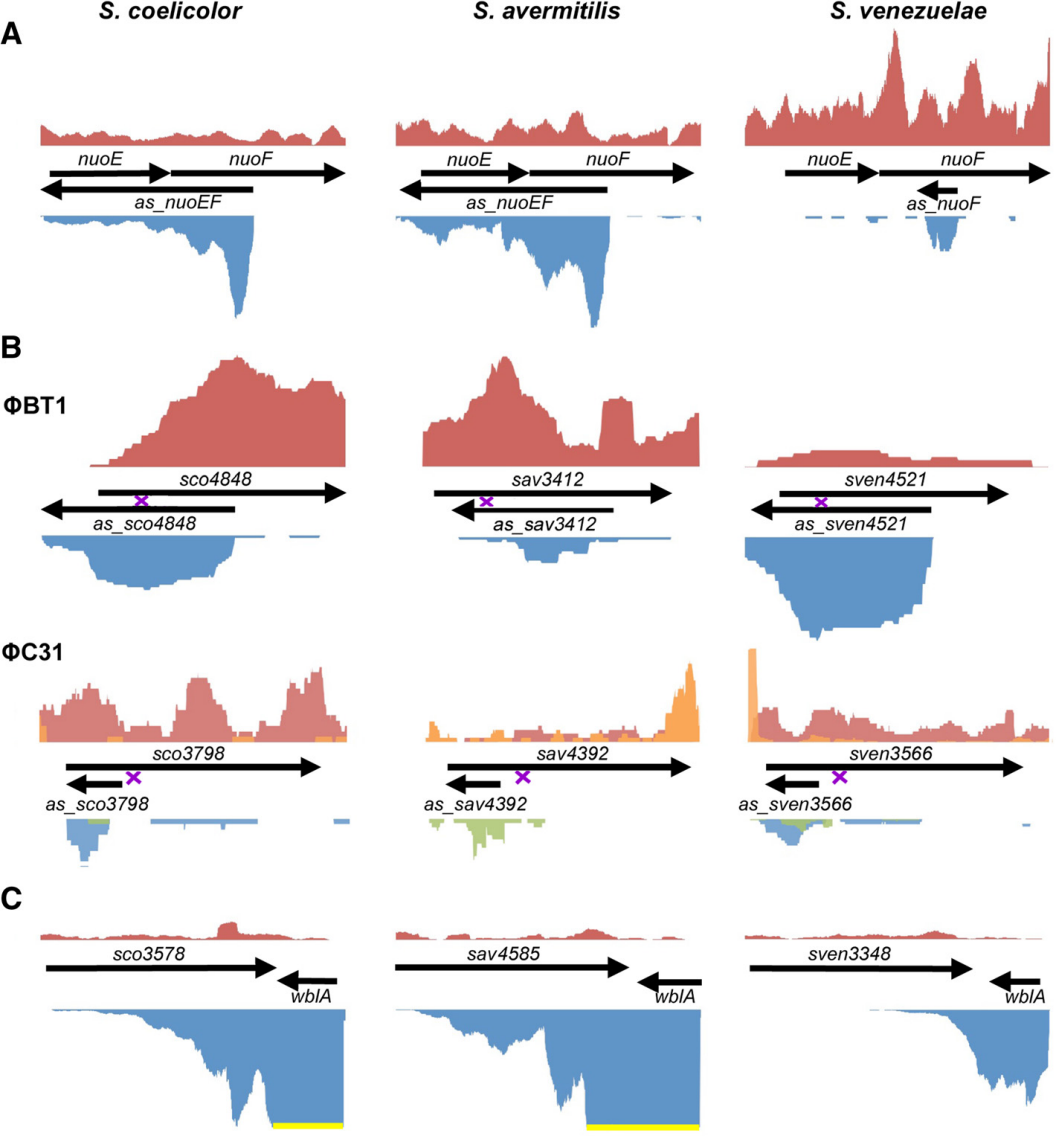
**Expression profiles of select conserved *cis*-antisense RNAs and cutoRNAs. (A)** Expression profile of the antisense gene opposite *nuoE* and *nuoF*. **(B)** Expression profile of a conserved asRNA opposite homologous genes in *S. coelicolor, S. avermitilis* and *S. venezuelae* which encompassed the site of ΦBT1 (Top) or ΦC31 (Bottom). The site of phage integration is marked with a purple ‘x’. **(C)** cutoRNA shared between *wblA* and a conserved downstream gene (*sco3578*/*sav4585/sven3348*). Red graphs (top) represent relative read coverage (long transcript library) for the positive strand (orange is the equivalent for the sRNA-enriched library), while blue graphs represent read coverage (long read library) for the negative strand (green represents the same in the sRNA-enriched library). As expression levels of different genes varied over several orders of magnitude, the y-axis for each gene set was scaled independently. When expression levels differed greatly for the positive and negative strand within a gene pair, the profile of the more highly expressed gene was cut off with a yellow line, to ensure that expression from the less highly expressed gene could be visualized.

Of the 11 conserved asRNAs we identified, the most striking was found opposite the *nuo* gene cluster. These *nuo* genes direct the expression of NADH:quinone oxidoreductase, an enzyme complex found in archaea, bacteria, and within eukaryotic mitochondria and chloroplasts [25]. This multi-protein complex, also known as complex I, is a key player in the respiratory transport chain [26]. Many bacteria encode a 14-subunit (NuoA-N) version of complex I; however, some groups have retained an ancestral 11-subunit form that lacks the ‘N-module’ subunits NuoE, NuoF and NuoG, while others have a 12-membered complex lacking only NuoE and NuoF [27]. It is within the N-module-encoding region that we identified one of the most highly expressed and conserved asRNAs. Transcription of the asRNA began within the coding region of *nuoF* and continued through the coding region of *nuoE* (Figure 2A), with the asRNA extending for up to 1,600 nucleotides in *S. coelicolor* and *S. avermitilis*; a shorter asRNA was observed in *S. venezuelae*. An intriguing possibility is that the asRNA provides a checkpoint in complex I assembly, down-regulating the expression of N-module-encoding genes until the rest of the complex has been synthesized/assembled. In order for such regulation to occur, both sense and antisense transcripts would need to be coordinately expressed. To test this, we conducted semi-quantitative RT-PCR experiments, and found that both sense and antisense transcripts were expressed at the same time (Additional file 1: Figure S1), supporting a possible regulatory role for this asRNA.

*Streptomyces* species also possess an additional copy of many of the complex I genes (*nuoA2*, *B2*, *D2*, and *H2* to *N2*) encoded from a disparate chromosomal location. Like the standard *nuo* gene cluster, these genes are organized contiguously (with the exception of *nuoD2)* and our data suggest that they are expressed as a single operon. While this second cluster lacked the N-module-encoding genes, it was associated with a second conserved asRNA extending from *nuoM2* to *nuoL2.* (Additional file 1: Figure S1). Both *nuoM2* and *nuoL2* encode antiporter-like proteins [28]. In the cyanobacterium *Synechocystis*, different antiporter subunits can be incorporated into complex I for different tasks related to photosynthesis [25,29,30]. The presence of additional *nuoL* and *nuoM* genes in *Streptomyces* genomes means there is the potential for analogous differential incorporation of these gene products into complex I, and this incorporation could be controlled by conserved asRNA activity. As for the *nuoEF-*associated asRNA, semi-quantitative RT-PCR revealed similar expression patterns for both *nuoL2* and its cognate asRNA, although the latter appeared to be expressed at lower levels relative to the mRNA (Additional file 1: Figure S1). Intriguingly, an asRNA has been reported opposite the *nuoM* homologue in rat mitochondria [31], raising the possibility that this asRNA arose before the evolution of eukaryotes, over 2-billion years ago.

In addition to the conserved asRNAs associated with the *nuo* gene clusters, we also identified conserved asRNAs associated with the genes targeted by the lysogenic phage ϕBT1 [32] and ϕC31 [33], although for the ϕC31-targeted genes, only the *S. venezuelae*-associated sequence met the relatively stringent cut-off we used in assigning asRNA designations. ϕBT1 integrates into the coding sequence of an integral membrane protein-encoding gene (*sco4848*/*sav3412*/*sven4521)*, while ϕC31 targets the coding sequence of a conserved pirin-like protein (*sco3798*/*sav4392*/*sven3566*). An asRNA encompassed the ϕBT1 integration site in all three *Streptomyces* species (Figure 2B), while for the ϕC31-associated genes, the asRNA was found immediately adjacent to the phage integration site (Figure 2B). There are a number of intriguing functional possibilities that could be ascribed to these asRNAs. They may simply act to control their associated protein coding genes, or they may contribute to a novel phage resistance mechanism, perhaps minimizing phage integration by sequestering these regions into transcriptionally active complexes. Alternatively, phage integration at these sites may be the result of positive selective pressure, as ϕC31, and presumably ϕBT1, integrate in an antisense orientation such that the integrase promoter is separated from its coding sequence [32,33]. As integrase activity is required for phage excision, a productive infection could only be achieved with the assistance of an endogenous (bacterial) promoter. For ϕBT1, such a promoter could obviously be provided by the asRNA (Figure 2B); for ϕC31, the asRNA promoter may well lie upstream of the integration site, but asRNA transcript levels were more abundant downstream of this region (Figure 2B).

### ‘cutoRNAs’ are a common and well-conserved phenomenon in *Streptomyces* species

In addition to the Class (i) asRNAs, we also identified a second major class of asRNAs in all three *Streptomyces* species, termed ‘cutoRNAs’, for convergent untranslated overlapping RNAs. These RNAs arose from the expression of convergent genes, whereby the transcription of one or both genes extended beyond its respective coding sequence into the downstream coding regions (Additional file 1: Table S1). Whilst we identified only 11 conserved asRNAs, there were 19 cutoRNA pairs conserved in *S. avermitilis, S. coelicolor* and *S. venezuelae* (Additional file 1: Table S2). We examined the genetic organization of these 19 gene pairs in other streptomycetes, and found this organization to be highly conserved. For example, in *Streptomyces scabies* and *Streptomyces griseus*, a convergent configuration was observed for 19/19 (*S. scabies*) and 18/19 (*S. griseus*) gene pairs. We extended our analyses to include more diverse actinobacteria, but found many of the genes involved were *Streptomyces-*specific; only the *wblA-sco3578* gene pair was conserved and convergently arranged in the more distantly related *Frankia alni, Thermobifida fusca*, and *Mycobacterium tuberculosis.* In *M. tuberculosis,* ‘antisense RNAs’ to both genes have been previously reported [34], suggesting broad cutoRNA conservation across the actinobacteria for this gene pair.

Given the extent of its conservation, we sought to further investigate the expression of the *wblA* and *sco3578* cutoRNA. *wblA* encodes a transcription factor that impacts both antibiotic production and aerial morphogenesis in *S. coelicolor*[35], while *sco3578* encodes a putative ion-transporting ATPase. Our RNA-Seq data revealed that the 3′ UTR of *wblA* covered the entire coding region of the downstream ATPase-encoding gene in both *S. coelicolor* (Figure 2C) and *S. avermitilis* (Figure 2C), extending more than 1.2 kb beyond the *wblA* translation stop site. In *S. venezuelae, wblA* transcripts extended ~500 nucleotides beyond the *wblA* coding sequence, well into the downstream coding sequence (Figure 2C). While the ATPase-encoding gene was expressed at much lower levels than *wblA,* its 3′ UTR still extended into *wblA*. Semi-quantitative RT-PCR analyses were conducted to follow the expression of these genes. We found each gene and its corresponding 3′ UTR, was expressed throughout development (Additional file 1: Figure S2). This suggested that, as for the asRNAs examined here, there is the potential for base pairing of these convergent transcripts, with possible downstream regulatory implications.

Outside of the *wblA-*associated cutoRNA, *M. tuberculosis* has previously been shown to have abundant asRNAs arising from the transcriptional read-through of convergently transcribed genes [34]. A similar phenomenon has also been noted in the more distantly-related (Gram-positive) bacterium *Bacillus subtilis*[36], suggesting that cutoRNAs may be widespread in bacteria. Studies in *B. subtilis* have also revealed intriguing correlations between flexible transcription termination and growth conditions [36]. It will be interesting to see whether cutoRNA occurrence in the streptomycetes is similarly impacted by different growth conditions.

There are a number of different scenarios by which cutoRNAs could function in the cell. Simultaneous expression of cutoRNA gene pairs could lead to altered stability of one or both transcripts. This is supported by an analysis of recently published data comparing gene expression in wild type and RNase III deficient strains of *S. coelicolor*[37] (where RNase III specifically cleaves double stranded RNA), which revealed that one gene in each of seven different cutoRNA pairs was significantly impacted by the loss of RNase III (*SCO1150, SCO4283, SCO4749, SCO5106, SCO5146, SCO6716,* SCO6729; Additional file 1: Table S1). cutoRNAs could also serve to ‘tether’ the convergently expressed mRNAs such that their protein products are produced in close proximity. This would imply a functional correlation between the convergent genes and their resulting products. Currently, there is no experimental evidence supporting related functions for any of the conserved cutoRNA gene pairs, as the majority of these genes have not been characterized. It is worth noting, however, that cutoRNAs were abundant in the species-specific secondary metabolic gene clusters, where they were shared between genes with obvious functional relationships (Additional file 1: Table S1 and below).

In *E. coli,* cutoRNA-like transcription is thought to be deleterious, and it has been proposed that the Rho transcription termination factor acts to prevent such asRNA expression [38]. Rho activity can be inhibited by the antibiotic bicyclomycin, and studies in a close relative of *S. coelicolor*, *Streptomyces lividans,* have revealed that bicyclomycin has no effect on colony growth [39], suggesting that the loss of Rho activity is not detrimental to the streptomycetes. This may imply that *Streptomyces* tolerate convergent transcription better than *E. coli*, or it may mean that they invoke other, as yet unknown means of dealing with transcriptional conflicts caused by convergent transcription.

Of the remaining asRNAs identified, very few were the result of divergent expression from overlapping promoters (five were observed in *S. coelicolor,* while none were detected in *S. avermitilis* or *S. venezuelae*) (Additional file 1: Table S1 ). Instead, much of the antisense transcription we detected could not be readily categorized (Additional file 1: Table S1 ). This was largely due to the lack of defined transcription start/stop sites and uneven transcript coverage, which made definitive classification challenging. It is conceivable that many of these transcripts were processed shortly after generation, possibly in conjunction with their corresponding sense transcripts, and consequently full-length asRNAs failed to accumulate. The idea that rapid processing masks the full extent of antisense transcription has been supported by findings in *Staphylococcus aureus,* where full length asRNAs were detected only following RNase III depletion [40]. The number of genes with associated asRNAs in *Streptomyces* may therefore be much higher than reported here.

### Expanding the *Streptomyces* sRNA landscape: conservation and organization of new sRNAs

**Figure 3:**
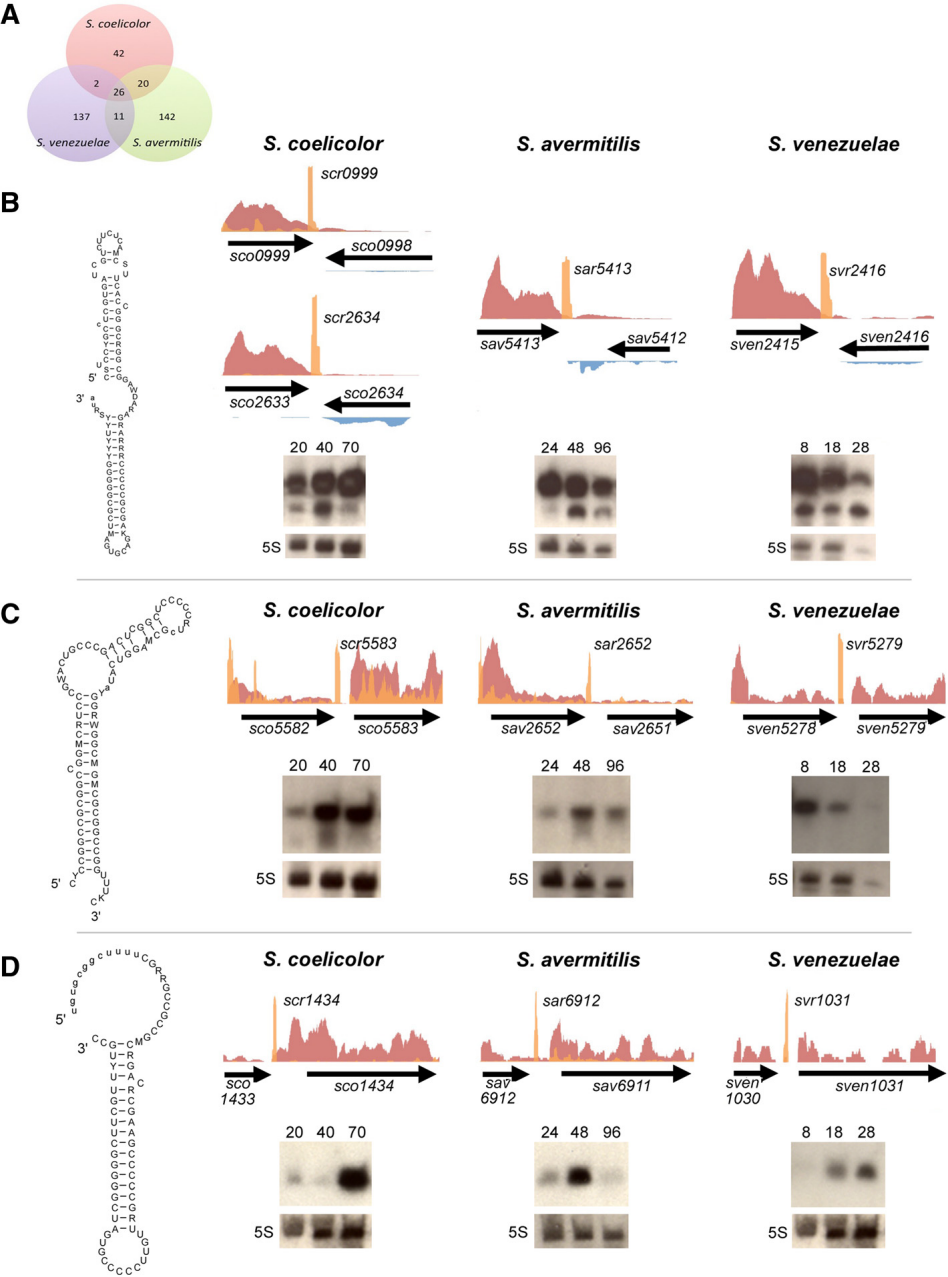
**Comparing conserved intergenic sRNAs: structure and expression analyses. (A)** Venn diagram illustrating sRNA conservation in *S. coelicolor*, *S. avermitilis* and *S. venezuelae*. **(B-D)** Structure, expression profiles and northern blot analyses of conserved sRNAs: **(B)** scr0999, scr2634, sar5413 and svr2416; **(C)** scr5583, sar2652 and svr5279; **(D)** scr1434, sar6912 and svr1031. For **(B-D)**: predicted conserved secondary structures for each sRNA are shown to the far left. Non-standard bases are indicated as follows: M (A or C), R (A or G), W (A or U), S (G or C), Y (C or U), K (G or U), D (not C). Insertions are denoted in lower case. For the expression profiles: positive strand coverage is shown above the gene annotation in red (long transcript library) and orange (sRNA-enriched library); negative strand profiles (below annotation) are shown in blue (long transcript library) and green (sRNA-enriched library). Northern blots showing the temporal expression of each sRNA (time of RNA extraction is indicated in hours) are shown below each coverage graph. For each blot, 5S rRNA was also probed as a control for RNA integrity and abundance.

To expand the existing library of sRNAs in *S. coelicolor*, and to begin to understand the distribution of sRNAs in different *Streptomyces* species, we endeavoured to mine our RNA-Seq data for unannotated sRNA genes within the intergenic regions of *S. coelicolor*, *S. avermitilis* and *S. venezuelae* (Figure 3A). New sRNAs were given a designation that consisted of a species reference, followed by a number corresponding to that of its right flanking protein-coding gene (*e.g. scr1434/sar6912/svr1031* for *S. coelicolor, S. avermitilis* and *S. venezuelae* sRNAs, respectively) (Additional file 1: Table S3). We identified 90 sRNAs in *S. coelicolor*, of which 71 were novel, bringing the total number of confirmed sRNAs in *S. coelicolor* to 105. Interestingly, we detected greater numbers of sRNAs in *S. avermitilis* and *S. venezuelae*: 199 and 176, respectively, of which fewer than 20 in each species were homologous to previously identified sRNAs from *S. coelicolor*. We also observed 17 of 34 previously confirmed sRNAs from *S. coelicolor*[17,18,21], along with another four that had been predicted but not experimentally validated [17,18] (Additional file 1: Table S3). An additional 12 previously confirmed/predicted sRNAs appeared, from our data, to be highly expressed 5′ UTRs and not independently encoded sRNAs. This did not, however, preclude these regions from having sRNA regulatory potential, as there are documented examples of functional sRNAs arising from transcription attenuation within 5′ UTRs [41].

Unlike the asRNAs, we found a significant number of intergenic sRNAs were conserved between the three species (Figure 3A; Additional file 1: Table S4). Of the 92 sRNAs we identified in *S. coelicolor*, 28.7% were conserved at a sequence level (E-value less than 1e-06) in all three species, while 22.3% and 2.2% were shared with *S. avermitilis* or *S. venezuelae,* respectively. We considered the possibility that some these conserved sRNA genes may - in addition, or alternatively – encode a small protein, as has been seen in *E. coli*[42]. We scrutinized all conserved sRNA sequences for open reading frames that were also conserved between species, and found four of 58 with the potential to encode a conserved protein (Additional file 1: Table S4). Further experimentation will be needed to assess the protein-coding capacity of these four genes.

Here, we directed our efforts towards the initial characterization of a number of highly expressed, non-protein-coding novel sRNAs. Using northern blotting, we probed the expression of three conserved sRNAs to verify our RNA-Seq data and to investigate their expression profiles. One of the most highly-expressed conserved sRNA had two equivalently expressed paralogues in *S. coelicolor* (*scr2634*, *scr0999*) (Figure 3B). In *S. avermitilis* and *S. venezuelae,* the equivalent sRNAs (*sar5413* and *svr2416,* respectively) were also highly expressed (Figure 3B). Structural predictions suggested that these sRNAs adopted near identical structures (Figure 3B), being largely unaffected by primary sequence differences. In each species, the sRNA was expressed from a site immediately downstream of *sodF* (within 18 nucleotides), where *sodF* encodes an iron/zinc superoxide dismutase involved in the defense against reactive oxygen species. While *sodF-*associated sRNAs have not been reported previously, sRNAs encoded within the 3′ regions of protein-coding genes are not unprecedented and have been described recently in *Salmonella*[43]. There is, however, evidence for control of *sodF*-like genes by small RNAs: expression of the *sodF* equivalent in *E. coli*, *sodB*, is controlled by the RyhB sRNA [44]; we do not currently have any data supporting a regulatory connection between *sodF* and the associated downstream sRNA. Northern blot analysis revealed that this *sodF-*associated sRNA was expressed throughout development in all three *Streptomyces* species (Figure 3B).

We probed an additional conserved sRNA that was amongst the most highly expressed in all three species*.* scr5583, sar2652*,* and svr5279 shared extensive sequence identity, and were predicted to have a structurally distinctive C-rich (67%) terminal loop (Figure 3C). Many well-characterized sRNAs, such as RNAIII in *S. aureus*[45], target mRNAs via C-rich loops; however, *Streptomyces* genomes are very GC-rich (>70%), so whether an equivalent phenomenon exists in these bacteria remains to be seen. Unexpectedly, northern blot analyses revealed that this sRNA was differentially expressed in three *Streptomyces* species: it was expressed most highly during aerial hyphae formation and sporulation (later developmental stages) in *S. coelicolor* and *S. avermitilis,* whereas in *S. venezuelae*, it was most highly expressed during vegetative (early) growth (Figure 3C).

Finally, we examined the expression profiles of the highly expressed scr1434, sar6912, and svr1031 sRNAs. Highest levels of each, as determined by northern blotting, were observed during aerial hyphae formation and sporulation (Figure 3D). This sRNA was predicted to form a very stable stem-loop structure, again, having a C-rich loop region (Figure 3D).

**Figure 4:**
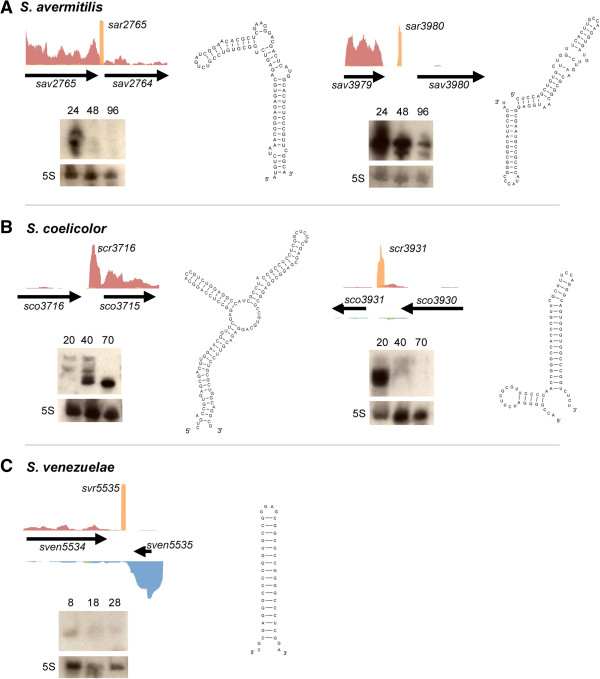
**Structure and expression analyses of species-specific intergenic sRNAs.** Expression profiles, northern analyses and structural predictions for: **(A)***S. avermitilis* sRNAs sar2765 (left) and sar3980 (right); **(B)***S. coelicolor* sRNAs scr3716 (left) and scr3931 (right); **(C)***S. venezuelae* sRNA svr5535. For each expression profile, relative sequence reads for genes encoded on the positive strand (top) are shown in red (long transcript library) and orange (sRNA-enriched library), while negative strand profiles are shown in blue (long transcript library). As expression levels for different genes varied greatly, the y-axes of each panel were scaled independently. Northern blots, shown below each coverage graph, revealed sRNA expression throughout development (time of RNA harvest is shown in hours). 5S rRNA was probed as a control for RNA quantity and integrity.

While many sRNAs were shared by all three *Streptomyces* species, there were notable species-specific differences as well. We focused our attention on select highly expressed unique sRNAs, and used northern blot analysis to assess their expression profiles (Figure 4). Within *S. avermitilis,* the 89 nucleotide sar2765 was expressed exclusively during vegetative growth (Figure 4A), while the equivalently sized sar3980 (88 nucleotides) was expressed most highly during vegetative and aerial growth (Figure 4A). In *S. coelicolor,* scr3716 (~128 nucleotides) was highly represented in our long transcript-enriched library and was not present in the sRNA-enriched library, unlike the majority of sRNAs identified in our study (this is in contrast to all classes of asRNA, which were almost exclusively detected in our long transcript library). scr3716 was expressed at low levels during vegetative growth, with expression levels rising significantly during aerial development and sporulation (Figure 4B), in contrast to the smaller 70 nucleotide scr3931, which was expressed solely during vegetative growth (Figure 4B). In *S. venezuelae,* svr5535 was one of the shortest sRNAs identified in our study at only 41 nucleotides, and unlike many other sRNAs, it was expressed throughout development (Figure 4C). Apart from svr5535, which was predicted to form a single stem-loop structure, all other sRNAs were predicted to adopt two or three stem-loop configurations.

In considering species-specific versus conserved sRNAs, we explored whether any correlation could be drawn between conservation and genome position. *Streptomyces* chromosomes are unusual relative to those of most bacteria in that they are linear, and are organized such that there is a central ‘core’ region that is broadly conserved in all actinobacteria. This central core is flanked on either side by ‘arm’ regions whose sequences are more divergent. Comparative genomic analyses have suggested that the left arm contains an actinomycete-specific region immediately adjacent to the core, while the equivalent position in the right arm is associated with *Streptomyces*-specific genes. The extreme ends of the chromosome arms contain predominantly species-specific genes [46]. We examined the position of each sRNA in *S. coelicolor* in relation to these different genetic bounds (Table 2). The majority of sRNAs (58 of 92) fell within the core region, with 50% of these conserved in at least one of the other two *Streptomyces* species. Of the 17 sRNAs located in the ‘actinomycete-specific’ region, a remarkable 82% were conserved, whereas somewhat surprisingly, only eight sRNAs were expressed from within the ‘*Streptomyces*-specific’ region, and of these, only three were also found in *S. avermitilis* or *S. venezuelae*. In the divergent chromosomal ends, few sRNAs were identified, and all of these were unique to *S. coelicolor*.

**Table 2:** **Location of unique and conserved sRNAs in *S. coelicolor***

Chromosomal region^*^	**# sRNAs**	**# unique**	**# conserved**	**% conserved**
Left terminal region	2	2	0	0%
Left *Actinomycetales*-specific region	17	3	14	82.4%
Core region	58	29	29	50.0%
Right *Streptomyces*-specific region	8	5	3	37.5%
Right terminal region	7	7	0	0%

* as per Kirby et al. [46].

In general, the 105 sRNAs identified here and elsewhere [17,18,21] for *S. coelicolor* is comparable to the number of sRNAs detected in *E. coli* (currently estimated to be ~80 [47]). This is fewer than might have been expected given the large *Streptomyces* genome (>8 Mb versus 4–5 Mb for *E. coli*), and the relatively large proportion of protein-encoding genes dedicated to regulation in *S. coelicolor* (12.3% of all protein-coding genes [48]). It is likely, however, that sRNA saturation has not been reached in any *Streptomyces* species, given that there has yet to be an exhaustive search conducted using different growth and stress conditions, and that each investigation undertaken to date has identified unique sRNA subsets without considerable overlap.

### ncRNAs feature prominently in many secondary metabolite clusters

*Streptomyces* species are renowned for their ability to produce a broad range of antibiotics, together with a host of other secondary metabolites having medical and agricultural utility. Our transcriptome analyses have revealed previously unrecognized complexity for some secondary metabolic clusters, largely in the form of asRNA expression.

**Figure 5:**
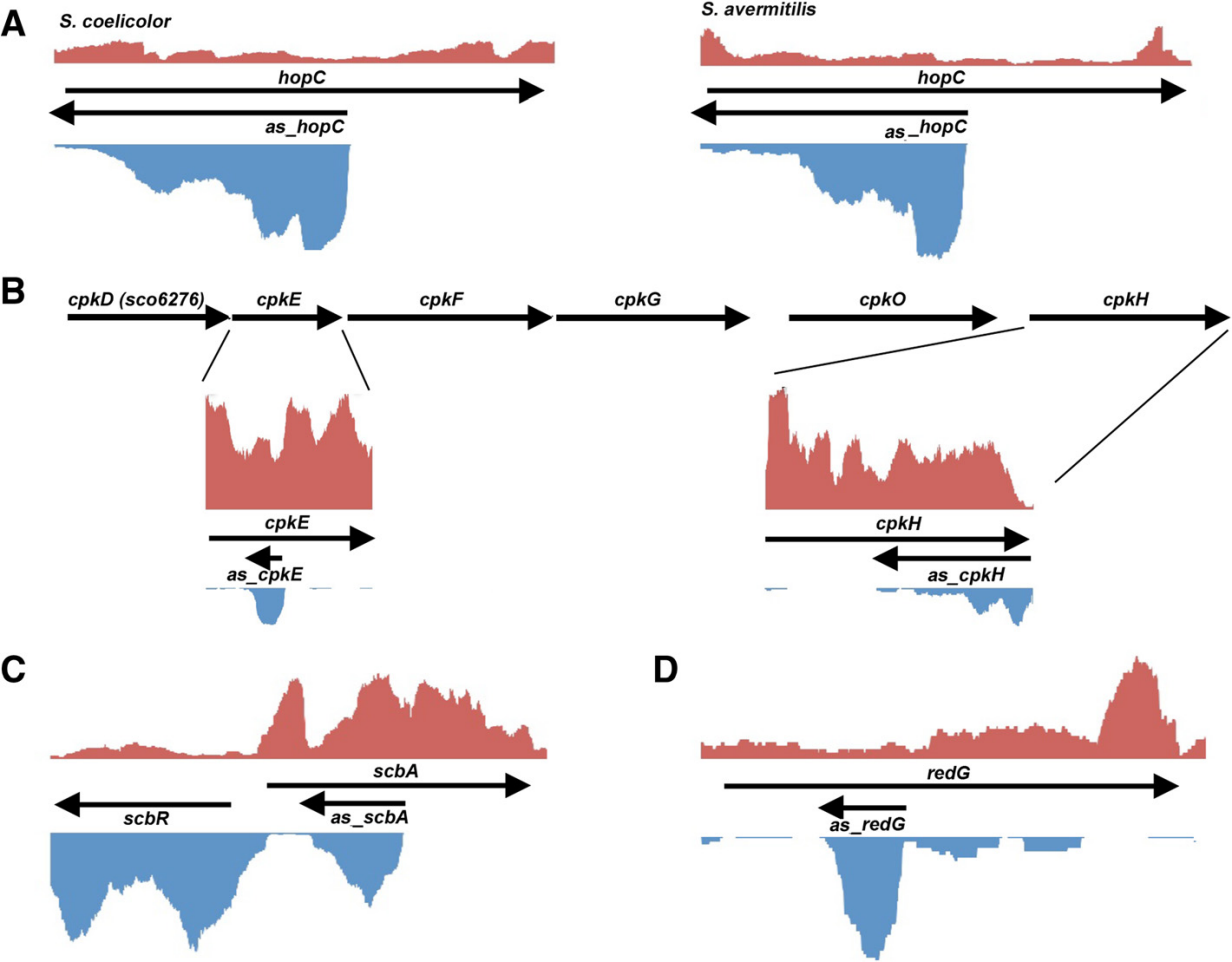
**Expression profiles of antisense RNAs within secondary metabolite clusters. (A)** Expression profile of the asRNA expressed opposite *hopC* in *S. coelicolor* (left) and *S. avermitilis* (right). **(B)** Expression of the two asRNAs expressed opposite *cpkE* and *cpkH* within the coelimycin P1 biosynthetic cluster of *S. coelicolor*. The relative position of these two genes within the cluster is shown above the coverage graphs. **(C)** Expression levels for *scbA* and *scbR* within the coelimycin P1 biosynthetic cluster in *S. coelicolor*. Antisense RNAs resulted from the divergent transcription of these two genes [60] and an independent antisense RNA was expressed within the coding region of *scbA*. **(D)** Expression profile of the asRNA expressed opposite *redG* within the prodiginine biosynthetic cluster of *S. coelicolor*. For each of **(A-D)**, relative sequence reads at each nucleotide position were shown in red (positive strand on the top), and blue (negative strand on the bottom). The y-axis of each gene set was scaled independently, as expression levels of different gene clusters varied.

asRNAs were abundant in the predicted secondary metabolic clusters for the three *Streptomyces* species examined here: 20% of *S. avermitilis*, 30% of *S. coelicolor* and 60% of *S. venezuelae* secondary metabolic clusters were associated with asRNAs of at least one type (Additional file 1: Table S1). Given the lack of general antisense RNA conservation found both within the streptomycetes in this study, and in other bacteria [49], we were surprised to identify a strongly-expressed *cis*-antisense RNA within a hopanoid biosynthetic cluster in *S. coelicolor* and *S. avermitilis* (Figure 5A). Hopanoids are cholesterol-like pentacyclic molecules [50-52] found throughout bacteria [53]. In *S. coelicolor*, the 12 gene hopanoid biosynthetic cluster is most highly expressed during aerial development, and it has been proposed that hopanoids help promote water retention during aerial hyphae formation [54]. This may explain why the equivalent cluster in *S. venzeuelae* (grown in liquid culture) was expressed at very low levels. The asRNA was transcribed opposite *hopC* (*sco6762)* (Figure 5A), a predicted phytoene dehydrogenase-encoding gene. Using semi-quantitative RT-PCR, we determined that both sense and antisense genes were expressed at the same time (Additional file 1: Figure S1). The hopanoid cluster in *S. coelicolor* is thought to direct the synthesis of both hopene and the related aminotrihydroxybacteriohopane [54]. Little is known about the biosynthetic steps leading to the synthesis of either compound, and nothing is known about the role of HopC. It is possible that *hopC* expression may be modulated by its cognate asRNA, which in turn could impact the production of one or both of these products.

Two well-characterized secondary metabolic clusters in *S. coelicolor* also encoded distinct antisense RNAs: the coelimycin P1 (*cpk*) biosynthetic cluster (Figure 5B,C) and the prodiginine (*red)* biosynthetic cluster (Figure 5D). The 16 gene coelimycin P1 biosynthetic cluster (*sco6273-6288*) [55-57] includes two genes with associated asRNAs: *cpkE/sco6277* (encoding a putative epoxide hydrolase) and *cpkH/sco6281* (encoding a putative FAD-binding protein). The *cpkE-*associated asRNA was expressed most highly in the centre of *cpkE*, while the *cpkH* antisense was expressed closer to the 3′ end of the coding sequence (Figure 5B). The roles of CpkE and CpkH in coelimycin P1 biosynthesis have yet to be elucidated. It is worth noting that *cpkE* is expressed as part of a larger operon (*cpkD-G*), and that the expression of this entire operon was increased by more than two-fold in an RNase III mutant strain [37], suggesting that the *cpkE* asRNA may function to destabilize its cognate polycistronic mRNA in an RNase III-dependent manner. In contrast, *cpkH* expression was not enhanced following the loss of RNase III, although transcript levels for both upstream (*cpkO*) and downstream flanking genes (*cpkI-K*) were increased [37], suggesting complex post-transcriptional dynamics in this area.

Regulators of *cpk* gene expression (ScbA and ScbR) also appeared to be subject to asRNA regulation (Figure 5C). ScbA directs the synthesis of the ϒ-butyrolactone quorum sensing molecule SCB1, which is sensed by ScbR - an SCB1 receptor/DNA binding transcription factor that represses *cpk* cluster expression in the absence of SCB1 [58]. It has been previously reported that the promoters - and thus 5′ UTRs - of the divergently transcribed *scbR* and *scbA* genes overlap [59,60] (Additional file 1: Table S1). In addition to the asRNAs resulting from this 5′ overlap, we also found there was a highly expressed asRNA within *scbA* (Figure 5C), adding an additional layer of regulation to an already transcriptionally intricate region.

The prodiginine cluster spans 22 genes and yields two major products: undecylprodigiosin and butyl-*meta*-cycloheptylprodigionine. RedG, a Rieske oxygenase-like enzyme, is thought to catalyze the conversion of undecylprodigiosin to butyl-*meta*-cycloheptylprodigionine [61], and it was opposite *redG* that a significant asRNA was detected (Figure 5D). The undecylprodigiosin:butyl-*meta*-cycloheptylprodigionine ratio is typically ~2:1 [62], so the *redG*-specific asRNA could provide a means of selectively modulating *redG* expression without impacting that of the downstream *redF*, whose product acts earlier in the undecylprodigiosin biosynthetic pathway [63].

**Figure 6:**
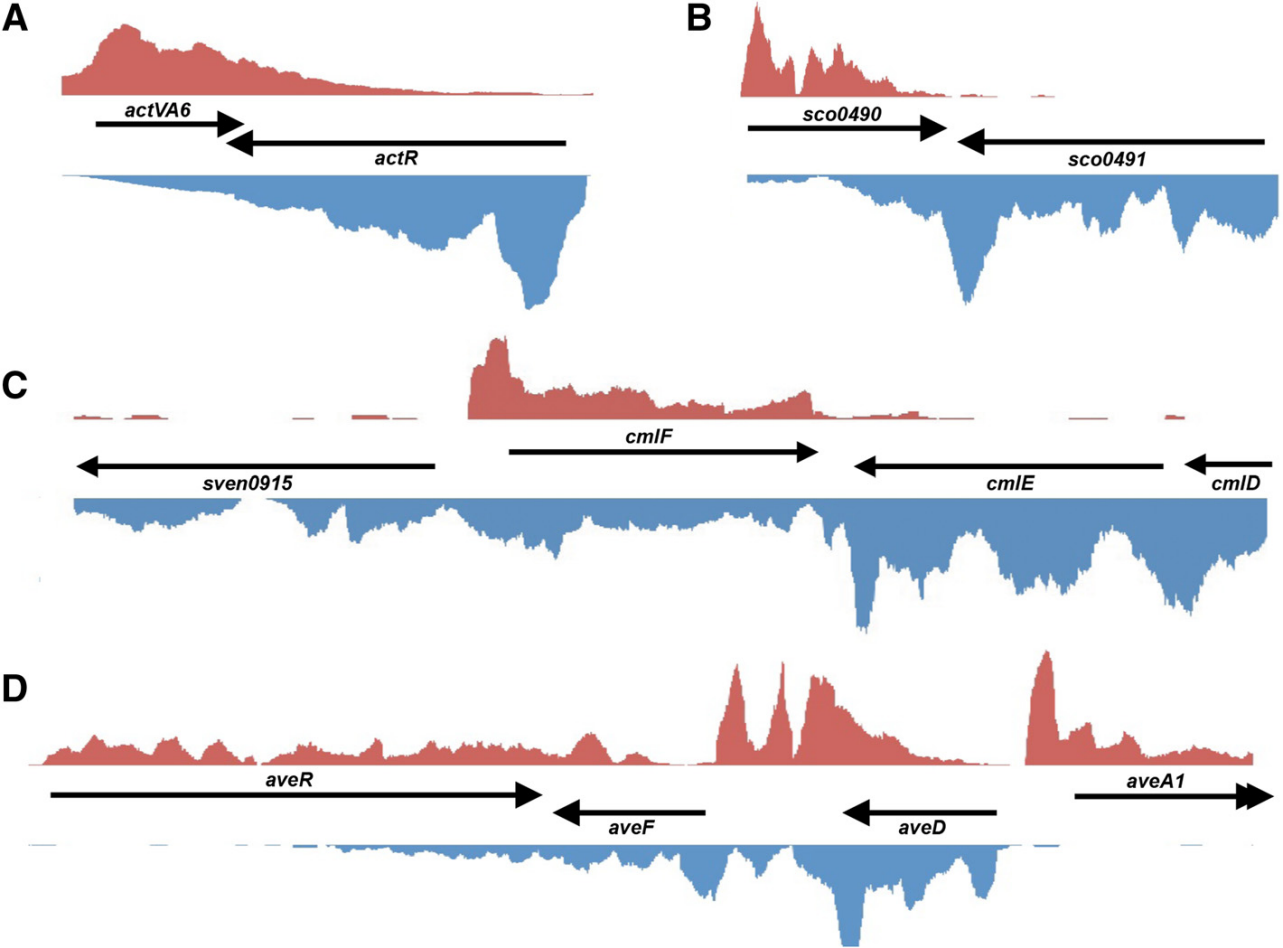
**cutoRNA expression within secondary metabolic clusters. (A)** Expression levels for a cutoRNA pair shared by *actVA6* and *actR* within the actinorhodin cluster of *S. coelicolor*. **(B)** Expression profiles for a cutoRNA pair (*sco0491* and *sco0490*) within the coelichelin siderophore biosynthetic cluster of *S. coelicolor*. **(C)** Expression of a four gene region within the chloramphenicol biosynthetic cluster of *S. venezuelae*. Transcription of the *cmlDE* operon generates an asRNA/cutoRNA to *cmlF*. **(D)** Expression profile of a transcriptionally complex region within the avermectin biosynthetic cluster of *S. avermitilis*. A cutoRNA pair exists between *aveR* and *aveF*. Multiple antisense RNAs were found opposite the *aveDF* operon. For **(A-D)**, relative sequence reads at each nucleotide position were shown in red for the positive strand (top), and blue for the negative strand (bottom). Given the differing expression levels observed for each gene cluster, y-axes for each panel were scaled independently.

In addition to expressing conventional asRNAs, secondary metabolic clusters were also a rich source of cutoRNAs, with eight (*S. coelicolor*), six (*S. avermitilis*) and three (*S. venezuelae*) cutoRNA pairs identified within these clusters (Additional file 1: Table S1). These included a distinctive cutoRNA pair within the 22 gene actinorhodin (*act*) biosynthetic cluster of *S. coelicolor*. Actinorhodin is a blue-pigmented polyketide antibiotic whose synthesis is directed by one of the best-studied pathways in the streptomycetes. At the centre of this cluster are two convergently transcribed genes, *actVA6* and *actR,* whose coding sequences overlap, and whose transcripts extended the full length of their respective downstream genes (Figure 6A)*. actVA6* encodes a monooxygenase that catalyzes an intermediate step in actinorhodin biosynthesis [64], while *actR* encodes a TetR-family repressor of the proposed actinorhodin resistance (ABC transporter) encoding genes *actAB*[65,66].

The intriguing genetic coupling of biosynthesis and transport-associated genes was also observed for the siderophore-producing coelichelin biosynthetic cluster [67]. Within this 11 gene cluster, the 3′ UTR of *sco0491* (*cchI*) extended into the coding region of *sco0490* (*cchJ*) (Figure 6B). Similar to the cutoRNA pair from the actinorhodin cluster, *sco0490* encodes a coelichelin biosynthetic enzyme, and *sco0491* encodes an ABC transporter that may participate in coelichelin export [67].

This theme was further reiterated in the chloramphenicol biosynthetic cluster of *S. venezuelae*. A four gene region encompassing *sven0915, cmlF*, *cmlE*, and *cmlD* (the leftmost genes in the cluster) was transcribed such that expression of the *cmlE-cmlD* operon failed to terminate, and instead extended through *cmlF* (a major facilitator family transporter) encoded on the opposite strand, into *sven0915,* located approximately 1,600 base pairs downstream (Figure 6C). CmlE and CmlD are required for chorismic acid synthesis, where chorismic acid is a precursor for both aromatic amino acid and chloramphenicol biosynthesis [68]; CmlF is a major facilitator transporter that may contribute to chloramphenicol resistance (although it is not the major resistance determinant).

Interestingly, while this cutoRNA phenomenon has not been previously reported, prior studies have identified short asRNA regions associated with antibiotic resistance genes, that stemmed from overlapping divergent promoters [69-71]. It will be interesting to determine whether such overlap of 5′ and 3′ untranslated regions is important for the stability and/or function of the associated mRNAs and protein products.

Most secondary metabolic clusters exhibited transcriptional patterns that could be readily correlated with protein-coding genes or defined asRNAs; however, there were clusters in each species that exhibited unusual transcriptional complexity. The most remarkable example of this was in the avermectin biosynthetic cluster of *S. avermitilis*. Avermectin is an important anti-parasitic agent used broadly in veterinary medicine [72]. Its biosynthetic cluster spans ~81 kb and 19 annotated genes [73,74], and an 18 kb region at the left end of the cluster encompassed a multitude of ncRNAs. This region included genes encoding the pathway-specific activator AveR, two polyketide tailoring enzymes (AveF and AveG), and the first of four polyketide synthases (the type I polyketide synthase AveA1). Expression of the convergently-oriented *aveR* and *aveF* genes resulted in the generation of a cutoRNA (Figure 6D). There was also very high antisense expression observed opposite *aveF* and *aveD,* which themselves appeared to be co-transcribed despite being separated by >700 bp (this unusual operonic structure has been noted previously [75]).

### mRNA-associated small RNAs are widespread in the streptomycetes

**Figure 7:**
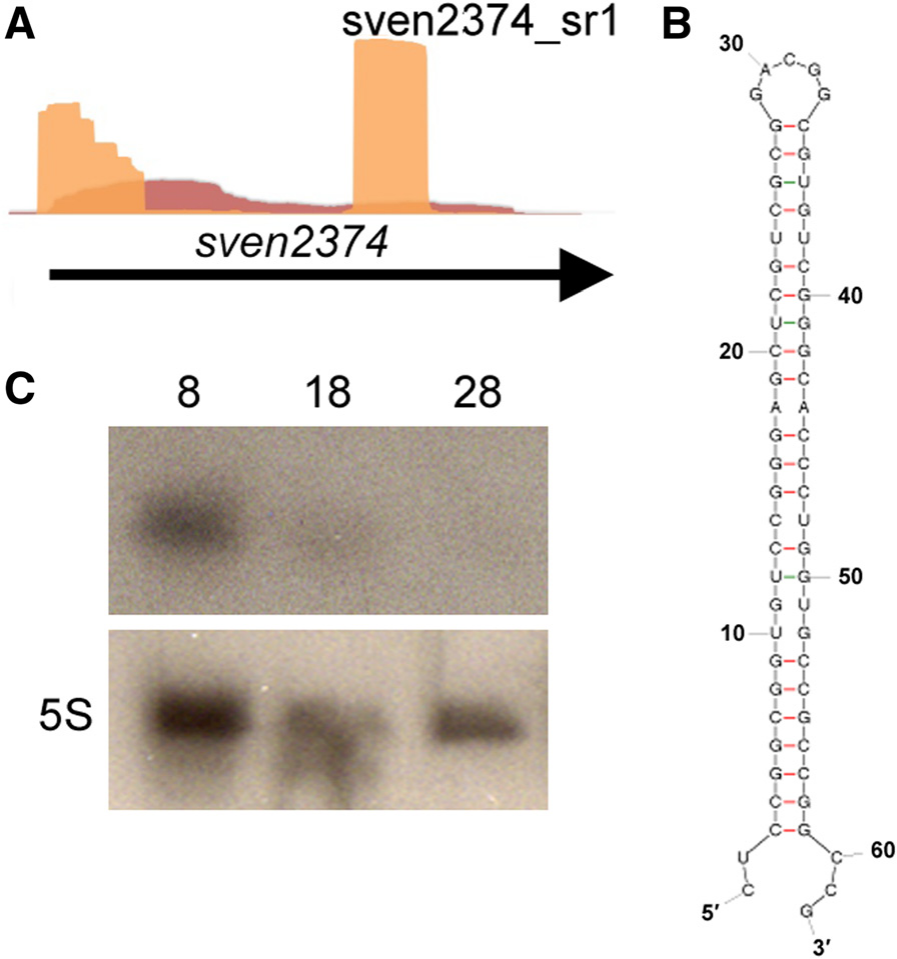
**Expression profile, northern analysis and secondary structure prediction of an mRNA-associated small RNA in *S. venezuelae*. (A)** Expression profile of *sven2374,* which shows high read levels for both its 5′ end and an internal 62 nucleotide region. Relative sequence reads at each nucleotide position are shown in red (long transcript library) and orange (sRNA-enriched library). **(B)** Predicted secondary structure of sven2374_sr1 (sr1: stable region 1). **(C)** Northern blot showing the temporal expression of sven2374_sr1 (time of RNA extraction is indicated in hours). 5S rRNA was probed as a control for RNA integrity and RNA loading levels.

The sRNA-enriched library proved to be a rich source of not only intergenic sRNAs, but also mRNA-associated short RNAs. While the long transcript libraries yielded relatively even coverage throughout most mRNAs, the sRNA-enriched libraries were dominated by sequences from the 5′ and 3′ UTRs of coding sequences. These regions were, in some instances, represented 100–1000 fold more highly than their corresponding coding sequences. In addition to these stable 5′ and 3′ UTR-associated RNAs, we also detected short sequences (~30-90 nucleotides) within coding regions that were unusually highly represented relative to their flanking sequences. A typical example of both end- and internally-enriched RNAs was seen for *sven2374*, which showed significant over-representation of its 5′ end and an internal 62 nt region, relative to the rest of the coding sequence (Figure 7A). Stable secondary structures within 5′ UTRs have been previously described [76-78] and can influence overall mRNA stability, whereas stable regions within 3′ UTRs may represent termination sequences (a class of sequence that has not been well-defined in the streptomycetes). In *E. coli,* 5′ and 3′ UTR fragments can accumulate to high levels, and in some cases appear to be expressed at times distinct from that of their corresponding mRNAs, suggesting that they may have the capacity to act as independent RNA molecules [79]. Similarly, in *Salmonella*, recent findings have revealed that 3′ UTRs have significant sRNA-encoding potential [43].

To begin understanding how these stable RNA species could be generated, we selected 20 of the most highly represented sequences for further analysis (Additional file 1: Table S5). These sequences were confined to *S. venezuelae*, where this phenomenon was more predominant than in the other two species examined. Secondary structure predictions [80] for the 20 highly represented sequences, suggested that all of these regions were highly structured, compared with sequences that were less abundant in the sRNA-enriched library (*e.g.* Figure 7B). We evaluated the GC content of these 20 sequences, comparing them with the nucleotide content of the entire coding sequence, and with a 15-nucleotide sequence window immediately upstream of the stable/structured sequences. We found the 20 structured sequences had a GC-content similar to that of the coding sequence as a whole. This is in contrast to the regions immediately preceding the structured sequences, which were significantly more AT-rich (an average of 32.3% versus 27.8%), and contained a higher proportion of poorly-used codons relative to the structured sequence immediately following. This suggested the potential for translational pausing, which when coupled with a highly structured downstream region, could promote stable RNA fragment accumulation. In *E. coli*, the major endoribonuclease RNase E cleaves in AU-rich regions near hairpin structures [81], and it will be interesting to see whether *Streptomyces* RNase E [82] contributes to the accumulation of these RNA species. It is equally possible that these RNA products were expressed independently of the associated coding sequence, as the AT-rich upstream region would also be consistent with a promoter region. To determine whether these abundant intragenic RNAs existed in the cell as discrete RNA elements, we used northern blotting with a probe specific for the highly represented region within *sven2374*. We observed a stable product during the early vegetative growth phase of *S. venezuelae* (Figure 7C), suggesting that these RNAs do indeed accumulate; whether they have a functional role in the cell remains to be determined.

## Conclusions

Using an RNA-Seq approach to evaluate gene expression throughout the *Streptomyces* life cycle, we have identified hundreds of novel ncRNAs in three disparate *Streptomyces* species. These included novel sRNAs, asRNAs and a prominent new class of asRNA – the cutoRNAs – that result from overlapping convergent transcription. Comparative analysis of the ncRNAs revealed considerable differences between species and between ncRNA types: *S. coelicolor* and *S. avermitilis* shared far greater numbers of ncRNA elements than either did with *S. venezuelae*, and throughout all species, asRNAs were less well conserved than sRNAs. From a genome-scale perspective, sRNA conservation largely mirrored that of protein-coding genes: sRNAs expressed from the chromosome ends were species-specific, while chromosome core-localized sRNAs were more highly conserved. Notably, ncRNAs were common features in secondary metabolic biosynthetic clusters, and likely contribute to the regulatory control of these pathways. Uncovering the ncRNA capacity of the streptomycetes will facilitate the downstream integration of these molecules into the regulatory networks governing growth, development and antibiotic production.

## Methods

### *Streptomyces* growth and RNA isolation

*Streptomyces* strains were grown on cellophane disks on the surface of solid MYM agar medium (*S. coelicolor* M145 and *S. avermitilis* MA-4680), or shaken in flasks containing liquid MYM (*S. venezuelae* ATCC 10712) at 30°C. Cells were harvested at timepoints corresponding to vegetative growth, aerial hyphae formation (or mycelial fragmentation in the case of *S. venezuelae)* and sporulation. RNA was extracted using a modified version of the guanidium thiocyanate protocol described previously [83]. Briefly, cells were lysed by vortexing with glass beads in a guanidium thiocyanate solution (4 M guanidium thiocyanate, 25 mM trisodium citrate dihydrate, 0.5% w/v sodium N-lauroylsarcosinate, 0.8% ß-mercaptoethanol) until homogeneous. The resulting suspension was subjected to two phenol-chloroform extractions and one acid phenol-chloroform extraction. Total nucleic acids were precipitated overnight at −20°C in isopropanol, before being pelleted, washed with 70% ethanol and resuspended in nuclease free water. Contaminating DNA was removed using Turbo DNase (Ambion), and RNA purity and concentration were determined using a Nanodrop spectrophotometer. RNA quality was assessed using an Agilent 2100 Bioanalyzer or by agarose gel electrophoresis prior to RNA-Seq or other applications, respectively. PCR amplification of a 196-nucleotide region of 16S rDNA was used to confirm the absence of DNA (lack of any product, relative to a chromosomal DNA control) prior to RNA-Seq library creation.

### Library preparation and RNA sequencing

For each species, RNA samples from each of three timepoints were divided in two, with one half being subjected to rRNA depletion using the MICROBExpress^TM^ Bacterial mRNA Enrichment Kit (Ambion) as per the manufacturer’s instructions. Each of the rRNA depleted samples was then combined to generate a species-specific ‘pool’, such that each pool contained equal amounts of RNA from each time-point (vegetative growth, aerial growth/mycelial fragmentation, sporulation). Equivalent pools were created for the untreated (total) RNA samples. The two pools were destined to become two separate libraries: the rRNA-depleted pools were sequenced using a protocol optimized for full length transcripts, while the total RNA samples were enriched for sRNAs prior to sequencing.

All samples were treated with tobacco acid pyrophosphatase to create 5′ ends amenable for adapter ligation. To enrich for small RNAs, the total RNA samples were size selected (40 to 300 nucleotides) following polyacrylamide gel electrophoresis. RNA from both libraries was then fragmented in a buffered zinc solution and single stranded RNA adapters were ligated to the 5′ and 3′ ends to maintain strand specificity, prior to re-purifying on a polyacrylamide gel. Each pool was then reverse transcribed and PCR amplified to generate DNA colonies, which were sequenced using an Illumina HiSeq 2000 sequencer. For the sRNA-enriched library, read lengths ranged from 24 to 94 nucleotides, while read lengths were as long as 150 nucleotides for the long transcript-enriched library.

### Alignment of reads to genomes

Sequencing reads having low quality 3′ ends were trimmed using the program PrinSeq [84]. The quality trimmed reads were aligned to their respective genome sequence using Bowtie2 [85] and then sorted, indexed and converted to BAM format using SAMtools (Version 0.1.18) [86]. For the full length transcript libraries, 59,073,931 (~99.4% of total reads obtained during sequencing), 44,462,362 (~99.4%) and 94,358,187 reads (~99.4%) were mapped to the *S. coelicolor*, *S. avermitilis*, and *S. venezuelae* genomes respectively. For the sRNA-enriched libraries a total of 21,871,239 (~99.5%), 23,608,152 (~98.5%) and 21,880,716 (~97.6%) reads were mapped to the same respective genomes. The BAM files were visualized using Integrated Genomics Viewer (Version 2.0) [87]. BEDTools [88] was used to calculate the positive and negative-strand read depth at each nucleotide, and a Perl script was used to exploit the BEDTools output in calculating the average sense and antisense coverage per nucleotide of each annotated gene.

### Non-coding RNA analysis

The genes with the highest levels of antisense coverage from the full length transcript libraries were chosen for analysis. This included genes with an antisense ‘mean expression value’ (MEV) (average read depth per nucleotide) greater than 10.0 in *S. coelicolor*, 3.5 in *S. avermitilis*, and 4.1 in *S. venezuelae*; the different values were determined by normalizing the MEV to the number of non-ribosomal RNA sequences obtained for each species. Given that many known antisense RNAs overlap only a small fraction of their sense counterpart, we also used BEDTools to determine the highest peak antisense expression levels for each gene. We focused on those genes having a maximum expression level greater than 20.0 in *S. coelicolor*, 6.9 in *S. avermitilis*, and 8.2 in *S. venezuelae*. Again, cutoff values were chosen to reflect differences in numbers of non-ribosomal RNA sequences obtained for each species. Intergenic sRNAs were annotated manually using Integrated Genomics Viewer [87].

Homologous sRNAs were identified using BLASTN (E-value less than 1e-06) and aligned using Clustal Omega [89,90]. Selected alignments were computationally folded using CMFinder [91] on the WAR webserver [92]. Compensatory mutations that maintained secondary structure were located manually and the VARNA software package [93] was used to illustrate RNA secondary structure. RNA secondary structure predictions were performed using the program Mfold [80].

To evaluate the protein-coding potential of sRNAs, we focused on those sRNAs conserved in at least two of the three *Streptomyces* species identified in this study. Frame Plot 2.3.2 was used to highlight potential open-reading frames, and amino acid sequences of similar lengths (≤10% difference) were aligned using Clustal Omega [89,90]. Amino acid sequences with high similarity (≥65%) were deemed to have the potential to encode a conserved small peptide.

### Analysis of stable degradation products

A custom Perl script was used to identify mRNAs with defined regions of high coverage compared with the average coverage for the entire gene in the short-read library. The top hits from this analysis were visualized using Integrated Genomics Viewer, and putative stable regions that were grossly overrepresented (more than 100-fold) compared with the rest of the mRNA were identified. These stable regions were classified according to their location: 5′ end-associated; 3′ end-associated (or between genes in an operon); or internal to the mRNA coding sequence. A custom Perl script was also developed to analyze the nucleotide content for entire genes, stable regions, and sequences flanking the stable regions.

### Northern blotting

Northern blotting was performed as described previously [17,94], only RNA was crosslinked to membranes using a 1-ethyl-3-(3-dimethylaminopropyl) carbodiimide crosslinking solution at 55°C for 2 hours [95]. Membranes were stripped with high-stringency buffer (0.2% saline-sodium citrate, 0.1% sodium dodecyl sulfate) at 65°C. They were subsequently checked by exposure to a storage phosphor screen to ensure complete removal of all radiolabelled probe, and re-probed as necessary.

### Semi-quantitative RT-PCR

Semi-quantitative RT-PCR was conducted using total RNA isolated from *S. coelicolor* at three distinct life-cycle stages. For each reverse transcription reaction, 3 μg of RNA was mixed with 10 nmol of each dNTP, and 2 pmol of each gene-specific primer (Additional file 1: Table S6). RNase-free water was added to give a total reaction volume of 12 μL. Following mixing, each sample was first incubated at 65°C for 10 minutes and then immediately chilled on ice for 5 minutes. Reverse transcription was performed using SuperScript® III reverse transcriptase (Invitrogen) according to the manufactures instructions with a few modifications. Briefly, 4 μL of 5× First Strand Buffer, 2 μL of 0.1 M DTT and 1 μL of RNaseOUT™ were added to each reaction. After incubation at 42°C for 2 minutes, 1 μL of SuperScript® III reverse transcriptase was added. Reverse transcription was performed at 42°C for 60 minutes and reactions were terminated by incubating at 70°C for 15 minutes.

The reverse transcription products (2 μL) were then used as template for PCR amplification. A standard PCR protocol using *Taq* DNA polymerase (Norgen) was used, with primers indicated in (Additional file 1: Table S6). Annealing temperatures were optimized for each primer combination, as were the number of amplifications cycles (to ensure that amplification remained within the linear range). PCR products were separated on 2-3% agarose gels. Negative controls containing nuclease free water in lieu of reverse transcriptase were included to ensure there was no residual genomic DNA present in the RNA samples. Primers targeting 16S rRNA were used as positive controls for RNA quality. All reverse transcription/PCR reactions were carried out in triplicate, using RNA isolated from three independent RNA time-courses.

## Abbreviations

asRNA: Antisense RNA; bp: Base pair; cutoRNA: Convergent untranslated overlapping RNA; MEV: Mean expression value; MYM: Maltose-yeast extract-malt extract; ncRNA: Non-coding RNA; RNA-Seq: RNA sequencing; RT-PCR: Reverse transcription PCR; sRNA: Small RNA; UTR: Untranslated region

## Competing interests

The authors declare that they have no competing interests.

## Authors’ contributions

MJM contributed to the experimental design, isolated RNA, analyzed the RNA-Seq data, performed northern blots and drafted the manuscript. RAY performed the RT-PCR and helped to draft the manuscript. SEJ analyzed the RNA-Seq data and provided feedback on the manuscript. MAE conceived of the study, contributed to the experimental design and coordination, and drafted the manuscript. All authors have read and approved the final manuscript.

## Supplementary Material

Additional file 1: Tables S1-S6 and Figures S1-S2. The data sets supporting the results of this article are available in NCBI’s Gene Expression Omnibus and are accessible through GEO Series accession number GSE46008 (http://www.ncbi.nlm.nih.gov/geo/query/acc.cgi?acc=GSE46008). Click here for file
