# Supplementary material for: Comparative analysis of non-coding RNAs in the antibiotic-producing Streptomyces bacteria
Source: BMC Genomics. 2013 Aug 16;14:558. doi: 10.1186/1471-2164-14-558 (PMC3765725; doi:10.1186/1471-2164-14-558)
Supplement: Additional file 1: Tables S1-S6 and Figures S1-S2. — The data sets supporting the results of this article are available in NCBI’s Gene Expression Omnibus and are accessible through GEO Series accession number GSE46008 (http://www.ncbi.nlm.nih.gov/geo/query/acc.cgi?acc=GSE46008). [file 1471-2164-14-558-S1.pdf]

Table S1: Classification of antisense RNAs in *S. coelicolor*, *S. avermitilis* and *S. venezuelae*

Sense gene[abcd]	cis-antisense RNA	cutoRNA	Divergent transcription	Unclassified*	Biosynthetic clusters[x]
sco0058	-	-	-	Y	-
sco0102	Y	-	-	-	-
sco0103	Y	-	-	-	-
sco0166	-	Y	-	-	-
sco0195	Y	-	-	-	-
sco0224	-	Y	-	-	-
sco0297	Y	-	-	-	-
sco0298	Y	-	-	-	-
sco0324	-	Y	-	-	-
sco0402	-	Y	-	-	-
sco0412	-	Y	-	-	-
sco0460	-	Y	-	-	-
sco0477	-	Y	-	-	-
sco0490	-	Y	-	-	Coelichelin
sco0491	-	Y	-	-	Coelichelin
sco0500[d]	-	-	-	Y	-
sco0562	-	-	-	Y	-
sco0591	-	-	-	Y	-
sco0592	-	Y	-	-	-
sco0681	-	Y	-	-	-
sco0682	-	Y	-	-	-
sco0695	-	-	-	Y	-
sco0729	-	-	-	Y	-
sco0732	-	Y	-	-	-
sco0733	-	-	-	Y	-
sco0735	-	Y	-	-	-
sco0742	-	Y	-	-	-
sco0763	-	Y	-	-	-
sco0798	-	Y	-	-	-
sco0884	-	Y	-	-	-
sco0908	-	Y	-	-	-
sco0909	-	Y	-	-	-
sco0927	-	Y	-	-	-
sco0954	Y	-	-	-	-
sco0973	-	Y	-	-	-
sco0974	-	Y	-	-	-
sco0975	-	Y	-	-	-
sco0976	-	Y	-	-	-
sco0988	-	-	-	Y	-
sco0989	-	Y	-	-	-

sco0998	-	-	-	Y	-
sco1019	-	Y	-	-	-
sco1025	-	-	-	Y	-
sco1039	-	Y	-	-	-
sco1069	-	-	-	Y	-
sco1071	Y	-	-	-	-
sco1075	-	Y	-	-	-
sco1086	-	Y	-	-	-
sco1104	-	Y	-	-	-
sco1117	-	-	-	Y	-
sco1140	-	-	-	Y	-
sco1142	-	-	-	Y	-
sco1150[d]	-	Y	-	-	-
sco1153	-	Y	-	-	-
sco1154	-	-	-	Y	-
sco1157	-	Y	-	-	-
sco1165	-	Y	-	-	-
sco1172	Y	-	-	-	-
sco1193	-	Y	-	-	-
sco1211	-	-	-	Y	-
sco1224	-	-	-	Y	-
sco1240	Y	-	-	-	-
sco1242	-	-	-	Y	-
sco1243	-	-	-	-	-
sco1248	-	Y	-	-	-
sco1261	-	Y	-	-	-
sco1293	-	-	-	Y	-
sco1294	-	Y	-	-	-
sco1297	Y	-	-	-	-
sco1315	-	Y	-	-	-
sco1320	-	Y	-	-	-
sco1334	-	-	-	Y	-
sco1335	-	-	-	Y	-
sco1347	-	-	-	-	-
sco1358	-	Y	-	-	-
sco1394	-	-	-	Y	-
sco1404	-	Y	-	-	-
sco1427	-	Y	-	-	-
sco1428	-	Y	-	-	-
sco1451	-	Y	-	-	-
sco1489	-	-	-	Y	-
sco1504	-	-	-	Y	-

sco1530	Y	-	-	-	-
sco1535	Y	-	-	-	-
sco1542	-	Y	-	-	-
sco1544	-	Y	-	-	-
sco1545	-	Y	-	-	-
sco1552	-	-	-	Y	-
sco1562	-	Y	-	-	-
sco1588	-	-	-	Y	-
sco1589	-	Y	-	-	-
sco1590	-	-	-	Y	-
sco1602	-	-	-	Y	-
sco1610	-	Y	-	-	-
sco1622	Y	-	-	-	-
sco1624	Y	-	-	-	-
sco1625	-	Y	-	-	-
sco1650	-	Y	-	-	-
sco1651	Y	-	-	-	-
sco1669	Y	-	-	-	-
sco1671	-	Y	-	-	-
sco1673	Y	-	-	-	-
sco1674	-	Y	-	-	-
sco1690	-	-	-	Y	-
sco1698	-	Y	-	-	-
sco1730	-	Y	-	-	-
sco1750	-	Y	-	-	-
sco1753	-	Y	-	-	-
sco1757	Y	-	-	-	-
sco1764	-	-	-	Y	-
sco1784	-	Y	-	-	-
sco1795	-	-	Y	-	-
sco1796	-	-	-	Y	-
sco1800	-	-	-	Y	-
sco1808	-	Y	-	-	-
sco1809	-	Y	-	-	-
sco1813	-	-	-	Y	-
sco1821	-	-	-	-	-
sco1831	-	Y	-	-	-
sco1832	-	Y	-	-	-
sco1837	-	Y	-	-	-
sco1841	-	Y	-	-	-
sco1842	-	Y	-	-	-
sco1859	Y	-	-	-	-

sco1903	-	Y	-	-	-
sco1904	-	Y	-	-	-
sco1908	-	Y	-	-	-
sco1909	-	Y	-	-	-
sco1929	Y	-	-	-	-
sco1931	-	Y	-	-	-
sco1961	-	-	-	Y	-
sco1966	-	-	-	Y	-
sco1975	-	Y	-	-	-
sco1977	-	-	-	Y	-
sco1981	-	Y	-	-	-
sco1988	-	-	-	Y	-
sco1992	-	Y	-	-	-
sco2000	-	-	-	Y	-
sco2009	-	-	-	Y	-
sco2012	-	-	-	Y	-
sco2022	-	Y	-	-	-
sco2027	-	-	-	Y	-
sco2068	-	Y	-	-	-
sco2077	-	-	-	Y	-
sco2100	-	-	-	Y	-
sco2101	-	Y	-	-	-
sco2113	-	Y	-	-	-
sco2114	-	Y	-	-	-
sco2124	-	Y	-	-	-
sco2125	-	Y	-	-	-
sco2153	Y	-	-	-	-
sco2157	Y	-	-	-	-
sco2169	-	-	-	Y	-
sco2171	-	Y	-	-	-
sco2190	-	-	-	Y	-
sco2204	-	Y	-	-	-
sco2219	-	-	-	Y	-
sco2224	-	-	-	Y	-
sco2234[a]	Y	-	-	-	-
sco2237	-	Y	-	-	-
sco2238	-	Y	-	-	-
sco2244	-	-	-	Y	-
sco2251	-	Y	-	-	-
sco2259	-	Y	-	-	-
sco2260	-	Y	-	-	-
sco2268	-	Y	-	-	-

sco2269	-	-	-	Y	-
sco2274	-	-	-	Y	-
sco2279	-	Y	-	-	-
sco2293	-	-	-	Y	-
sco2299	-	Y	-	-	-
sco2311	Y	-	-	-	-
sco2320	-	-	-	Y	-
sco2331	-	Y	-	-	-
sco2338	-	Y	-	-	-
sco2346	-	-	-	Y	-
sco2351	-	Y	-	-	-
sco2357	-	Y	-	-	-
sco2358	-	Y	-	-	-
sco2364	Y	Y	-	-	-
sco2372	-	Y	-	-	-
sco2376	-	-	-	-	-
sco2383	Y	-	-	-	-
sco2384	-	Y	-	-	-
sco2385	-	-	-	Y	-
sco2438	-	Y	-	-	-
sco2439	-	Y	-	-	-
sco2465	-	-	-	Y	-
sco2484	-	-	-	Y	-
sco2497	-	-	-	Y	-
sco2509	-	Y	-	-	-
sco2526	-	Y	-	-	-
sco2527	-	Y	-	-	-
sco2540	-	-	-	-	-
sco2557	-	Y	-	-	-
sco2581	-	-	Y	-	-
sco2583	-	Y	-	-	-
sco2595	Y	-	-	-	-
sco2618	Y	-	-	-	-
sco2626	-	Y	-	-	-
sco2634	-	-	-	Y	-
sco2654	-	Y	-	-	-
sco2670	-	Y	-	-	-
sco2674	-	Y	-	-	-
sco2685	Y	-	-	-	-
sco2726	-	-	-	Y	-
sco2772	-	Y	-	-	-
sco2786	-	Y	-	-	-

sco2789	-	Y	-	-	-
sco2790	-	Y	-	-	-
sco2806	Y	-	-	-	-
sco2810	Y	-	-	-	-
sco2823	Y	-	-	-	-
sco2838	-	Y	-	-	-
sco2884	-	Y	-	-	-
sco2885	-	Y	-	-	-
sco2888	Y	-	-	-	-
sco2893	-	Y	-	-	-
sco2897	-	-	-	Y	-
sco2902	-	Y	-	-	-
sco2906	-	-	-	Y	-
sco2910	-	-	-	Y	-
sco2917	-	-	-	Y	-
sco2918	-	Y	-	-	-
sco2919	-	Y	-	-	-
sco2921	-	Y	-	-	-
sco2922	-	Y	-	-	-
sco2925	-	Y	-	-	-
sco2926	-	Y	-	-	-
sco2938	-	-	-	Y	-
sco2943	-	-	-	Y	-
sco2949	-	-	-	Y	-
sco2975	-	Y	-	-	-
sco2985	-	Y	-	-	-
sco2989	-	Y	-	-	-
sco3007[c]	-	Y	-	-	-
sco3015	Y	-	-	-	-
sco3017	-	-	-	Y	-
sco3023	-	-	-	Y	-
sco3024	-	-	-	Y	-
sco3039	-	-	-	Y	-
sco3043	-	-	-	Y	-
sco3047	-	Y	-	-	-
sco3049	-	Y	-	-	-
sco3050	-	Y	-	-	-
sco3052	-	-	-	Y	-
sco3053	-	Y	-	-	-
sco3057	-	-	-	Y	-
sco3076	-	Y	-	-	-
sco3080	-	Y	-	-	-

sco3087	-	-	-	Y	-
sco3106	-	-	-	Y	-
sco3107	-	Y	-	-	-
sco3118	-	-	-	Y	-
sco3133	-	Y	-	-	-
sco3158	-	Y	-	-	-
sco3173	-	-	-	Y	-
sco3175	Y	-	-	-	-
sco3176	-	-	-	Y	-
sco3191	-	Y	-	-	-
sco3195	-	Y	-	-	-
sco3196	-	Y	-	-	-
sco3202	Y	-	-	-	-
sco3203	-	-	-	Y	-
sco3227	-	Y	-	-	Calcium dependent antibiotic
sco3275	-	-	-	Y	-
sco3283	-	-	-	Y	-
sco3287[ab]	-	-	-	Y	-
sco3289	-	-	-	Y	-
sco3290	Y	-	-	-	-
sco3291	Y	-	-	-	-
sco3298	-	Y	-	-	-
sco3310	-	-	-	Y	-
sco3317[a]	Y	-	-	-	-
sco3318	Y	-	-	-	-
sco3320	Y	-	-	-	-
sco3321[a]	-	-	-	Y	-
sco3324	-	Y	-	-	-
sco3325	-	Y	-	-	-
sco3344	-	Y	-	-	-
sco3349	-	-	-	-	-
sco3350	-	-	-	Y	-
sco3352	-	Y	-	-	-
sco3360	-	Y	-	-	-
sco3364	-	Y	-	-	-
sco3372	-	-	-	Y	-
sco3374	-	Y	-	-	-
sco3394	-	Y	-	-	-
sco3402	-	Y	-	-	-
sco3403	-	Y	-	-	-
sco3408	Y	-	-	-	-

sco3410	-	-	-	Y	-
sco3413	-	Y	-	-	-
sco3423	-	Y	-	-	-
sco3424	-	Y	-	-	-
sco3432	-	Y	-	-	-
sco3433	Y	-	-	-	-
sco3481	Y	-	-	-	-
sco3482	Y	-	-	-	-
sco3483	Y	-	-	-	-
sco3487	Y	-	-	-	-
sco3497	-	-	-	Y	-
sco3503	-	-	-	Y	-
sco3505	-	-	-	Y	-
sco3535	-	-	-	Y	-
sco3536	-	Y	-	-	-
sco3550	-	-	Y	-	-
sco3559	-	-	-	Y	-
sco3560	Y	-	-	-	-
sco3567	-	-	-	Y	-
sco3572	-	Y	-	-	-
sco3578	-	Y	-	-	-
sco3579	-	-	-	Y	-
sco3602	-	-	-	-	-
sco3606	-	Y	-	-	-
sco3620	Y	-	-	-	-
sco3621	-	-	-	Y	-
sco3624	-	Y	-	-	-
sco3636	-	-	-	Y	-
sco3638	-	-	-	Y	-
sco3665	-	-	-	Y	-
sco3671	Y	-	-	-	-
sco3693	-	Y	-	-	-
sco3712	-	Y	-	-	-
sco3713	-	Y	-	-	-
sco3734	-	Y	-	-	-
sco3769	-	Y	-	-	-
sco3793	-	Y	-	-	-
sco3794	-	Y	-	-	-
sco3825	-	Y	-	-	-
sco3832	-	Y	-	-	-
sco3836	-	Y	-	-	-
sco3855	-	Y	-	-	-

sco3902	-	Y	-	-	-
sco3910	-	-	-	Y	-
sco3920	-	-	-	Y	-
sco3927	-	Y	-	-	-
sco3939	-	Y	-	-	-
sco3941	Y	-	-	-	-
sco3942	-	-	-	Y	-
sco3949	-	Y	-	-	-
sco3960	-	-	-	Y	-
sco3973	-	-	-	Y	-
sco3976	-	-	-	-	-
sco3989	Y	-	-	-	-
sco3991	-	-	-	Y	-
sco4008	-	-	-	Y	-
sco4014	-	Y	-	-	-
sco4025	-	Y	-	-	-
sco4034	-	-	-	Y	-
sco4038	Y	-	-	-	-
sco4041	-	-	-	Y	-
sco4042	-	Y	-	-	-
sco4056	-	Y	-	-	-
sco4057	-	-	-	Y	-
sco4079	-	-	-	Y	-
sco4080	-	Y	-	-	-
sco4087	-	Y	-	-	-
sco4088	-	Y	-	-	-
sco4090	-	-	-	Y	-
sco4118	Y	-	-	-	-
sco4134	-	Y	-	-	-
sco4135	-	Y	-	-	-
sco4136	-	-	-	Y	-
sco4137	Y	-	-	-	-
sco4164	Y	-	-	-	-
sco4172	-	Y	-	-	-
sco4182	-	Y	-	-	-
sco4183	-	-	-	Y	-
sco4186	-	Y	-	-	-
sco4188	-	-	-	Y	-
sco4201	Y	-	-	-	-
sco4206	-	-	-	Y	-
sco4223	-	-	-	Y	-
sco4232	-	-	-	Y	-

sco4259	-	-	-	Y	-
sco4261[a]	Y	-	-	-	-
sco4262	Y	-	-	-	-
sco4264	-	-	-	Y	-
sco4269	-	-	-	Y	-
sco4279	-	Y	-	-	-
sco4283[d]	-	Y	-	-	-
sco4290	-	Y	-	-	-
sco4298	-	Y	-	-	-
sco4299	-	Y	-	-	-
sco4310	-	Y	-	-	-
sco4316	-	-	-	Y	-
sco4317	-	-	-	Y	-
sco4318	-	Y	-	-	-
sco4323	-	Y	-	-	-
sco4324	-	-	-	Y	-
sco4325	-	Y	-	-	-
sco4326	-	Y	-	-	-
sco4334	-	Y	-	-	-
sco4358	-	-	-	Y	-
sco4365	-	Y	-	-	-
sco4371	Y	-	-	-	-
sco4388	-	-	-	Y	-
sco4401	-	Y	-	-	-
sco4407	-	Y	-	-	-
sco4413	-	-	-	Y	-
sco4424	-	Y	-	-	-
sco4425[c]	-	Y	-	-	-
sco4426	-	-	-	Y	-
sco4430	-	Y	-	-	-
sco4431	-	Y	-	-	-
sco4440	Y	-	-	-	-
sco4443	-	Y	-	-	-
sco4447	-	-	-	Y	-
sco4474	-	-	-	Y	-
sco4488	-	Y	-	-	-
sco4489	-	-	-	Y	-
sco4511	-	Y	-	-	-
sco4524	Y	-	-	-	-
sco4536	Y	-	-	-	-
sco4537	Y	-	-	-	-
sco4557	-	Y	-	-	-

sco4560	-	-	-	Y	-
sco4564	Y	-	-	-	-
sco4565	Y	-	-	-	-
sco4566[a]	Y	-	-	-	-
sco4567[a]	Y	-	-	-	-
sco4579	-	-	-	Y	-
sco4588	-	Y	-	-	-
sco4589	-	Y	-	-	-
sco4591	-	Y	-	-	-
sco4602	Y	-	-	-	-
sco4603	Y	-	-	-	-
sco4604	Y	-	-	-	-
sco4605	Y	-	-	-	-
sco4606	Y	-	-	-	-
sco4607	Y	-	-	-	-
sco4613	-	Y	-	-	-
sco4614	-	Y	-	-	-
sco4632	-	-	-	Y	-
sco4657	-	-	-	Y	-
sco4662	-	-	-	Y	-
sco4680	-	Y	-	-	-
sco4692[a]	-	-	-	Y	-
sco4693[a]	Y	-	-	-	-
sco4699[a]	-	-	-	Y	-
sco4702	-	-	-	Y	-
sco4721	-	-	-	Y	-
sco4731	-	Y	-	-	-
sco4737	-	-	-	Y	-
sco4749[d]	-	Y	-	-	-
sco4761	Y	-	-	-	-
sco4772	-	-	-	-	-
sco4773	-	Y	-	-	-
sco4789	-	Y	-	-	-
sco4792	-	Y	-	-	-
sco4793	-	Y	-	-	-
sco4794	-	-	-	Y	-
sco4795	-	-	-	Y	-
sco4801	-	Y	-	-	-
sco4811	-	-	-	Y	-
sco4825	Y	-	-	-	-
sco4829	Y	-	-	-	-
sco4840	-	Y	-	-	-

sco4841	-	Y	-	-	-
sco4846	-	Y	-	-	-
sco4848	Y	-	-	-	-
sco4872	-	-	-	Y	-
sco4909	-	-	-	Y	-
sco4912	-	Y	-	-	-
sco4921	-	-	-	Y	-
sco4928	-	Y	-	-	-
sco4929	-	Y	-	-	-
sco4933	-	-	-	Y	-
sco4953	-	Y	-	-	-
sco4957	-	-	-	Y	-
sco4960	-	-	-	Y	-
sco4965	Y	-	-	-	-
sco4969	-	-	-	Y	-
sco4975	-	-	-	Y	-
sco4995	-	Y	-	-	-
sco5049	-	Y	-	-	-
sco5054	-	Y	-	-	-
sco5060	-	Y	-	-	-
sco5062	-	-	-	Y	-
sco5063	-	-	-	Y	-
sco5073	Y	-	-	-	Actinorhodin
sco5080	Y	-	-	-	Actinorhodin
sco5081	-	Y	-	-	Actinorhodin
sco5082	-	Y	-	-	Actinorhodin
sco5099	Y	-	-	-	-
sco5106[d]	-	Y	-	-	-
sco5121	Y	-	-	Y	-
sco5123	-	Y	-	-	-
sco5124	-	Y	-	-	-
sco5125	-	Y	-	-	-
sco5134	-	-	-	Y	-
sco5138	-	-	-	-	-
sco5143	-	-	-	Y	-
sco5146[d]	-	Y	-	-	-
sco5151	-	Y	-	-	-
sco5152	-	Y	-	-	-
sco5155	-	Y	-	-	-
sco5164	-	Y	-	-	-
sco5167	-	Y	-	-	-
sco5168	-	Y	-	-	-

sco5177	-	-	-	Y	-
sco5178	-	Y	-	-	-
sco5182	-	-	-	Y	-
sco5183	-	-	-	Y	-
sco5197	-	Y	-	-	-
sco5198	-	Y	-	-	-
sco5200	-	-	Y	-	-
sco5202	-	Y	-	-	-
sco5203	-	-	Y	Y	-
sco5209	-	-	-	Y	-
sco5223	-	Y	-	-	Albaflavenone
sco5224	-	-	-	Y	-
sco5241	Y	-	-	-	-
sco5242	-	Y	-	-	-
sco5243	-	-	-	Y	-
sco5255	-	Y	-	-	-
sco5256	-	Y	-	-	-
sco5294	-	Y	-	-	-
sco5329	Y	-	-	-	-
sco5330	-	Y	-	-	-
sco5333	-	Y	-	-	-
sco5334	-	-	-	Y	-
sco5356	-	-	-	Y	-
sco5380	-	-	-	Y	-
sco5389	-	-	-	Y	-
sco5390	-	Y	-	-	-
sco5392	-	-	-	Y	-
sco5398	Y	-	-	-	-
sco5410	-	-	-	Y	-
sco5411	-	-	-	Y	-
sco5418	-	-	-	Y	-
sco5422	-	-	-	Y	-
sco5427	-	Y	-	-	-
sco5445	-	-	-	Y	-
sco5465	-	Y	-	-	-
sco5466	-	Y	-	-	-
sco5488	-	Y	-	-	-
sco5489	-	Y	-	-	-
sco5506	-	-	-	Y	-
sco5507	-	Y	-	-	-
sco5516	-	-	-	Y	-
sco5518	-	-	-	Y	-

sco5543	Y	-	-	-	-
sco5545	Y	-	-	-	-
sco5558	Y	-	-	-	-
sco5561	-	Y	-	-	-
sco5573	-	Y	-	-	-
sco5576	-	-	-	Y	-
sco5581	-	Y	-	-	-
sco5630	-	-	-	Y	-
sco5641	-	-	-	Y	-
sco5650	-	-	-	Y	-
sco5661	Y	-	-	-	-
sco5664	-	Y	-	-	-
sco5706	-	-	-	Y	-
sco5709	-	-	-	Y	-
sco5722	-	Y	-	-	-
sco5727	-	Y	-	-	-
sco5760	-	Y	-	-	-
sco5781	-	Y	-	-	-
sco5782	-	-	-	Y	-
sco5788	-	-	-	Y	-
sco5802	-	-	-	Y	-
sco5810	-	-	-	Y	-
sco5814	-	Y	-	-	-
sco5821	-	-	-	Y	-
sco5828	-	Y	-	-	-
sco5835	-	Y	-	-	-
sco5841	-	-	-	Y	-
sco5842	Y	-	-	-	-
sco5856	-	Y	-	-	-
sco5866	-	Y	-	-	-
sco5897	Y	-	-	-	Prodiginine
sco5986	-	Y	-	-	-
sco5988	-	Y	-	-	-
sco5989	-	-	-	Y	-
sco6002	-	Y	-	-	-
sco6003	-	-	-	Y	-
sco6029	Y	-	-	-	-
sco6039	-	-	-	Y	-
sco6042	-	Y	-	-	-
sco6043	-	Y	-	-	-
sco6066	-	-	-	Y	-
sco6077	-	Y	-	-	-

sco6085	-	Y	-	-	-
sco6093	-	-	-	Y	-
sco6106	-	-	-	Y	-
sco6127	-	Y	-	-	-
sco6156	-	-	-	Y	-
sco6196	-	Y	-	-	-
sco6222	-	Y	-	-	-
sco6246	-	-	-	-	-
sco6265	-	-	-	Y	-
sco6266	Y	-	-	-	SCB1
sco6268	-	Y	-	-	-
sco6271	-	-	-	Y	-
sco6273	-	-	-	-	yCPK
sco6274	-	-	-	Y	yCPK
sco6277[d]	Y	-	-	-	yCPK
sco6281	Y	-	-	-	yCPK
sco6284	-	-	-	Y	yCPK
sco6285	-	-	-	Y	yCPK
sco6311	-	-	-	Y	-
sco6312	-	-	-	Y	-
sco6373	Y	-	-	-	-
sco6375	Y	-	-	-	-
sco6377	-	-	-	Y	-
sco6390	-	-	-	Y	-
sco6393	Y	-	-	-	-
sco6394	Y	-	-	-	-
sco6396	-	-	-	Y	-
sco6422	-	Y	-	-	-
sco6438	-	Y	-	-	Dipeptide
sco6455	-	Y	-	-	-
sco6477	-	-	-	Y	-
sco6492	-	Y	-	-	-
sco6598	Y	-	-	-	-
sco6624[c]	Y	-	-	-	-
sco6636	-	Y	-	-	-
sco6646	-	Y	-	-	-
sco6654	-	-	-	Y	-
sco6704	-	-	-	Y	-
sco6715	Y	-	-	-	-
sco6716[d]	-	Y	-	-	-
sco6717	-	Y	-	-	-
sco6721[a]	-	-	-	Y	-

sco6723	-	-	-	Y	-
sco6725	-	Y	-	-	-
sco6729[d]	-	Y	-	-	-
sco6733	-	-	-	Y	-
sco6735	-	Y	-	-	-
sco6736	-	-	-	-	-
sco6740	-	Y	-	-	-
sco6742	-	Y	-	-	-
sco6749	-	Y	-	-	-
sco6760	-	-	-	Y	Hopene
sco6761	Y	-	-	-	Hopene
sco6762	Y	-	-	-	Hopene
sco6770	-	-	-	Y	Hopene
sco6771	-	Y	-	-	
sco6772	-	Y	-	-	-
sco6775	Y	-	-	-	-
sco6777	-	Y	-	-	-
sco6828	-	-	-	Y	-
sco6861	-	-	-	Y	-
sco6947	-	-	-	Y	-
sco6949	-	-	-	Y	-
sco6998	-	Y	-	-	-
sco7009	-	Y	-	-	-
sco7022	Y	-	-	-	-
sco7047	-	-	-	Y	-
sco7101	-	-	-	Y	-
sco7173	-	-	-	Y	-
sco7219	-	-	-	Y	-
sco7234	-	-	-	Y	-
sco7249	-	Y	-	-	-
sco7250	-	Y	-	-	-
sco7252	-	-	-	-	-
sco7253	-	Y	-	-	-
sco7258	-	Y	-	-	-
sco7268	-	Y	-	-	-
sco7300	-	Y	-	-	-
sco7343	-	Y	-	-	-
sco7455	-	-	-	Y	-
sco7456	-	-	-	Y	-
sco7480	-	Y	-	-	-
sco7603	-	-	-	Y	-
sco7605	-	Y	-	-	-

sco7652	-	Y	-	-	-
sco7653	-	-	-	Y	-
sco7716	-	Y	-	-	-
sco7717	Y	-	-	-	-
sco7718	-	-	-	Y	-
sco7757	-	-	-	Y	-
sco7835	-	-	-	Y	-
sav0047	-	-	-	Y	-
sav0112	-	-	-	Y	-
sav0164	-	-	-	Y	-
sav0193	-	-	-	Y	-
sav0200	-	-	-	Y	-
sav0220	-	-	-	Y	-
sav0234	-	-	-	Y	-
sav0243	-	-	-	Y	-
sav0244	-	-	-	Y	-
sav0247	-	-	-	Y	-
sav0259	-	-	-	Y	Microcin
sav0263	-	-	-	Y	-
sav0272	-	-	-	Y	-
sav0274	-	-	-	Y	-
sav0277	-	-	-	Y	-
sav0334	-	-	-	Y	-
sav0410	Y	Y	-	-	-
sav0435	-	-	-	Y	-
sav0514	-	-	-	Y	-
sav0541	-	-	-	Y	-
sav0573	-	-	-	Y	-
sav0675	-	-	-	Y	-
sav0749	-	-	-	Y	-
sav0779	-	-	-	Y	-
sav0783	-	-	-	Y	-
sav0784	-	-	-	Y	-
sav0824	-	-	-	Y	-
sav0825	-	-	-	Y	-
sav0886	-	-	-	Y	-
sav0896	-	-	-	Y	-
sav0897	-	-	-	Y	-
sav0935	-	Y	-	-	Avermectin
sav0936	Y	Y	-	-	Avermectin
sav0937	Y	-	-	-	Avermectin

sav0938	-	-	-	Y	Avermectin
sav0939	-	-	-	Y	Avermectin
sav0942	-	-	-	Y	Avermectin
sav0945	-	Y	-	-	Avermectin
sav0946	-	Y	-	-	Avermectin
sav0948	-	-	-	Y	Avermectin
sav0949	-	Y	-	-	Avermectin
sav0962	-	-	-	Y	-
sav0980	-	-	-	Y	-
sav1016	-	-	-	Y	-
sav1043	-	-	-	Y	-
sav1047	-	-	-	Y	-
sav1048	Y	Y	-	-	-
sav1060	-	-	-	Y	-
sav1083	-	-	-	Y	-
sav1090	Y	-	-	-	-
sav1092	-	Y	-	-	-
sav1100	-	-	-	Y	-
sav1104	-	-	-	Y	-
sav1111	-	-	-	Y	-
sav1118	-	-	-	Y	-
sav1138	-	Y	-	-	-
sav1161	-	-	-	Y	-
sav1162	-	Y	-	-	-
sav1164	-	-	-	Y	-
sav1187	-	-	-	Y	-
sav1235	-	Y	-	-	-
sav1236	-	Y	-	-	-
sav1237	-	-	-	Y	-
sav1238	-	Y	-	-	-
sav1247	-	-	-	Y	-
sav1300	-	-	-	Y	-
sav1308	-	-	-	Y	-
sav1332	-	-	-	Y	-
sav1410	-	-	-	Y	-
sav1423	-	-	-	Y	-
sav1463	-	-	-	Y	-
sav1486	-	Y	-	-	-
sav1502	-	-	-	Y	-
sav1526	-	-	-	Y	-
sav1572	-	-	-	Y	-
sav1574	-	-	-	Y	-

sav1578	-	-	-	Y	-
sav1589	-	Y	-	-	-
sav1635	-	-	-	Y	-
sav1642	-	Y	-	-	-
sav1644	-	-	-	Y	-
sav1652	Y	-	-	-	Hopene
sav1663	-	Y	-	-	-
sav1664	-	-	-	Y	-
sav1676	Y	-	-	-	-
sav1684	-	-	-	Y	-
sav1692	-	-	-	Y	-
sav1704	-	-	-	Y	-
sav1705	-	-	-	Y	-
sav1792	-	-	-	Y	-
sav1799	-	-	-	Y	-
sav1837	-	Y	-	-	-
sav1895	-	Y	-	-	-
sav1939	-	-	-	Y	-
sav1956	-	-	-	Y	-
sav1959	-	-	-	Y	-
sav2001	-	-	-	Y	-
sav2034	-	-	-	Y	-
sav2048	-	-	-	Y	-
sav2061	-	-	-	Y	-
sav2075	-	-	-	Y	-
sav2085	-	-	-	Y	-
sav2089	-	-	-	Y	-
sav2116	-	-	-	Y	-
sav2160	-	-	-	Y	-
sav2169	-	-	-	Y	-
sav2203	-	-	-	Y	-
sav2220	-	-	-	Y	-
sav2225	-	-	-	Y	-
sav2227	-	-	-	Y	-
sav2232	-	-	-	Y	-
sav2261	-	-	-	Y	-
sav2268	-	-	-	Y	2-alkyl-4-hydroxymethylfuran-3-carboxylic acids
sav2272	-	Y	-	-	-
sav2325	-	Y	-	-	-
sav2326	-	-	-	Y	-

sav2363	-	-	-	Y	-
sav2382	-	-	-	Y	Aromatic polyketide
sav2406	-	-	-	Y	-
sav2410	-	-	-	Y	-
sav2417	Y	-	-	-	-
sav2424	-	-	-	Y	-
sav2435	-	-	-	Y	-
sav2446	-	-	-	Y	-
sav2451	-	-	-	Y	-
sav2456	-	-	-	Y	-
sav2464	-	-	-	Y	-
sav2490	-	-	-	Y	-
sav2539	-	-	-	Y	-
sav2551	Y	-	-	-	-
sav2565	-	-	-	Y	-
sav2574	-	-	-	Y	-
sav2628	-	-	-	Y	-
sav2653	-	-	-	Y	-
sav2677	-	-	-	Y	-
sav2678	-	-	-	Y	-
sav2693	Y	-	-	-	-
sav2719	-	-	-	Y	-
sav2738	-	-	-	Y	-
sav2770	-	-	-	Y	-
sav2797	-	-	-	Y	-
sav2799	-	Y	-	-	-
sav2821	-	Y	-	-	-
sav2822	-	-	-	Y	-
sav2829	-	-	-	Y	-
sav2831	-	-	-	Y	-
sav2854	-	-	-	Y	-
sav2865	-	Y	-	-	-
sav2866	-	Y	-	-	-
sav2875	-	-	-	Y	-
sav2906	-	-	-	Y	-
sav2918	Y	-	-	-	-
sav2933	-	Y	-	-	-
sav2957	-	-	-	Y	-
sav2970	-	-	-	Y	-
sav2971	-	-	-	Y	-
sav2985	Y	-	-	-	-
sav2986	-	Y	-	-	-

sav2987	-	-	-	Y	-
sav2988	-	Y	-	-	-
sav3014	-	-	-	Y	-
sav3050	-	Y	-	-	-
sav3051	-	-	-	Y	-
sav3074	-	-	-	Y	-
sav3082	-	-	-	Y	-
sav3097	-	-	-	Y	-
sav3119	Y	-	-	-	-
sav3123	-	-	-	Y	-
sav3142	-	Y	-	-	-
sav3143	-	-	-	Y	-
sav3147	-	-	-	Y	-
sav3148	-	-	-	Y	-
sav3149	-	-	-	Y	-
sav3164	Y	-	-	-	Non-ribosomal peptide
sav3165	Y	-	-	-	-
sav3167	-	-	-	Y	-
sav3168	-	Y	-	-	-
sav3198	Y	-	-	-	Non-ribosomal peptide
sav3208	-	-	-	Y	-
sav3214	Y	-	-	-	-
sav3215	Y	-	-	-	-
sav3265	-	-	-	Y	-
sav3291	-	-	-	Y	-
sav3297	-	Y	-	-	-
sav3299	Y	-	-	-	-
sav3306	-	Y	-	-	-
sav3310	-	Y	-	-	-
sav3315	-	-	-	Y	-
sav3328	-	-	-	Y	-
sav3335	-	-	-	Y	-
sav3336	-	-	-	Y	-
sav3338	-	-	-	Y	-
sav3355	-	-	-	Y	-
sav3364	-	-	-	Y	-
sav3407	-	-	-	Y	-
sav3429	-	-	-	Y	-
sav3447	-	-	-	Y	-
sav3459	-	Y	-	-	-

sav3462	-	Y	-	-	-
sav3465	-	-	-	Y	-
sav3469	-	-	-	Y	-
sav3478	-	-	-	Y	-
sav3492	-	-	-	Y	-
sav3493	Y	-	-	-	-
sav3494	-	-	-	Y	-
sav3502	-	-	-	Y	-
sav3503	-	-	-	Y	-
sav3513	-	-	-	Y	-
sav3547	-	-	-	Y	-
sav3559	-	-	-	Y	-
sav3571	-	Y	-	-	-
sav3580	-	-	-	Y	-
sav3596	-	-	-	Y	-
sav3597	-	-	-	Y	-
sav3626	-	Y	-	-	-
sav3673	-	-	-	Y	-
sav3682	-	-	-	Y	-
sav3687	-	-	-	Y	-
sav3695	Y	-	-	-	-
sav3702	-	Y	-	-	-
sav3703	-	Y	-	-	-
sav3711	-	-	-	Y	-
sav3714	-	-	-	Y	-
sav3730	-	-	-	Y	-
sav3775	-	-	-	Y	-
sav3783	Y	-	-	-	-
sav3790	-	-	-	Y	-
sav3794	-	Y	-	-	-
sav3806	-	-	-	Y	-
sav3841	-	-	-	Y	-
sav3848	-	-	-	Y	-
sav3853	-	-	-	Y	-
sav3860	-	-	-	Y	-
sav3889	-	-	-	Y	-
sav3897	-	Y	-	-	-
sav3906	-	Y	-	-	-
sav3910	-	-	-	Y	-
sav3913	-	-	-	Y	-
sav3931	-	-	-	Y	-
sav3936	-	-	-	Y	-

sav3942	-	Y	-	-	-
sav3965	Y	-	-	-	-
sav3970	-	-	-	Y	-
sav3982	-	-	-	Y	-
sav3983	-	-	-	Y	-
sav4024	-	-	-	Y	-
sav4048	-	-	-	Y	-
sav4054	-	-	-	Y	-
sav4059	-	-	-	Y	-
sav4077	Y	-	-	-	-
sav4078	-	Y	-	-	-
sav4081	-	-	-	Y	-
sav4111	-	-	-	Y	-
sav4133	-	Y	-	-	-
sav4134	-	-	-	Y	-
sav4177	-	-	-	Y	-
sav4181	-	-	-	Y	-
sav4185	-	-	-	Y	-
sav4187	-	-	-	Y	-
sav4193	-	Y	-	-	-
sav4235	Y	-	-	-	-
sav4266	-	Y	-	-	-
sav4275	-	Y	-	-	-
sav4277	-	-	-	Y	-
sav4326	-	-	-	Y	-
sav4328	Y	-	-	-	-
sav4349	-	-	-	Y	-
sav4357	-	-	-	Y	-
sav4361	-	-	-	Y	-
sav4367	Y	-	-	-	-
sav4369	-	-	-	Y	-
sav4387	-	-	-	Y	-
sav4397	-	-	-	Y	-
sav4398	-	-	-	Y	-
sav4401	-	-	-	Y	-
sav4421	-	-	-	Y	-
sav4428	-	-	-	Y	-
sav4429	-	-	-	Y	-
sav4435	-	Y	-	-	-
sav4437	-	-	-	Y	-
sav4489	-	-	-	Y	-
sav4510	-	Y	-	-	-

sav4516	-	-	-	Y	-
sav4548	-	-	-	Y	-
sav4554	-	Y	-	-	-
sav4563	-	Y	-	-	-
sav4585	-	Y	-	-	-
sav4590	-	-	-	Y	-
sav4595	-	-	-	Y	-
sav4601	-	-	-	Y	-
sav4613	-	-	-	Y	-
sav4626	-	-	-	Y	-
sav4625	-	-	-	Y	-
sav4662	Y	-	-	-	-
sav4667	-	-	-	Y	-
sav4668	-	-	-	Y	-
sav4679	-	-	-	Y	-
sav4696	-	-	-	Y	-
sav4712	-	Y	-	-	-
sav4733	-	-	-	Y	-
sav4737	-	-	-	Y	-
sav4738	Y	-	-	-	-
sav4739	-	-	-	Y	-
sav4740	Y	-	-	-	-
sav4741	Y	-	-	-	-
sav4743	-	-	-	Y	-
sav4745	-	-	-	Y	-
sav4754	-	-	-	Y	-
sav4757	-	-	-	Y	-
sav4762	-	-	-	Y	-
sav4782	-	-	-	Y	-
sav4783	-	-	-	Y	-
sav4792	-	-	-	Y	-
sav4794	-	-	-	Y	-
sav4795	-	-	-	Y	-
sav4819	-	-	-	Y	-
sav4823	-	-	-	Y	-
sav4835	-	Y	-	-	-
sav4840	Y	-	-	-	-
sav4841	Y	-	-	-	-
sav4842	Y	-	-	-	-
sav4843	Y	-	-	-	-
sav4852	-	-	-	Y	-
sav4886	Y	-	-	-	-

sav4889	Y	-	-	-	-
sav4894	-	-	-	Y	-
sav4895	-	-	-	Y	-
sav4905	-	-	-	Y	-
sav4955	-	Y	-	-	-
sav4960	-	-	-	Y	-
sav4971	-	-	-	Y	-
sav5004	-	-	-	Y	-
sav5024	-	-	-	Y	-
sav5025	Y	-	-	-	-
sav5053	-	-	-	Y	-
sav5054	-	-	-	Y	-
sav5059	-	-	-	Y	-
sav5061	-	-	-	Y	-
sav5068	-	-	-	Y	-
sav5069	-	-	-	-	-
sav5085	-	-	-	Y	-
sav5117	-	-	-	Y	-
sav5164	-	Y	-	-	-
sav5180	-	-	-	Y	-
sav5188	-	Y	-	-	-
sav5189	-	-	-	Y	-
sav5190	-	-	-	Y	-
sav5231	-	-	-	Y	-
sav5240	Y	-	-	-	-
sav5245	-	-	-	Y	-
sav5246	-	-	-	Y	-
sav5268	-	Y	-	-	-
sav5293	-	-	-	Y	-
sav5304	-	-	-	Y	-
sav5305	-	-	-	Y	-
sav5363	Y	-	-	-	-
sav5369	-	Y	-	-	-
sav5371	-	-	-	Y	-
sav5379	-	-	-	Y	-
sav5407	Y	-	-	-	-
sav5412	-	-	-	Y	-
sav5439	-	-	-	Y	-
sav5444	Y	-	-	-	-
sav5475	-	-	-	Y	-
sav5483	-	-	-	Y	-
sav5485	-	-	-	Y	-

sav5489	-	-	-	Y	-
sav5494	-	Y	-	-	-
sav5525	-	-	-	Y	-
sav5548	-	Y	-	-	-
sav5587	-	-	-	Y	-
sav5592	-	-	-	Y	-
sav5596	-	-	-	Y	-
sav5624	-	-	-	Y	-
sav5638	-	Y	-	-	-
sav5650	-	Y	-	-	-
sav5679	-	-	-	Y	-
sav5690	-	-	-	Y	-
sav5695	-	-	-	Y	-
sav5699	-	Y	-	-	-
sav5700	-	Y	-	-	-
sav5701	-	-	-	Y	-
sav5708	-	-	-	Y	-
sav5709	-	-	-	Y	-
sav5711	-	Y	-	-	-
sav5741	Y	-	-	-	-
sav5756	-	-	-	Y	-
sav5764	-	-	-	Y	-
sav5780	-	-	-	Y	-
sav5781	-	-	-	Y	-
sav5782	-	-	-	Y	-
sav5794	-	Y	-	-	-
sav5807	Y	-	-	-	-
sav5810	-	-	-	Y	-
sav5823	-	-	-	Y	-
sav5836	-	Y	-	-	-
sav5837	-	Y	-	-	-
sav5862	-	-	-	Y	-
sav5872	-	-	-	Y	-
sav5882	-	-	-	Y	-
sav5913	-	-	-	Y	-
sav5929	-	Y	-	-	-
sav5933	-	Y	-	-	-
sav5936	-	-	-	Y	-
sav5937	-	-	-	Y	-
sav5985	-	-	-	Y	-
sav5988	-	-	-	Y	-
sav6012	-	-	-	Y	-

sav6015	-	Y	-	-	-
sav6034	-	-	-	Y	-
sav6048	-	-	-	Y	-
sav6050	-	-	-	Y	-
sav6057	-	-	-	Y	-
sav6059	-	-	-	Y	-
sav6061	Y	-	-	-	-
sav6088	-	-	-	Y	-
sav6090	-	-	-	Y	-
sav6130	-	-	-	Y	-
sav6134	-	-	-	Y	-
sav6138	-	-	-	Y	-
sav6139	-	-	-	Y	-
sav6171	Y	-	-	-	-
sav6228	-	-	-	Y	-
sav6233	Y	-	-	-	-
sav6237	-	-	-	Y	-
sav6241	-	Y	-	-	-
sav6256	-	-	-	Y	-
sav6261	-	-	-	Y	-
sav6287	-	-	-	Y	-
sav6303	-	-	-	Y	-
sav6320	-	-	-	Y	-
sav6391	-	-	-	Y	-
sav6399	Y	-	-	-	-
sav6410	-	-	-	Y	-
sav6423	-	Y	-	-	-
sav6429	-	-	-	Y	-
sav6434	-	-	-	Y	-
sav6456	-	-	-	Y	-
sav6464	-	-	-	Y	-
sav6483	-	Y	-	-	-
sav6498	-	-	-	Y	-
sav6533	-	-	-	Y	-
sav6542	-	Y	-	-	-
sav6543	-	Y	-	-	-
sav6556	-	-	-	Y	-
sav6561	-	-	-	Y	-
sav6567	-	-	-	Y	-
sav6585	-	-	-	Y	-
sav6597	-	-	-	Y	-
sav6602	-	-	-	Y	-

sav6606	-	-	-	Y	-
sav6608	-	-	-	Y	-
sav6636	-	-	-	Y	-
sav6637	Y	-	-	-	-
sav6648	-	-	-	Y	-
sav6674	Y	-	-	-	-
sav6675	-	Y	-	-	-
sav6696	-	-	-	Y	-
sav6707	-	-	-	Y	-
sav6711	-	-	-	Y	-
sav6712	-	-	-	Y	-
sav6731	-	-	-	Y	-
sav6787	-	-	-	Y	-
sav6805	-	Y	-	-	-
sav6816	-	-	-	Y	-
sav6823	-	Y	-	-	-
sav6888	-	-	-	Y	-
sav6893	-	Y	-	-	-
sav6894	-	Y	-	-	-
sav6897	-	-	-	Y	-
sav6956	-	Y	-	-	-
sav6959	-	-	-	Y	-
sav6961	-	-	-	Y	-
sav6991	-	-	-	Y	-
sav6993	-	-	-	Y	-
sav7004	-	-	-	Y	-
sav7039	-	-	-	Y	-
sav7058	-	-	-	Y	-
sav7059	-	-	-	Y	-
sav7075	-	-	-	Y	-
sav7087	-	-	-	Y	-
sav7088	-	Y	-	-	-
sav7090	-	-	-	Y	-
sav7094	-	Y	-	-	-
sav7096	-	-	-	Y	-
sav7098	-	Y	-	-	-
sav7118	-	-	-	Y	-
sav7129	-	-	-	Y	-
sav7165	-	-	-	Y	Non-ribosomal peptide
sav7188	-	-	-	Y	-
sav7218	-	-	-	Y	-

sav7247	-	-	-	Y	-
sav7248	-	Y	-	-	-
sav7249	-	-	-	Y	-
sav7250	-	Y	-	-	-
sav7251	-	Y	-	-	-
sav7289	-	-	-	Y	-
sav7299	-	-	-	Y	-
sav7311	-	-	-	Y	-
sav7313	-	-	-	Y	-
sav7327	-	-	-	Y	-
sav7337	-	-	-	Y	-
sav7343	-	-	-	Y	-
sav7346	-	Y	-	-	-
sav7388	-	Y	-	-	-
sav7389	-	Y	-	-	-
sav7419	-	-	-	Y	-
sav7429	-	-	-	Y	-
sav7442	Y	-	-	-	-
sav7448	-	-	-	Y	-
sav7449	Y	-	-	-	-
sav7477	-	-	-	Y	-
sav7481	-	-	-	Y	-
sav7486	-	-	-	Y	-
sav7487	-	Y	-	-	-
sav7498	-	-	-	Y	-
sav7513	-	-	-	Y	-
sav7519	-	-	-	Y	-
sav7536	-	-	-	Y	-
sav7544	-	-	-	Y	-
sven0002	-	-	-	Y	-
sven0003	-	-	-	Y	-
sven0013	-	-	-	Y	-
sven0053	-	-	-	Y	-
sven0066	-	-	-	Y	-
sven0181	-	-	-	Y	-
sven0203	-	-	-	Y	-
sven0204	-	-	-	Y	-
sven0216	-	-	-	Y	-
sven0227	-	-	-	Y	Ectoine
sven0253	-	-	-	Y	-
sven0265	-	Y	-	-	Terpene

sven0287	-	-	-	Y	-
sven0301	-	-	-	Y	-
sven0335	-	-	-	Y	-
sven0338	-	Y	-	-	-
sven0347	Y	-	-	-	-
sven0363	-	-	-	Y	-
sven0365	-	Y	-	-	-
sven0367	-	-	-	Y	-
sven0369	-	-	-	Y	-
sven0375	-	-	-	Y	-
sven0422	-	-	-	Y	-
sven0432	-	-	-	Y	-
sven0434	-	-	-	Y	-
sven0452	Y	-	-	-	-
sven0480	-	-	-	Y	Polyketide / non-ribosomal peptide hybrid
sven0501	-	-	-	Y	Polyketide / non-ribosomal peptide hybrid
sven0516	-	-	-	Y	Polyketide / non-ribosomal peptide hybrid
sven0519	-	-	-	Y	Polyketide / non-ribosomal peptide hybrid
sven0552	-	Y	-	-	Terpene
sven0579	-	-	-	Y	-
sven0591	-	Y	-	-	-
sven0604	-	-	-	Y	-
sven0605	Y	-	-	-	-
sven0611	-	-	-	Y	-
sven0645	-	-	-	Y	Lantipeptide
sven0658	-	-	-	Y	Lantipeptide
sven0659	Y	-	-	-	Lantipeptide
sven0666	-	-	-	Y	-
sven0689	-	-	-	Y	-
sven0693	-	-	-	Y	-
sven0712	-	-	-	Y	-
sven0719	-	-	-	Y	-
sven0724	-	-	-	Y	-
sven0735	-	Y	-	-	-

sven0738	-	-	-	Y	-
sven0741	-	-	-	Y	-
sven0747	-	-	-	Y	-
sven0767	-	Y	-	-	Indole
sven0787	-	-	-	Y	-
sven0826	-	-	-	Y	-
sven0827	-	-	-	Y	-
sven0842	-	-	-	Y	-
sven0843	-	-	-	Y	-
sven0856	-	-	-	Y	-
sven0867	-	-	-	Y	-
sven0868	-	-	-	Y	-
sven0879	-	-	-	Y	-
sven0891	-	-	-	Y	-
sven0898	-	-	-	Y	-
sven0904	Y	-	-	-	-
sven0912	-	-	-	Y	-
sven0913	-	-	-	Y	-
sven0916	-	-	-	Y	-
sven0936	-	-	-	Y	-
sven0954	-	-	-	Y	-
sven0964	-	-	-	Y	-
sven1046	-	-	-	Y	-
sven1050	-	-	-	Y	-
sven1064	-	-	-	Y	-
sven1065	-	-	-	Y	-
sven1075	-	-	-	Y	-
sven1079	Y	-	-	-	-
sven1094	-	-	-	Y	-
sven1098	Y	-	-	-	-
sven1105	-	-	-	Y	-
sven1116	Y	-	-	-	-
sven1128	-	-	-	Y	-
sven1130	Y	Y	-	-	-
sven1131	-	Y	-	-	-
sven1132	-	-	-	Y	-
sven1158	Y	-	-	-	-
sven1160	-	Y	-	-	-
sven1167	-	-	-	Y	-
sven1178	-	-	-	Y	-
sven1184	-	-	-	Y	-
sven1187	-	-	-	Y	-

sven1197	-	Y	-	-	-
sven1202	-	-	-	Y	-
sven1230	-	-	-	Y	-
sven1237	Y	-	-	-	-
sven1239	Y	-	-	-	-
sven1242	-	-	-	Y	-
sven1252	Y	-	-	-	-
sven1262	-	-	-	Y	-
sven1283	-	-	-	Y	-
sven1316	-	-	-	Y	-
sven1372	-	Y	-	-	-
sven1373	-	Y	-	-	-
sven1384	-	-	-	Y	-
sven1396	-	-	-	Y	-
sven1413	-	-	-	Y	-
sven1421	-	-	-	Y	-
sven1432	-	-	-	Y	-
sven1440	-	-	-	Y	-
sven1447	-	Y	-	-	-
sven1448	-	Y	-	-	-
sven1459	-	Y	-	-	-
sven1464	-	-	-	Y	-
sven1472	-	-	-	Y	-
sven1477	-	Y	-	-	-
sven1478	-	Y	-	-	-
sven1489	-	Y	-	-	-
sven1508	-	-	-	Y	-
sven1517	-	-	-	Y	-
sven1528	-	-	-	Y	-
sven1531	-	-	-	Y	-
sven1536	-	-	-	Y	-
sven1561	-	-	-	Y	-
sven1569	-	-	-	Y	-
sven1593	-	-	-	Y	-
sven1619	-	-	-	Y	-
sven1626	-	-	-	Y	-
sven1638	-	-	-	Y	-
sven1661	-	-	-	Y	-
sven1663	-	-	-	Y	-
sven1669	-	-	-	Y	-
sven1675	-	-	-	Y	-
sven1677	Y	-	-	-	-

sven1690	-	-	-	Y	-
sven1694	Y	-	-	-	-
sven1699	-	-	-	Y	-
sven1735	-	-	-	Y	-
sven1760	-	-	-	Y	-
sven1774	-	Y	-	-	-
sven1778	-	-	-	Y	-
sven1785	-	-	-	Y	-
sven1792	-	-	-	Y	-
sven1801	-	-	-	Y	-
sven1803	-	-	-	Y	-
sven1805	Y	-	-	-	-
sven1809	Y	-	-	-	-
sven1810	-	-	-	Y	-
sven1812	Y	-	-	-	-
sven1821	Y	-	-	-	-
sven1828	-	-	-	Y	-
sven1829	Y	-	-	-	-
sven1834	-	-	-	Y	-
sven1843	-	-	-	Y	-
sven1857	-	-	-	Y	-
sven1865	-	-	-	Y	-
sven1866	Y	-	-	-	-
sven1868	-	-	-	Y	-
sven1870	-	-	-	Y	-
sven1887	-	-	-	Y	-
sven1892	Y	-	-	-	-
sven1897	-	-	-	Y	-
sven1933	-	-	-	Y	-
sven1949	-	-	-	Y	-
sven1953	-	-	-	Y	-
sven1961	-	-	-	Y	-
sven1962	-	Y	-	-	-
sven1964	-	-	-	Y	-
sven1981	-	-	-	Y	-
sven1989	-	-	-	Y	-
sven1997	-	-	-	Y	-
sven2033	-	-	-	Y	-
sven2059	-	-	-	Y	-
sven2065	-	-	-	Y	-
sven2067	-	-	-	Y	-
sven2072	-	-	-	Y	-

sven2095	-	-	-	Y	-
sven2151	-	-	-	Y	-
sven2152	-	-	-	Y	-
sven2172	-	-	-	Y	-
sven2175	-	-	-	Y	-
sven2178	Y	-	-	-	-
sven2181	-	-	-	Y	-
sven2190	-	-	-	Y	-
sven2195	-	-	-	Y	-
sven2209	Y	-	-	-	-
sven2210	-	-	-	Y	-
sven2229	-	-	-	Y	-
sven2235	-	-	-	Y	-
sven2262	-	-	-	Y	-
sven2270	-	-	-	Y	-
sven2281	-	-	-	Y	-
sven2284	-	-	-	Y	-
sven2292	-	-	-	Y	-
sven2293	-	-	-	Y	-
sven2297	-	-	-	Y	-
sven2298	-	-	-	Y	-
sven2315	-	-	-	Y	-
sven2325	-	-	-	Y	-
sven2338	-	-	-	Y	-
sven2341	Y	-	-	-	-
sven2365	-	-	-	Y	-
sven2371	Y	-	-	-	-
sven2380	-	-	-	Y	-
sven2386	Y	-	-	-	-
sven2394	-	-	-	Y	-
sven2405	-	-	-	Y	-
sven2409	-	-	-	Y	-
sven2416	-	-	-	Y	-
sven2419	-	-	-	Y	-
sven2438	-	-	-	Y	-
sven2442	-	-	-	Y	-
sven2450	-	-	-	Y	-
sven2451	-	-	-	Y	-
sven2516	-	-	-	Y	-
sven2518	-	-	-	Y	-
sven2521	-	-	-	Y	-
sven2550	-	-	-	Y	-

sven2552	Y	-	-	-	-
sven2557	-	-	-	Y	-
sven2582	-	-	-	Y	-
sven2586	-	-	-	Y	-
sven2612	-	-	-	Y	-
sven2639	-	Y	-	-	-
sven2642	-	-	-	Y	-
sven2667	-	-	-	Y	-
sven2675	-	-	-	Y	-
sven2678	Y	Y	-	-	-
sven2683	-	-	-	Y	-
sven2688	-	-	-	Y	-
sven2691	-	-	-	Y	-
sven2696	-	-	-	Y	-
sven2719	-	-	-	Y	-
sven2723	-	-	-	Y	-
sven2750	-	-	-	Y	-
sven2753	-	-	-	Y	-
sven2754	Y	-	-	-	-
sven2760	-	-	-	Y	-
sven2761	-	-	-	Y	-
sven2785	Y	-	-	-	-
sven2793	Y	-	-	-	-
sven2799	-	-	-	Y	-
sven2814	-	-	-	Y	-
sven2829	-	-	-	Y	-
sven2833	Y	-	-	-	-
sven2835	-	-	-	Y	-
sven2837	-	Y	-	-	-
sven2840	-	-	-	Y	-
sven2850	-	-	-	Y	-
sven2878	-	-	-	Y	-
sven2879	-	-	-	Y	-
sven2886	-	-	-	Y	-
sven2889	-	-	-	Y	-
sven2890	-	-	-	Y	-
sven2895	-	-	-	Y	-
sven2967	-	-	-	Y	-
sven2992	-	-	-	Y	-
sven3000	-	-	-	Y	-
sven3008	-	-	-	Y	-
sven3009	-	-	-	Y	-

sven3029	-	-	-	Y	-
sven3035	-	Y	-	-	-
sven3036	-	Y	-	-	-
sven3037	-	-	-	Y	-
sven3038	-	-	-	Y	-
sven3043	-	-	-	Y	-
sven3044	-	-	-	Y	-
sven3050	-	-	-	Y	-
sven3056	-	-	-	Y	-
sven3064	-	-	-	Y	-
sven3077	Y	-	-	-	-
sven3080	-	-	-	Y	-
sven3102	-	Y	-	-	-
sven3120	-	-	-	Y	-
sven3126	-	Y	-	-	-
sven3127	-	Y	-	-	-
sven3146	-	-	-	Y	-
sven3148	-	-	-	Y	-
sven3150	-	-	-	Y	-
sven3152	-	-	-	Y	-
sven3160	-	-	-	Y	-
sven3162	-	-	-	Y	-
sven3165	-	-	-	Y	-
sven3175	-	-	-	Y	-
sven3176	-	-	-	Y	-
sven3179	Y	-	-	-	-
sven3180	Y	-	-	Y	-
sven3209	-	-	-	Y	-
sven3224	-	Y	-	-	-
sven3237	-	-	-	Y	-
sven3247	-	-	-	Y	-
sven3254	-	-	-	Y	-
sven3260	-	-	-	Y	-
sven3273	-	-	-	Y	-
sven3286	-	-	-	Y	-
sven3293	-	-	-	Y	-
sven3300	-	-	-	Y	-
sven3320	-	-	-	Y	-
sven3329	-	-	-	Y	-
sven3338	-	-	-	Y	-
sven3340	-	-	-	Y	-
sven3348	-	Y	-	-	-

sven3349	-	Y	-	-	-
sven3360	-	-	-	Y	-
sven3368	-	-	-	Y	-
sven3370	-	-	-	Y	-
sven3371	Y	-	-	-	-
sven3388	-	-	-	Y	-
sven3423	-	-	-	Y	-
sven3433	Y	-	-	-	-
sven3469	-	-	-	Y	-
sven3473	-	-	-	Y	-
sven3485	Y	-	-	-	-
sven3490	-	-	-	Y	-
sven3505	-	-	-	Y	-
sven3515	-	-	-	Y	-
sven3525	-	Y	-	-	-
sven3533	-	Y	-	-	-
sven3535	-	-	-	Y	-
sven3551	-	-	-	Y	-
sven3566	Y	-	-	-	-
sven3571	-	-	-	Y	-
sven3577	-	-	-	Y	-
sven3579	-	-	-	Y	-
sven3593	-	-	-	Y	-
sven3606	-	Y	-	-	-
sven3610	-	Y	-	-	-
sven3611	-	-	-	Y	-
sven3613	-	-	-	Y	-
sven3627	Y	-	-	-	-
sven3643	-	Y	-	-	-
sven3644	-	Y	-	-	-
sven3657	-	-	-	Y	-
sven3659	Y	-	-	-	-
sven3696	-	-	-	Y	-
sven3706	-	-	-	Y	-
sven3711	-	-	-	Y	-
sven3712	-	-	-	Y	-
sven3715	-	-	-	Y	-
sven3717	-	-	-	Y	-
sven3718	-	-	-	Y	-
sven3719	-	-	-	Y	-
sven3721	-	-	-	Y	-
sven3729	-	-	-	Y	-

sven3763	-	-	-	Y	-
sven3768	-	-	-	Y	-
sven3796	-	-	-	Y	-
sven3814	-	-	-	Y	-
sven3837	-	-	-	Y	-
sven3846	-	-	-	Y	-
sven3847	-	-	-	Y	-
sven3854	-	-	-	Y	-
sven3859	-	-	-	Y	-
sven3874	-	-	-	Y	-
sven3891	-	-	-	Y	-
sven3893	-	-	-	Y	-
sven3894	-	-	-	Y	-
sven3895	Y	-	-	-	-
sven3925	Y	-	-	-	-
sven3961	-	-	-	Y	-
sven3963	-	-	-	Y	-
sven3966	-	-	-	Y	-
sven3982	-	-	-	Y	-
sven3983	-	-	-	Y	-
sven4021	-	-	-	Y	-
sven4025	-	-	-	Y	-
sven4033	-	-	-	Y	-
sven4036	-	-	-	Y	-
sven4042	-	-	-	Y	-
sven4043	Y	-	-	-	-
sven4066	-	-	-	Y	-
sven4089	-	-	-	Y	-
sven4090	-	-	-	Y	-
sven4095	-	-	-	Y	-
sven4103	-	-	-	Y	-
sven4130	Y	-	-	-	-
sven4135	-	-	-	Y	-
sven4138	-	-	-	Y	-
sven4149	-	-	-	Y	-
sven4199	-	-	-	Y	-
sven4204	-	-	-	Y	-
sven4205	-	-	-	Y	-
sven4211	-	-	-	Y	-
sven4227	Y	-	-	-	-
sven4231	-	-	-	Y	-
sven4262	-	-	-	Y	-

sven4267	Y	-	-	-	-
sven4270	Y	-	-	-	-
sven4281	-	-	-	Y	-
sven4283	-	-	-	Y	-
sven4285	-	-	-	Y	-
sven4288	-	-	-	Y	-
sven4296	-	-	-	Y	-
sven4319	Y	-	-	-	-
sven4333	-	-	-	Y	-
sven4337	Y	-	-	-	-
sven4346	Y	-	-	-	-
sven4361	-	-	-	Y	-
sven4364	Y	-	-	-	-
sven4374	-	-	-	Y	-
sven4407	Y	-	-	-	-
sven4426	-	Y	-	-	-
sven4444	-	-	-	Y	-
sven4451	-	-	-	Y	-
sven4456	-	-	-	Y	-
sven4458	Y	-	-	-	-
sven4468	-	Y	-	-	-
sven4469	-	-	-	Y	-
sven4472	-	-	-	Y	-
sven4498	Y	-	-	-	-
sven4518	-	-	-	Y	-
sven4521	Y	-	-	-	-
sven4544	Y	-	-	-	-
sven4590	-	-	-	Y	-
sven4592	-	-	-	Y	-
sven4600	-	-	-	Y	-
sven4609	-	-	-	Y	-
sven4613	-	Y	-	-	-
sven4614	-	Y	-	-	-
sven4621	-	-	-	Y	-
sven4629	-	-	-	Y	-
sven4631	-	-	-	Y	-
sven4671	-	-	-	Y	-
sven4724	-	-	-	Y	-
sven4747	-	-	-	Y	-
sven4751	-	-	-	Y	-
sven4789	-	Y	-	-	-
sven4792	-	-	-	Y	-

sven4795	-	-	-	Y	-
sven4810	-	-	-	Y	-
sven4813	-	Y	-	-	-
sven4814	-	Y	-	-	-
sven4833	-	-	-	Y	-
sven4844	-	Y	-	-	-
sven4847	Y	-	-	-	-
sven4863	-	-	-	Y	-
sven4869	-	-	-	Y	-
sven4898	-	-	-	Y	-
sven4907	-	-	-	Y	-
sven4943	-	-	-	Y	-
sven4944	-	-	-	Y	-
sven4946	-	Y	-	-	-
sven4954	-	-	-	Y	-
sven4957	-	-	-	Y	-
sven4980	-	-	-	Y	-
sven4983	-	-	-	Y	-
sven5031	-	-	-	Y	-
sven5041	-	-	-	Y	-
sven5062	-	-	-	Y	-
sven5072	Y	-	-	-	-
sven5093	-	-	-	Y	Butyrolactone
sven5118	-	-	-	Y	-
sven5157	-	-	-	Y	-
sven5160	-	-	-	Y	-
sven5207	-	-	-	Y	-
sven5253	-	-	-	Y	-
sven5256	-	-	-	Y	-
sven5258	-	-	-	Y	-
sven5271	-	-	-	Y	-
sven5283	-	-	-	Y	-
sven5302	-	-	-	Y	-
sven5368	-	-	-	Y	Polyketide
sven5390	-	Y	-	-	-
sven5393	-	-	-	Y	-
sven5395	-	-	-	Y	-
sven5405	-	-	-	Y	-
sven5414	-	-	-	Y	Siderophore
sven5420	-	-	-	Y	Siderophore
sven5426	-	-	-	Y	-
sven5451	-	-	-	Y	-

sven5462	-	-	-	Y	-
sven5480	-	-	-	Y	Siderophore
sven5500	-	-	-	Y	-
sven5522	-	-	-	Y	-
sven5528	-	-	-	Y	-
sven5561	-	-	-	Y	-
sven5571	-	-	-	Y	-
sven5596	-	-	-	Y	-
sven5656	-	-	-	Y	-
sven5682	-	-	-	Y	-
sven5686	-	-	-	Y	-
sven5700	-	-	-	Y	-
sven5709	-	-	-	Y	-
sven5712	-	-	-	Y	-
sven5715	-	-	-	Y	-
sven5721	-	-	-	Y	-
sven5767	-	-	-	Y	-
sven5780	-	-	-	Y	-
sven5798	-	-	-	Y	-
sven5802	-	-	-	Y	-
sven5809	-	-	-	Y	-
sven5860	-	-	-	Y	-
sven5862	-	-	-	Y	-
sven5871	-	-	-	Y	-
sven5875	-	-	-	Y	-
sven5897	-	-	-	Y	-
sven5904	-	-	-	Y	-
sven5931	-	-	-	Y	-
sven5936	-	-	-	Y	-
sven5955	Y	-	-	-	-
sven5960	-	-	-	Y	-
sven5969	Y	-	-	-	Butyrolactone / polyketide
sven6004	-	-	-	Y	-
sven6027	-	-	-	Y	-
sven6048	-	-	-	Y	-
sven6055	-	-	-	Y	-
sven6104	-	-	-	Y	-
sven6109	-	-	-	Y	-
sven6119	-	-	-	Y	-
sven6142	-	-	-	Y	-
sven6156	-	-	-	Y	-
sven6180	-	-	-	Y	Non-

					ribosomal peptide
sven6209	-	-	-	Y	Non-ribosomal peptide
sven6213	-	-	-	Y	Non-ribosomal peptide
sven6264	-	-	-	Y	Non-ribosomal peptide
sven6310	-	-	-	Y	-
sven6361	-	-	-	Y	-
sven6381	-	-	-	Y	-
sven6387	-	-	-	Y	-
sven6396	-	-	-	Y	-
sven6397	-	-	-	Y	-
sven6404	-	-	-	Y	-
sven6430	-	-	-	Y	-
sven6434	-	-	-	Y	-
sven6435	-	Y	-	-	-
sven6437	-	-	-	Y	Terpene
sven6443	-	-	-	Y	Terpene
sven6455	Y	-	-	-	Terpene
sven6456	Y	-	-	-	Terpene
sven6457	-	-	-	Y	Terpene
sven6460	-	-	-	Y	Terpene
sven6464	-	-	-	Y	-
sven6519	-	-	-	Y	-
sven6523	-	-	-	Y	-
sven6526	-	-	-	Y	-
sven6531	-	-	-	Y	Bacteriocin
sven6564	-	-	-	Y	-
sven6580	-	-	-	Y	-
sven6621	-	-	-	Y	-
sven6631	-	-	-	Y	-
sven6657	-	Y	-	-	-
sven6668	-	-	-	Y	-
sven6673	-	-	-	Y	-
sven6751	-	-	-	Y	-
sven6784	-	-	-	Y	-
sven6801	-	-	-	Y	-
sven6803	-	-	-	Y	-
sven6826	Y	-	-	-	-
sven6833	-	-	-	Y	-

sven6840	-	-	-	Y	Melanin
sven6863	-	-	-	Y	-
sven6866	-	-	-	Y	-
sven6872	-	-	-	Y	-
sven6873	-	-	-	Y	-
sven6885	-	-	-	Y	-
sven6900	-	-	-	Y	-
sven6901	Y	-	-	-	-
sven6935	-	-	-	Y	-
sven6936	-	-	-	Y	-
sven6949	-	-	-	Y	-
sven6957	Y	-	-	-	-
sven7023	-	-	-	Y	-
sven7111	-	-	-	Y	Terpene
sven7226	-	-	-	Y	Polyketide
sven7271	-	-	-	Y	-
sven7272	-	-	-	Y	-
sven7281	-	-	-	Y	-
sven7282	Y	-	-	-	-
sven7289	-	-	-	Y	-
sven7294	-	-	-	Y	-
sven7301	-	-	-	Y	-
sven7316	Y	-	-	-	-
sven7317	-	-	-	Y	-
sven7324	-	-	-	Y	-
sven7335	Y	-	-	-	-
sven7351	-	-	-	Y	-
sven7396	-	-	-	Y	-
sven7418	-	-	-	Y	-
sven7451	-	-	-	Y	Terpene / non-ribosomal peptide
sven7453	-	-	-	Y	-

*Unclassified due to ambiguous transcription start sites and/or uneven coverage

[x] Location of clusters as per Nett *et al.* (2009) for *S. avermitilis* and *S. coelicolor,* and as determined using the program antiSMASH [Medema *et al.* (2011)] for *S. venezuelae.*

[a] Previously identified by Vockenhuber *et al.* (2011)

[b] Previously identified by Swiercz *et al.* (2008)

[c] Previously identified by D’Alia *et al.* (2010)

[d] Upregulated (or upregulated cutoRNA partner) in an RNase III mutant, as shown by Gatewood *et al.* (2012).

References:

D'Alia D, Nieselt K, Steigele S, Müller J, Verburg I, et al (2010) Noncoding RNA of glutamine synthetase I moduelates antibiotic production in *Streptomyces coelicolor* A3(2). J Bacteriol 192: 1160-1164.

Gatewood ML, Bralley P, Weil MR, Jones GH (2012) RNA-Seq and RNA immunoprecipitation analyses of the transcriptome of *Streptomyces coelicolor* identify substrates for RNase III. J Bacteriol 194: 2228-2237.

Medema MH, Blin K, Cimermancic P, de Jager V, Zakrzewski P, et al. (2011) antiSMASH: rapid identification, annotation and analysis of secondary metabolite biosynthesis gene clusters in bacterial and fungal genome sequences. Nucleic Acids Res. doi: 10.1093/nar/gkr466

Nett M, Ikeda H, Moore BS (2009) Genomic basis for natural product biosynthetic diversity in the actinomycetes. Nat Prod Rep 26: 1362-1384

Swiercz JP, Hindra, Bobek J, Haiser HJ, DiBerardo C, et al. (2008) Small non-coding RNAs in *Streptomyces coelicolor.* Nucleic Acid Res. 36: 7240-7251.

Vockenhuber M-P, Sharma CM, Statt MG, Schmidt D, Xu Z, et al. (2011) Deep sequencing-based identification of small non-coding RNAs in *Streptomyces coelicolor.* RNA Biology 8: 468-477.

Table S2. Conserved cutoRNA gene pairs.

Conserved cutoRNA gene pairs					Conservation of convergent gene arrangement*				
S. coelicolor	***S. avermitilis***	***S. venezuelae***	**Gene 1 annotation**	**Gene 2 annotation**	***S. griseus***	***S. scabies***	***T. fusca***	***M. tb***	***F. alni***
sco1294 / sco1293	*sav7062 / sav7063*	*sven0868 / sven0867*	putative cystathionine gamma-synthase	hypothetical protein	Y	Y	NH	NH	NH
sco1841 / sco1842	*sav6424 / sav6423*	*sven1488 / sven1489*	hypothetical protein	hypothetical protein	Y	Y	NH	NH	NH
sco2114 / sco2113	*sav6087 / sav6088*	*sven1774 / sven1773*	hypothetical protein	putative bacterioferritin	Y	Y	NH	NH	N
sco2267 / sco2268	*sav5930 / sav5929*	*sven1952 / sven1953*	probable heme oxygenase	conserved hypothetical protein	Y	Y	NH	NH	NH
sco2582 / sco2583	*sav5476 / sav5475*	*sven2364 / sven2365*	conserved hypothetical protein SCC123.20.	putative membrane protein.	Y	Y	NH	NH	NH
sco3134 / sco3133	*sav3572 / sav3571*	*sven2957 / sven2956*	putative two-component system response regulator	putative MarR-family regulator	Y	Y	NH	NH	NH
sco3375 / sco3374	*sav4695 / sav4696*	*sven3225 / sven3224*	putative Lsr2-like protein	hypothetical proline-rich protein	Y	Y	NH	NH	NH
sco3579 / sco3578	*sav4584 / sav4585*	*sven3349 / sven3348*	wblA	putative ion-transporting ATPase	Y	Y	Y	Y	Y
sco3008 / sco3007	*sav5068 / sav5069*	*sven2751 / sven2750*	putative two-component system response regulator	conserved hypothetical protein	Y	Y	NH	NH	NH
sco3190 / sco3191	*sav3681 / sav3682*	*sven3035 / sven3036*	large-conductance mechanosensitive channel	hypothetical protein	N	Y	NH	NH	NH
sco3833 / sco3832	*sav4360 / sav4361*	*sven3607 / sven3606*	transcriptional regulator, TetR family	putative transcriptional regulator	Y	Y	NH	NH	NH
sco4282 / sco4283	*sav3943 / sav3942*	*sven4035 / sven4036*	putative dimeric protein	putative tagatose-6-phosphate kinase	Y	Y	NH	NH	NH
sco4335 / sco4334	*sav3896 / sav3897*	*sven4150 / sven4149*	hypothetical protein	putative hypoxanthine / guanine permease	Y	Y	NH	NH	NH
sco5167 / sco5168	*sav3097 / sav3096*	*sven4813 / sven4814*	hypothetical protein	hypothetical protein	Y	Y	NH	NH	NH
sco5255 / sco5256	*sav2987 / sav2986*	*sven4945 / sven4946*	signal peptidase protein	hypothetical protein	Y	Y	NH	NH	NH
sco5390 / sco5389	*sav2865 / sav2866*	*sven5041 / sven5040*	putative luciferase-family protein	hypothetical protein	Y	Y	NH	NH	N
sco6750 / sco6749	*sav1663 / sav1664*	*sven6435 / sven6434*	isopentenyl-diphosphate delta-isomerase	hypothetical protein	Y	Y	NH	NH	NH
sco6771 / sco6772	*sav1643 / sav1642*	*sven6456 / sven6457*	putative small hydrophobic secreted protein	putative protein tyrosine phosphatase	Y	Y	NH	NH	NH
sco7252 / sco7253	*sav1236 / sav1235*	*sven0266 / sven0265*	putative regulatory protein	hypothetical protein	Y	Y	NH	NH	NH

***** (Y) – YES
(N) – NO
(NH) - At least one of the two genes does not have homologues in the given species;

Table S3: Intergenic sRNAs in *S. coelicolor*, *S. avermitilis*, and *S. venezuelae*.

Species	sRNA name	Left Gene	Right Gene	Strand	Start	End	Size	Located within biosynthetic cluster[y]	Previously identified or predicted
S. coelicolor									
	scr0636	*sco0635*	*sco0636*	+	677033	677230	198		
	scr0792	*sco0791*	*sco0792*	+	838750	838863	114		
	scr0959	*sco0958*	*sco0959*	+	1008731	1008802	72		
	scr0999	*sco0998*	*sco0999*	-	1054736	1054647	90		
	scr1104	*sco1103*	*sco1104*	-	1161700	1161580	121		Vockenhuber et al (2011)
	scr1363	*sco1362*	*sco1363*	-	1441015	1440924	92		
	scr1390*	*sco1389*	*sco1390*	-	1474104	1473910	195		
	scr1424	*sco1423*	*sco1424*	-	1519780	1519565	216		
	scr1434	*sco1433*	*sco1434*	+	1529418	1529480	63		
	scr1594	*sco1593*	*sco1594*	+	1703984	1704169	186		
	scr1792*	*sco1791*	*sco1792*	-	1922085	1921800	286		Panek et al (2008)
	scr1805	*sco1804*	*sco1805*	-	1933162	1932972	191		
	scr1961	*sco1960*	*sco1961*	+	2099076	2099145	70		
	scr2101	*sco2100*	*sco2101*	-	2258212	2257982	231		Swiercz et al (2008)
	scr2107	*sco2106*	*sco2107*	+	2264717	2264823	107		
	scr2117	*sco2116*	*sco2117*	+	2276540	2276611	72		
	scr2136	*sco2135*	*sco2136*	-	2297658	2297564	95		
	scr2147	*sco2146*	*sco2147*	-	2308772	2308678	95		
	scr2257	*sco2256*	*sco2257*	+	2424206	2424263	58		
	scr2288	*sco2287*	*sco2288*	+	2458925	2458996	72		
	scr2446	*sco2445*	*sco2446*	+	2625429	2625650	222		
	scr2589	*sco2588*	*sco2589*	+	2725406	2725465	60		
	scr2528	*sco2527*	*sco2528*	+	2725412	2725465	54		
	scr2634	*sco2633*	*sco2634*	+	2860742	2860810	69		
	scr2736	*sco2735*	*sco2736*	+	2982223	2982670	448		Swiercz et al (2008); Vockenhuber et al (2011)
	scr2750	*sco2749*	*sco2750*	-	2996439	2996338	102		
	scr2822	*sco2821*	*sco2822*	+	3082275	3082345	71		Panek et al (2008)
	scr2922	*sco2921*	*sco2922*	-	3174924	3174429	496		
	scr2943	*sco2942*	*sco2943*	+	3198596	3198780	185		
	scr2952	*sco2951*	*sco2952*	+	3208719	3208802	84		Vockenhuber et al (2011)
	scr3020*	*sco3019*	*sco3020*	-	3302281	3302024	258		
	scr3077	*sco3076*	*sco3077*	-	3370220	3369991	230		
	scr3097	*sco3096*	*sco3097*	+	3391994	3392073	80		
	scr3202	*sco3201*	*sco3202*	-	3510186	3510094	93		Vockenhuber et al

									(2011)
	scr3253	*sco3252*	*sco3253*	+	3605514	3605596	83		
	scr3334*	*sco3333*	*sco3334*	-	3691139	3690886	254		Panek et al (2008)
	scr3410	*sco3409*	*sco3410*	-	3775694	3775649	46		
	scr3437	*sco3436*	*sco3437*	-	3799361	3799139	223		
	scr3558	*sco3557*	*sco3558*	-	3933668	3933527	142		Panek et al (2008); Swiercz et al (2008); Vockenhuber et al (2011)
	scr3559	*sco3558*	*sco3559*	+	3934693	3934919	227		Panek et al (2008); Vockenhuber et al (2011)
	scr3580	*sco3579*	*sco3580*	-	3958172	3957843	330		Vockenhuber et al (2011)
	scr3603	*sco3602*	*sco3603*	-	3979771	3979385	387		
	scr3679	*sco3678*	*sco3679*	-	4061931	4061681	251		
	scr3716	*sco3715*	*sco3716*	-	4091239	4091112	128		
	scr3901	*sco3900*	*sco3901*	+	4295743	4296186	444		
	scr3920	*sco3919*	*sco3920*	+	4315358	4315489	132		Vockenhuber et al (2011)
	scr3929	*sco3928*	*sco3929*	+	4323850	4324377	528		
	scr3931	*sco3930*	*sco3931*	-	4326774	4326705	70		
	scr4076*	*sco4075*	*sco4076*	-	4470108	4470036	73		Panek et al (2008)
	scr4108	*sco4107*	*sco4108*	+	4507134	4507300	167		
	scr4123*	*sco4122*	*sco4123*	+	4530078	4530310	233		
	scr4135	*sco4134*	*sco4135*	-	4550061	4549825	237		
	scr4139	*sco4138*	*sco4139*	-	4553616	4553538	79		
	scr4220	*sco4219*	*sco4220*	-	4627309	4627161	149		
	scr4233	*sco4232*	*sco4233*	-	4637485	4637326	160		
	scr4339	*sco4338*	*sco4339*	+	4753312	4753401	90		
	scr4389	*sco4388*	*sco4389*	-	4805789	4805580	210		Swiercz et al (2008); Vockenhuber et al (2011)
	scr4395	*sco4394*	*sco4395*	-	4811827	4811713	115		
	scr4412	*sco4411*	*sco4412*	-	4829635	4829349	287		
	scr4524	*sco4523*	*sco4524*	-	4944655	4944415	241		
	scr4619	*sco4618*	*sco4619*	+	5043143	5043220	78		
	scr4628	*sco4627*	*sco4628*	+	5049482	5049538	57		
	scr4632	*sco4631*	*sco4632*	+	5054815	5055060	246		Vockenhuber et al (2011)
	scr4677	*sco4676*	*sco4677*	+	5108129	5108199	71		Swiercz et al (2008)
	scr4684	*sco4683*	*sco4684*	+	5113596	5113760	165		

	scr4689	*sco4688*	*sco4689*	-	5117055	5116751	305		
	scr4909	*sco4908*	*sco4909*	+	5341263	5341370	108		
	scr5028	*sco5027*	*sco5028*	+	5462580	5462628	49		Swiercz et al (2008)
	scr5112	*sco5111*	*sco5112*	-	5556709	5556524	186		
	scr5239	*sco5238*	*sco5239*	+	5699820	5700035	216		
	scr5346	*sco5345*	*sco5346*	-	5814859	5814784	76		
	scr5439	*sco5438*	*sco5439*	-	5912258	5912163	96		Panek et al (2008)
	scr5583	*sco5582*	*sco5583*	+	6085733	6085815	83		
	scr5633	*sco5632*	*sco5633*	+	6133070	6133458	389		
	scr5676	*sco5675*	*sco5676*	-	6176413	6176160	254		Swiercz et al (2008); Vockenhuber et al (2011)
	scr5710	*sco5709*	*sco5710*	-	6221571	6221499	73		
	scr5839	*sco5838*	*sco5839*	+	6392117	6392286	170		
	scr5917	*sco5916*	*sco5917*	+	6484345	6484908	564		
	scr5922	*sco5921*	*sco5922*	-	6489972	6489791	182		
	scr5999	*sco5998*	*sco5999*	-	6575212	6574790	423		
	scr6106	*sco6105*	*sco6106*	+	6706591	6706667	77		Vockenhuber et al (2011)
	scr6154	*sco6153*	*sco6154*	+	6755084	6755550	467		
	scr6204	*sco6203*	*sco6204*	-	6818847	6818680	168		
	scr6214	*sco6213*	*sco6214*	+	6828082	6828120	39		
	scr6287	*sco6286*	*sco6287*	-	6946592	6946547	46	Yellow cryptic polyketide	
	scr6925	*sco6924*	*sco6925*	+	7688336	7688467	132		Vockenhuber et al (2011)
	scr7098	*sco7097*	*sco7098*	+	7888419	7888550	132		
	scr7204	*sco7203*	*sco7204*	+	8006662	8007032	371		
	scr7213	*sco7212*	*sco7213*	+	8016644	8016820	177		
	scr7214	*sco7213*	*sco7214*	+	8017756	8017850	95		
	scr7434	*sco7433*	*sco7434*	-	8247159	8247075	85		
	scr7776	*sco7775*	*sco7776*	+	8604074	8604158	85		
S. avermitilis									
	sar0048	*sav47*	*sav48*	+	57774	57862	89		
	sar0059	*sav58*	*sav59*	+	67588	67733	146		
	sar0088	*sav87*	*sav88*	+	98161	98540	380		
	sar0170	*sav169*	*sav170*	+	189927	189977	51		
	sar0212	*sav211*	*sav212*	+	247123	247236	114		
	sar0245	*sav244*	*sav245*	-	282713	282594	120		
	sar0250	*sav249*	*sav250*	-	288680	288607	74		
	sar0273	*sav272*	*sav273*	-	320477	320276	202		
	sar0287	*sav286*	*sav287*	-	336631	336553	79		
	sar7594[x]	*sav312*	*sav7594*	+	370213	370258	46		

	sar0324_1	*sav323*	*sav324*	-	382268	382160	109		
	sar0324_2	*sav323*	*sav324*	-	382428	382371	58		
	sar0324_3	*sav323*	*sav324*	-	382612	382447	166		
	sar0351	*sav350*	*sav351*	-	412386	412056	331		
	sar0353	*sav352*	*sav353*	-	414792	414750	43		
	sar0444	*sav443*	*sav444*	-	598309	598225	85		
	sar0466	*sav465*	*sav466*	+	616811	616926	116		
	sar7577[x]	*sav504*	*sav7577*	-	648062	647767	296		
	sar0625	*sav624*	*sav625*	-	777160	777061	100		
	sar0634	*sav633*	*sav634*	-	788238	788113	126		
	sar0709	*sav708*	*sav709*	-	864406	864174	233		
	sar0742	*sav741*	*sav742*	-	890982	890920	63		
	sar0778	*sav777*	*sav778*	+	927129	927220	92		
	sar0805	*sav804*	*sav805*	-	958110	958026	85		
	sar0823	*sav822*	*sav823*	+	973852	973976	125		
	sar0829	*sav828*	*sav829*	+	982914	982983	70		
	sar0959	*sav958*	*sav959*	-	1219321	1219274	48		
	sar0964	*sav963*	*sav964*	-	1223354	1223170	185		
	sar0967	*sav966*	*sav967*	-	1225858	1225751	108		
	sar0994	*sav993*	*sav994*	-	1259585	1259487	99		
	sar1055	*sav1054*	*sav1055*	-	1332741	1332671	71		
	sar1077	*sav1076*	*sav1077*	-	1357266	1357224	43		
	sar1106	*sav1105*	*sav1106*	-	1391110	1391029	82		
	sar1129	*sav1128*	*sav1129*	-	1417372	1417296	77		
	sar1190	*sav1189*	*sav1190*	+	1480461	1480530	70		
	sar1249	*sav1248*	*sav1249*	+	1548899	1548993	95	Polyketide / non-ribosomal peptide hybrid	
	sar1289	*sav1288*	*sav1289*	-	1597349	1597206	144		
	sar1474	*sav1473*	*sav1474*	-	1817130	1817100	31		
	sar1503	*sav1502*	*sav1503*	-	1843553	1843465	89		
	sar1553	*sav1552*	*sav1553*	-	1912470	1912379	92	Polyketide	
	sar1571	*sav1570*	*sav1571*	+	1935799	1935844	46		
	sar1655	*sav1654*	*sav1655*	+	2027150	2027205	56	Hopene	
	sar1689	*sav1688*	*sav1699*	+	2066680	2066772	93		
	sar1711	*sav1710*	*sav1711*	-	2091025	2090892	134		
	sar1814	*sav1813*	*sav1814*	+	2216915	2217244	330		
	sar2017	*sav2016*	*sav2017*	-	2458516	2458411	106		
	sar2028	*sav2027*	*sav2028*	+	2468943	2469157	215		
	sar2087	*sav2086*	*sav2087*	-	2541468	2541337	132		
	sar2124	*sav2123*	*sav2124*	-	2590455	2590363	93		
	sar2127	*sav2126*	*sav2127*	+	2594347	2594434	88		
	sar2199	*sav2198*	*sav2199*	+	2673499	2673564	66		
	sar2203	*sav2202*	*sav2203*	+	2682862	2683133	272		
	sar2245	*sav2244*	*sav2245*	+	2732474	2732605	132		

	sar2254	*sav2253*	*sav2254*	+	2748973	2749062	90		
	sar2261	*sav2260*	*sav2261*	+	2758394	2758465	72		
	sar2301	*sav2300*	*sav2301*	+	2803270	2803624	355		
	sar2386	*sav2385*	*sav2386*	-	2911641	2911540	102	Aromatic polyketide	
	sar2406	*sav2405*	*sav2406*	+	2932336	2932772	437		
	sar2421	*sav2420*	*sav2421*	-	2949746	2949603	144		
	sar2515*	*sav2514*	SAV*_2515*	-	3079265	3079081	185		
	sar2517	*sav2516*	*sav2517*	-	3082189	3082129	61		
	sar2548	*sav2547*	*sav2548*	+	3126939	3127014	76		
	sar2551	*sav2550*	*sav2551*	+	3128837	3128898	62		
	sar2567	*sav2566*	*sav2567*	-	3148409	3148337	73		
	sar2627	*sav2626*	*sav2627*	+	3226335	3226392	58		
	sar2652	*sav2651*	*sav2652*	-	3250602	3250521	82		
	sar2765	*sav2764*	*sav2765*	-	3392358	3392270	89		
	sar2770	*sav2769*	*sav2770*	-	3399050	3398950	101		
	sar2808	*sav2807*	*sav2808*	+	3451152	3451231	80		
	sar2924	*sav2923*	*sav2924*	+	3659378	3659414	37		
	sar2966	*sav2965*	*sav2966*	+	3716515	3716601	87		
	sar3006	*sav3005*	*sav3006*	-	3760718	3760638	81		
	sar3017	*sav3016*	*sav3017*	+	3771883	3771800	84		
	sar3018	*sav3017*	*sav3018*	-	3773826	3773483	344		
	sar3107	*sav3106*	*sav3107*	+	3879684	3880075	392		
	sar3140	*sav3139*	*sav3140*	+	3912959	3913036	78		
	sar3145	*sav3144*	*sav3145*	-	3917556	3917496	61		
	sar3164	*sav3163*	*sav3164*	+	3941458	3941806	349	Non-ribosomal peptide	
	sar3238	*sav3237*	*sav3238*	-	4036188	4036140	49		
	sar3338	*sav3337*	*sav3338*	-	4152217	4152150	68		
	sar3386	*sav3385*	*sav3386*	+	4207298	4207426	129		
	sar3482	*sav3481*	*sav3482*	+	4314599	4314690	92		
	sar3510	*sav3509*	*sav3510*	-	4344147	4344001	147		
	sar3573	*sav3572*	*sav3573*	-	4423000	4422844	157		
	sar3677	*sav3676*	*sav3677*	+	4553774	4553929	156		
	sar3679	*sav3678*	*sav3679*	+	4555649	4555702	54		
	sar3694_1	*sav3693*	*sav3694*	+	4571212	4571486	275		
	sar3694_2	*sav3693*	*sav3694*	-	4571405	4571266	140		
	sar3698	*sav3697*	*sav3698*	-	4575349	4575256	94		
	sar3708_1*	*sav3707*	*sav3708*	+	4590173	4590242	70		
	sar3708_2*	*sav3707*	*sav3708*	-	4590259	4590179	81		
	sar3713	*sav3712*	*sav3713*	+	4594744	4594818	75		
	sar3721	*sav3720*	*sav3721*	+	4602366	4602436	71		
	sar3730	*sav3729*	*sav3730*	+	4617509	4617600	92		
	sar3855	*sav3854*	*sav3855*	+	4762016	4762130	115		
	sar3858	*sav3857*	*sav3858*	+	4765693	4765915	223		
	sar3861	*sav3860*	*sav3861*	-	4769708	4769638	71		

	sar3892	*sav3891*	*sav3892*	-	4808188	4808105	84		
	sar3948	*sav3947*	*sav3948*	+	4872344	4872418	75		
	sar3970	*sav3969*	*sav3970*	+	4895793	4896030	238		
	sar3975	*sav3975*	*sav3976*	-	4901101	4901011	91		
	sar3980	*sav3979*	*sav3980*	+	4906190	4906277	88		
	sar3982	*sav3982*	*sav3983*	+	4909537	4909620	84		
	sar4024	*sav4023*	*sav4024*	+	4945054	4945399	346		
	sar4076	*sav4075*	*sav4076*	+	4996048	4996124	77		
	sar4105_1*	*sav4104*	*sav4105*	-	5024050	5024095	46		
	sar4105_2*	*sav4104*	*sav4105*	-	5029867	5029660	208		
	sar4113	SAV_4112	*sav4113*	-	5043566	5043450	117		
	sar4119*	*sav4118*	*sav4119*	-	5051673	5051507	167		
	sar4142*	*sav4141*	*sav4142*	+	5081598	5081712	115		
	sar4154	*sav4153*	*sav4154*	+	5102609	5102792	184		
	sar4183	*sav4182*	*sav4183*	-	5132493	5132428	66		
	sar4221	*sav4220*	*sav4221*	-	5176975	5176902	74		
	sar4227	*sav4226*	*sav4227*	+	5182448	5182494	47		
	sar4246	*sav4245*	*sav4246*	-	5207192	5207050	143		
	sar4274	*sav4273*	*sav4274*	-	5240107	5240034	74		
	sar4280	*sav4279*	*sav4280*	+	5246032	5246079	48		
	sar4323	*sav4322*	*sav4323*	-	5298534	5298439	96		
	sar4325	*sav4324*	*sav4325*	+	5300359	5300585	227		
	sar4449	*sav4448*	*sav4449*	-	5435097	5434767	331		
	sar4464	*sav4463*	*sav4464*	+	5452987	5453201	215		
	sar4466	*sav4465*	*sav4466*	-	5454893	5454787	107		
	sar4518	*sav4517*	*sav4518*	+	5519730	5520278	549		
	sar4599_1	*sav4598*	*sav4599*	+	5613649	5613695	47		
	sar4599_2	*sav4598*	*sav4599*	-	5613740	5613701	40		
	sar4604	*sav4603*	*sav4604*	-	5622151	5621921	231		
	sar4605	*sav4604*	*sav4605*	+	5623152	5623293	142		
	sar4634	*sav4633*	*sav4634*	+	5657853	5657894	42		
	sar4635	*sav4634*	*sav4635*	-	5659414	5659293	122		
	sar4691	*sav4690*	*sav4691*	+	5717781	5717832	52		
	sar4726*	*sav4725*	*sav4726*	+	5758724	5758952	229		
	sar4755_1	*sav4754*	*sav4755*	+	5798158	5798235	78		
	sar4755_2	*sav4754*	*sav4755*	-	5798244	5798178	67		
	sar4784	*sav4783*	*sav4784*	+	5827430	5827521	92		
	sar5002_1	*sav5001*	*sav5002*	+	6051621	6051861	241		
	sar5002_2	*sav5001*	*sav5002*	-	6052035	6051886	150		
	sar5018	*sav5017*	*sav5018*	-	6075746	6075660	87		
	sar5019	*sav5018*	*sav5019*	-	6076816	6076706	111		
	sar5020	*sav5019*	*sav5020*	-	6077813	6077707	107		
	sar5033	*sav5032*	*sav5033*	-	6092394	6092349	46		
	sar5057*	*sav5056*	*sav5057*	+	6123837	6124050	214		

	sar5126	*sav5125*	*sav5126*	-	6225412	6225322	91			
	sar5162	*sav5161*	*sav5162*	-	6270115	6270026	90			
	sar5217	*sav5216*	*sav5217*	-	6322900	6322737	164			
	sar5282	*sav5281*	*sav5282*	-	6399268	6399222	47			
	sar5316	*sav5315*	*sav5316*	+	6443023	6443119	97			
	sar5331_1	*sav5330*	*sav5331*	-	6460234	6460136	99			
	sar5331_2	*sav5330*	*sav5331*	-	6460569	6460260	310			
	sar5366	*sav5365*	*sav5366*	-	6506449	6506370	80			
	sar5413	*sav5412*	*sav5413*	-	6563407	6563315	93			
	sar5523	*sav5522*	*sav5523*	+	6694234	6694613	380			
	sar5549	*sav5548*	*sav5549*	-	6712176	6712127	50			
	sar5562	*sav5561*	*sav5562*	-	6850201	6850110	92			
	sar5751	*sav5750*	*sav5751*	+	6953178	6953422	245			
	sar5782	*sav5781*	*sav5782*	+	6987407	6987469	63			
	sar5828	*sav5827*	*sav5828*	+	7034388	7034568	181			
	sar5855	*sav5854*	*sav5855*	-	7063523	7063435	89			
	sar5912	*sav5911*	*sav5912*	+	7121977	7122048	72			
	sar5914	*sav5913*	*sav5914*	-	7125394	7125232	163			
	sar6002	*sav6001*	*sav6002*	+	7229111	7229269	159			
	sar6057	*sav6056*	*sav6057*	+	7291229	7291400	172			
	sar6063	*sav6062*	*sav6063*	+	7297145	7297192	48			
	sar6085	*sav6084*	*sav6085*	-	7322414	7322306	109			
	sar6087	*sav6086*	*sav6087*	-	7325372	7325302	71			
	sar6096	*sav6095*	*sav6096*	-	7332956	7332846	111			
	sar6104	*sav6103*	*sav6104*	+	7340361	7340426	66			
	sar6191	*sav6190*	*sav6191*	+	7441504	7441585	82			
	sar6224	*sav6223*	*sav6224*	+	7476616	7476662	47			
	sar6284	*sav6283*	*sav6284*	-	7542085	7542006	80			
	sar6287	*sav6286*	*sav6287*	+	7546861	7546922	62			
	sar6350_1	*sav6349*	*sav6350*	+	7617994	7618066	73			
	sar6350_2	*sav6349*	*sav6350*	+	7618072	7618377	306			
	sar6383	*sav6382*	*sav6383*	-	7656436	7656340	97			
	sar6459	*sav6458*	*sav6459*	-	7736351	7736291	61			
	sar6487*	*sav6486*	*sav6487*	+	7764906	7765176	271			
	sar6642	*sav6641*	*sav6642*	-	7946216	7946167	50			
	sar6650_1	*sav6649*	*sav6650*	+	7954775	7954869	95			
	sar6650_2	*sav6649*	*sav6650*	+	7954913	7954999	87			
	sar6673	*sav6672*	*sav6673*	+	7984373	7984448	76			
	sar6697	*sav6696*	*sav6697*	-	8009565	8009483	83			
	sar6711	*sav6710*	*sav6711*	-	8026341	8026244	98			
	sar6745	*sav6744*	*sav6745*	-	8063459	8063309	151			
	sar6875_1	*sav6874*	*sav6875*	+	8204040	8204175	136			
	sar6875_2	*sav6874*	*sav6875*	+	8204215	8204302	88			
	sar6912	*sav6911*	*sav6912*	-	8246202	8246140	63			

	sar6923	*sav6922*	*sav6923*	+	8259274	8259573	300		
	sar6976*	*sav6975*	*sav6976*	+	8328102	8328240	139		
	sar6989	*sav6988*	*sav6989*	-	8348863	8348818	46		
	sar6990	*sav6989*	*sav6990*	-	8349781	8349748	34		
	sar6991	*sav6990*	*sav6991*	+	8351549	8351628	80		
	sar6998	*sav6997*	*sav6998*	+	8358793	8358966	174		
	sar7100	*sav7099*	*sav7100*	-	8461862	8461743	120		
	sar7183	*sav7182*	*sav7183*	+	8552575	8552613	39		
	sar7207	*sav7206*	*sav7207*	-	8583516	8583427	90		
	sar7256	*sav7255*	*sav7256*	-	8652122	8652078	45		
	sar7310	*sav7209*	*sav7310*	+	8718717	8718789	73		
	sar7393	*sav7392*	*sav7393*	+	8820487	8820529	43		
	sar7513	*sav7512*	*sav7513*	+	8956302	8956421	120		
	sar7536	*sav7535*	*sav7536*	+	8977882	8977950	69		
S. venezuelae									
	svr0090	*sven0089*	*sven0090*	+	86665	86870	206		
	svr0155	*sven0154*	*sven0155*	+	161764	161816	53		
	svr0164	*sven0163*	*sven0164*	+	171837	172019	183		
	svr0205	*sven0204*	*sven0205*	-	219518	219415	104		
	svr0308	*sven0307*	*sven0308*	-	327545	327508	38		
	svr0357	*sven0356*	*sven0357*	+	383067	383154	88		
	svr0359	*sven0358*	*sven0359*	+	386316	386401	86		
	svr0413	*sven0412*	*sven0413*	+	439342	439378	37		
	svr0441	*sven0440*	*sven0441*	+	473451	473516	66		
	svr0461	*sven0460*	*sven0461*	+	501812	502021	210		
	svr0475	*sven0474*	*sven0475*	+	514567	514606	40	Polyketide / non-ribosomal peptide hybrid	
	svr0552	*sven0551*	*sven0552*	-	633652	633532	121	Terpene	
	svr0651	*sven0650*	*sven0651*	-	763534	763245	290		
	svr0658	*sven0657*	*sven0658*	-	774825	774738	88		
	svr0735_1	*sven0734*	*sven0735*	+	846062	846129	68		
	svr0735_2	*sven0734*	*sven0735*	-	846191	846065	127		
	svr0785	*sven0784*	*sven0785*	-	905788	905581	208		
	svr0823	*sven0822*	*sven0823*	+	950241	950395	155		
	svr0825	*sven0824*	*sven0825*	+	952484	952535	52		
	svr0974_1*	*sven0973*	*sven0974*	-	1104401	1104137	265		
	svr0974_2	*sven0973*	*sven0974*	-	1104518	1104425	94		
	svr1004	*sven1003*	*sven1004*	-	1146315	1146248	68		
	svr1013_1	*sven1012*	*sven1013*	+	1155885	1155923	39		
	svr1013_2	*sven1012*	*sven1013*	-	1155919	1155881	39		
	svr1013_3	*sven1012*	*sven1013*	-	1155980	1155941	40		
	svr1015	*sven1014*	*sven1015*	-	1157731	1157534	198		
	svr1031	*sven1030*	*sven1031*	+	1171874	1171946	73		
	svr1032	*sven1031*	*sven1032*	+	1174057	1174096	40		

	svr1076	*sven1075*	*sven1076*	-	1219934	1219898	37		
	svr1078	*sven1077*	*sven1078*	-	1222806	1222766	41		
	svr1134	*sven1133*	*sven1134*	+	1281666	1281744	79		
	svr1153	*sven1152*	*sven1153*	+	1335028	1335071	44		
	svr1186	*sven1185*	*sven1186*	+	1335028	1335071	44		
	svr1188	*sven1187*	*sven1188*	+	1337216	1337329	114		
	svr1240	*sven1239*	*sven1240*	+	1393042	1393093	52		
	svr1249	*sven1248*	*sven1249*	-	1403844	1403774	71		
	svr1270	*sven1269*	*sven1270*	+	1431298	1431342	45		
	svr1310	*sven1309*	*sven1310*	-	1469417	1469377	41		
	svr1341	*sven1340*	*sven1341*	+	1503166	1503321	156		
	svr1427	*sven1426*	*sven1427*	-	1598148	1597742	407		
	svr1428*	*sven1427*	*sven1428*	-	1600880	1600603	278		
	svr1577	*sven1576*	*sven1577*	-	1758249	1758215	35		
	svr1663	*sven1662*	*sven1663*	-	1860448	1860336	113		
	svr1676	*sven1675*	*sven1676*	-	1871497	1871416	82		
	svr1699	*sven1698*	*sven1699*	-	1896981	1896944	38		
	svr1760	*sven1759*	*sven1760*	+	1965201	1965241	41		
	svr1766	*sven1765*	*sven1766*	+	1969717	1969824	108		
	svr1769	*sven1768*	*sven1769*	+	1972038	1972076	39		
	svr1778	*sven1777*	*sven1778*	+	1982548	1982633	86		
	svr1804	*sven1803*	*sven1804*	-	2009995	2009848	148		
	svr1820	*sven1819*	*sven1820*	-	2026024	2025909	116		
	svr1875	*sven1874*	*sven1875*	+	2086548	2086588	41		
	svr1914	*sven1913*	*sven1914*	-	2133063	2133003	61		
	svr1920	*sven1919*	*sven1920*	-	2135820	2135747	74		
	svr1942	*sven1941*	*sven1942*	+	2158399	2158459	61		
	svr2034	*sven2033*	*sven2034*	+	2246355	2246432	78		
	svr2100	*sven2099*	*sven2100*	-	2291833	2291786	48		
	svr2113	*sven2112*	*sven2113*	-	2299104	2299047	58		
	svr2128	*sven2127*	*sven2128*	+	2307576	2307608	33		
	svr2153	*sven2152*	*sven2153*	-	2323486	2323419	68		
	svr2173_1*	*sven2172*	*sven2173*	+	2338858	2338912	55		
	svr2173_2*	*sven2172*	*sven2173*	+	2339004	2339094	91		
	svr2184	*sven2183*	*sven2184*	-	2350942	2350908	35		
	svr2186	*sven2185*	*sven2186*	+	2354566	2354657	92		
	svr2213	*sven2212*	*sven2213*	-	2382190	2382148	43		
	svr2222	*sven2221*	*sven2222*	-	2391316	2391229	88		
	svr2286	*sven2285*	*sven2286*	+	2466170	2466321	152		
	svr2309	*sven2308*	*sven2309*	+	2492736	2492818	83		
	svr2325	*sven2324*	*sven2325*	-	2508047	2507989	59		
	svr2374	*sven2373*	*sven2374*	+	2564947	2564990	44		
	svr2380	*sven2379*	*sven2380*	+	2573116	2573306	191		
	svr2404	*sven2403*	*sven2404*	+	2605276	2605326	51		

	svr2416	*sven2415*	*sven2416*	+	2617740	2617829	90		
	svr2475	*sven2474*	*sven2475*	+	2692459	2692540	82		
	svr2477	*sven2476*	*sven2477*	+	2694888	2694962	75		
	svr2486	*sven2485*	*sven2486*	+	2701949	2702025	77		
	svr2501	*sven2500*	*sven2501*	-	2712672	2712634	39		
	svr2503	*sven2502*	*sven2503*	+	2713691	2713763	73		
	svr2535	*sven2534*	*sven2535*	+	2753750	2753848	99		
	svr2569	*sven2568*	*sven2569*	-	2795716	2795658	59	Siderophore	
	svr2605	*sven2604*	*sven2605*	+	2840678	2840755	78		
	svr2658	*sven2657*	*sven2658*	-	2893438	2893355	84		
	svr2670	*sven2669*	*sven2670*	+	2904517	2904558	42		
	svr2676	*sven2675*	*sven2676*	-	2909883	2909847	37		
	svr2691	*sven2690*	*sven2691*	+	2925203	2925300	98		
	svr2719	*sven2718*	*sven2719*	-	2960718	2960571	148		
	svr2763*	*sven2762*	*sven2763*	-	3026714	3026501	214		
	svr2831	*sven2830*	*sven2831*	-	3105245	3105096	150		
	svr3031	*sven3030*	*sven3031*	+	3316921	3317100	180		
	svr3058	*sven3057*	*sven3058*	-	3349419	3349351	69		
	svr3068	*sven3067*	*sven3068*	+	3362151	3362221	71		
	svr3195_1*	*sven3194*	*sven3195*	-	3501490	3501255	236		
	svr3195_2*	*sven3194*	*sven3195*	+	3501555	3501600	46		
	svr3209	*sven3208*	*sven3209*	-	3515843	3515777	67		
	svr3316	*sven3315*	*sven3316*	+	3631774	3631883	110		
	svr3328	*sven3327*	*sven3328*	-	3644356	3644215	142		
	svr3329	*sven3328*	*sven3329*	+	3645319	3645545	227		
	svr3373	*sven3372*	*sven3373*	-	3692244	3692163	82		
	svr3390	*sven3389*	*sven3390*	-	3703742	3703697	46		
	svr3412	*sven3411*	*sven3412*	+	3729165	3729223	59		
	svr3468	*sven3467*	*sven3468*	+	3780761	3781260	500		
	svr3504	*sven3503*	*sven3504*	+	3813879	3814075	197		
	svr3556	*sven3555*	*sven3556*	-	3862804	3862721	84		
	svr3584	*sven3583*	*sven3584*	-	3895923	3895890	34		
	svr3641	*sven3640*	*sven3641*	-	3954043	3953989	55		
	svr3699	*sven3698*	*sven3699*	+	4016611	4016686	76		
	svr3707	*sven3706*	*sven3707*	+	4022640	4022733	94		
	svr3746	*sven3745*	*sven3746*	-	4070199	4070157	43		
	svr3778	*sven3777*	*sven3778*	-	4105389	4105357	33		
	svr3789*	*sven3788*	*sven3789*	+	4113664	4113824	161		
	svr3832*	*sven3831*	*sven3832*	+	4157390	4157458	69		
	svr3869	*sven3868*	*sven3869*	+	4202938	4203060	123		
	svr3877*	*sven3876*	*sven3877*	+	4217760	4217964	205		
	svr3967	*sven3966*	*sven3967*	-	4308327	4307973	355		
	svr3995	*sven3994*	*sven3995*	+	4331751	4331835	85		
	svr3999	*sven3998*	*sven3999*	+	4336011	4336100	90		

	svr4012	*sven4011*	*sven4012*	+	4345906	4346105	200		
	svr4031	*sven4030*	*sven4031*	+	4365408	4365444	37		
	svr4046	*sven4045*	*sven4046*	+	4382673	4382911	239		
	svr4090	*sven4089*	*sven4090*	+	4433744	4433822	79		
	svr4155	*sven4154*	*sven4155*	+	4496853	4496938	86		
	svr4204	*sven4203*	*sven4204*	+	4548689	4548728	40		
	svr4210	*sven4209*	*sven4210*	-	4555638	4555522	117		
	svr4236	*sven4235*	*sven4236*	+	4580872	4580998	127		
	svr4279	*sven4278*	*sven4279*	+	4632238	4632272	35		
	svr4346	*sven4345*	*sven4346*	-	4703066	4702976	91		
	svr4464	*sven4463*	*sven4464*	-	4811777	4811740	38		
	svr4465	*sven4464*	*sven4465*	+	4812758	4812839	82		
	svr4477	*sven4476*	*sven4477*	-	4828765	4828614	152		
	svr4511	*sven4510*	*sven4511*	+	4865853	4865892	40		
	svr4633	*sven4632*	*sven4633*	+	4987729	4987852	124		
	svr4647	*sven4646*	*sven4647*	-	5004978	5004937	42	Melanin	
	svr4693	*sven4692*	*sven4693*	+	5048583	5048630	48		
	svr4737	*sven4736*	*sven4737*	+	5093394	5093590	197		
	svr4824	*sven4823*	*sven4824*	-	5188990	5188941	50		
	svr4904	*sven4903*	*sven4904*	-	5282887	5282654	234		
	svr5026	*sven5025*	*sven5026*	-	5410945	5410906	40		
	svr5058	*sven5057*	*sven5058*	+	5443073	5443117	45		
	svr5092	*sven5091*	*sven5092*	-	5480122	5480023	100	Butyrolactone	
	svr5117	*sven5116*	*sven5117*	-	5524420	5524299	122	Thiopeptide	
	svr5272	*sven5271*	*sven5272*	-	5695125	5694800	326		
	svr5279	*sven5278*	*sven5279*	+	5706907	5706987	81		
	svr5283	*sven5282*	*sven5283*	-	5713220	5713165	56		
	svr5316	*sven5315*	*sven5316*	+	5744324	5744401	78		
	svr5393	*sven5392*	*sven5393*	+	5837098	5837146	49		
	svr5428	*sven5427*	*sven5428*	-	5884275	5884213	63		
	svr5510	*sven5509*	*sven5510*	+	5985187	5985241	55		
	svr5514	*sven5513*	*sven5514*	+	5992941	5993104	164		
	svr5535	*sven5534*	*sven5535*	+	6017922	6017963	42		
	svr5558	*sven5557*	*sven5558*	+	6039597	6039637	41		
	svr5635	*sven5634*	*sven5635*	-	6121371	6121303	69		
	svr5651	*sven5650*	*sven5651*	-	6133699	6133654	46		
	svr5664	*sven5663*	*sven5664*	+	6147492	6147624	133		
	svr5679	*sven5678*	*sven5679*	+	6160952	6160989	38		
	svr5681	*sven5680*	*sven5681*	+	6161818	6161861	44		
	svr5700	*sven5699*	*sven5700*	+	6184424	6184564	141		
	svr5729	*sven5728*	*sven5729*	+	6213966	6214077	112		
	svr5830	*sven5829*	*sven5830*	-	6349417	6349380	38		
	svr5922	*sven5921*	*sven5922*	+	6449278	6449447	170		
	svr5940	*sven5939*	*sven5940*	-	6464189	6464112	78		

	svr5941	*sven5940*	*sven5941*	+	6468076	6468164	89		
	svr6086	*sven6085*	*sven6086*	-	6629464	6629262	203		
	svr6110	*sven6109*	*sven6110*	-	6654343	6654245	99		
	svr6112	*sven6111*	*sven6112*	+	6656954	6657029	76		
	svr6179	*sven6178*	*sven6179*	+	6730240	6730368	129	Non-ribosomal peptide	
	svr6310	*sven6309*	*sven6310*	-	6886369	6886288	82		
	svr6402	*sven6401*	*sven6402*	-	6987310	6987166	145		
	svr6475	*sven6474*	*sven6475*	-	7064808	7064763	46		
	svr6552	*sven6551*	*sven6552*	+	7160290	7160453	164		
	svr6559	*sven6558*	*sven6559*	+	7167725	7167830	106		
	svr6576	*sven6575*	*sven6576*	+	7185047	7185087	41		
	svr7049	*sven7048*	*sven7049*	-	7715443	7715260	184	Non-ribosomal peptide	
	svr7129	*sven7128*	*sven7129*	+	7825187	7825254	68		
	svr7278	*sven7277*	*sven7278*	+	8008644	8008754	111		
	svr7297	*sven7296*	*sven7297*	-	8027121	8026909	213		
	svr7351	*sven7350*	*sven7351*	+	8083855	8083893	39		

* sRNA found adjacent to a tRNA or rRNA gene

[x] unusual gene order in *S. avermitilis*

[y] locations of clusters as per Nett *et al.* (2009) for *S. avermitilis* and *S. coelicolor,* and using the program antiSMASH (Medema *et al.,* 2011) for *S. venezuelae.*

References:

Medema MH, Blin K, Cimermancic P, de Jager V, Zakrzewski P, et al. (2011) antiSMASH: rapid identification, annotation and analysis of secondary metabolite biosynthesis gene clusters in bacterial and fungal genome sequences. Nucleic Acids Res. doi: 10.1093/nar/gkr466

Nett M, Ikeda H, Moore BS (2009) Genomic basis for natural product biosynthetic diversity in the actinomycetes. Nat Prod Rep 26: 1362-1384

Table S4: Conserved intergenic sRNAs in *Streptomyces*

	S. coelicolor	*S. avermitilis*	*S. venezuelae*	Potential for encoding a short conserved ORF
**Conserved in three species**				
	scr1104	sar1503	svr0735_2	-
	scr1434	sar6912	svr1031	-
	scr1594	sar6745	svr1188	-
	scr1792	sar6487	svr1428	-
	scr2107	sar6096	svr1766	-
	scr2117	sar6085	svr1778	Y (18 amino acids)
	scr2147	sar6057	svr1804	-
	scr2634	sar5413	svr2416	-
	scr2736	sar5331_1	svr2535	-
	scr2952	sar5126	svr2719	-
	scr3077	sar3510	svr2831	-
	scr3334	sar4726	svr3195_1	-
	scr3558	sar4605	svr3328	-
	scr3559	sar4604	svr3329	-
	scr3920	sar4274	svr3699	-
	scr4076	sar4142	svr3832	-
	scr4123	sar4105_1	svr3328	-
	scr4339	sar3892	svr4155	-
	scr4395	sar3855	svr4210	-
	scr4619	sar4221	svr1924	-
	scr4677	sar3140	svr3556	-
	scr5239	sar3018	svr4904	-
	scr5583	sar2652	svr5279	-
	scr5839	sar2421	svr5514	-
	scr5917	sar0324_1	svr3468	-
	scr6214	sar2017	svr6086	-
**Conserved in *S. avermitilis* and *S. venezuelae***				
		sar4154	svr0461	-
		sar6191	svr1676	
		sar5855	svr2034	-
		sar5162	svr2670	-
		sar3677	svr3031	-
		sar4755_1	svr3556	-
		sar4227	svr3746	-
		sar3861	svr4204	-
		sar5020	svr4465	-
		sar2517	svr5393	-
		sar1689	svr6402	-
**Conserved in *S. coelicolor* and *S. avermitilis***				
	scr0959	sar7256		-
	scr1363	sar6998		Y (12-13 amino acids)

	scr1390	sar6976		-
	scr1424	sar6923		-
	scr1961	sar6284		-
	scr2101	sar6103		-
	scr2136	sar6063		-
	scr2822	sar0959		-
	scr3020	sar5057		Y (54-55 amino acids)
	scr3202	sar3694_2		-
	scr3679	sar4464		-
	scr4108	sar4119		Y (17 amino acids)
	scr4139	sar4076		-
	scr4233	sar3970		-
	scr4389	sar3858		-
	scr4689	sar0088		-
	scr5439	sar2808		-
	scr5710	sar2548		-
	scr6106	sar2124		-
**Conserved in *S. coelicolor* and *S. venezuelae***				
	scr4628		svr1914	-
	scr4684		svr3504	-

Table S5: Locations and names of 20 highly expressed stable degradation products.

Gene	CDS Start (nt position)	CDS Stop (nt position)	Strand	Stable region designation*	Stable Start (nt position)	Stable Stop (nt position)	Length
sven0433	463756	464730	+	sven0433_sr1	463897	463972	76
sven0729	840508	841308	-	sven0729_sr1	840939	841016	78
sven0847	971829	972173	+	sven0847_sr1	972049	972100	52
sven1454	1631142	1632533	+	sven1454_sr1	1631298	1631375	78
sven1480	1656022	1656387	-	sven1480_sr1	1656329	1656370	42
sven2285	2465015	2466037	-	sven2285_sr1	2465419	2465480	62
sven2374	2564998	2566152	-	sven2374_sr1	2565389	2565477	89
sven2374	2564998	2566152	-	sven2374_sr2	2565153	2565214	62
sven2917	3176014	3176742	-	sven2917_sr1	3176510	3176563	54
sven3045	3331513	3334293	-	sven3045_sr1	3332336	3332420	85
sven3682	4003974	4004264	+	sven3682_sr1	4004048	4004088	41
sven3714	4031950	4032951	+	sven3714_sr1	4032618	4032681	64
sven3715	4032972	4036562	+	sven3715_sr1	4033149	4033208	60
sven3715	4032972	4036562	+	sven3715_sr2	4033257	4033295	39
sven3715	4032972	4036562	+	sven3715_sr3	4034482	4034531	50
sven4147	4488857	4491217	-	sven4147_sr1	4490785	4490858	74
sven4589	4944297	4945223	+	sven4589_sr1	4945085	4945170	86
sven4918	5296230	5296568	+	sven4918_sr1	5296312	5296357	46
sven5046	5429952	5430920	-	sven5046_sr1	5430157	5430227	71
sven5212	5633123	5634463	+	sven5212_sr1	5633165	5633207	43

* sr denotes ‘stable region’

Table S6. Oligonucleotide primers used in this study.

Primer / probe name	Sequence (5′ - 3′)	Purpose
16S fwd	AGAGTTTGATCCTGGCTCAG	DNA contamination check; transcription profiling for 16S rRNA
16S rev	CGAACCTCGCAGATGCCTG	DNA contamination check; RT and transcription profiling for 16S rRNA
sco4606 in fwd	GTCCCCTTTCTGATCGGTCT	RT (*as_nuoL2M2*) and transcription profiling for *as_nuoL2M2* and *nuoL2/sco4606*
sco4606 in rev	ATCGTCGAGTAGGCCAGGAC	RT (*nuoL2/sco4606*) and transcription profiling for *as_nuoL2/M2* and *nuoL2/sco4606*
sco4567 in fwd	GATCGCGTACGTCAAGGAGT	RT (*as_nuoEF*) and transcription profiling for *as_nuoEF* and *nuoF/sco4567*
sco4567 in rev	GTGAGGGTGAGATCGAGTCC	RT (*nuoF/sco4567*) and transcription profiling for *as_nuoEF* and *nuoF/sco4567*
sco6762 in fwd	CTCGCCTTCTCCTTCCAGC	RT (*as_hopC*) and transcription profiling for *as_hopC* and *hopC/sco6762*
sco6762 in rev	CGATCCGGTCGAGGAACC	RT (*hopC/sco6762*) and transcription profiling for *as_hopC* and *hopC/sco6762*
sco3578 5′ in fwd	ATGATGCTCGACATGAAGCG	Transcription profiling for 5′ end of *sco3578*
sco3578 5′ in rev	GAGCGATTGGTAGAAGGGGT	RT and transcription profiling for 5′ end of *sco3578*
sco3578 3′ in fwd	CGGGAAAGCTGGACTTCGTA	RT (3′ end *wblA/sco3579*) and transcription profiling for 3′ end of *sco3578* and *wblA/sco3579*
sco3578 3′ in rev	CGTACGTGTCGGGAGAGTTG	RT (3′ end *sco3578*) and transcription profiling for 3′ end of *wblA/sco3579* and *sco3578*
sco3579 5′ in fwd	CTGCTCCCTGAACGAACAGT	RT and transcription profiling for 5′ end of *wlbA/sco3579*
sco3579 5′ in rev	GCTGGGTAACCGACTGGAG	Transcription profiling for 5′ end of *wblA/sco3579*
Conserved sRNA1	GCCCGGTGAAGGTTGAGAAGACGATCACGA	Northern probe to detect scr2634, sar5413, and svr2416
Conserved sRNA2	GGGGGAGCCGAGTCGGGCAGTTCGGGA	Northern probe to detect scr5583, sar2652, and svr5279
Conserved sRNA3	AACGAAGCCCCCGATCACGGGGGAACAA	Northern probe to detect scr1434, sar6912, and

		sve1031
sar2765	ACACGCCAGACTCTGCACTCTCCCG	Northern probe to detect sar2765.
sar3980	TCGCCTCCATTGCCGCGTTCAGACA	Northern probe to detect sar3980.
scr3716	TGCCTGGAGGCCTCGAACCGTTCCA	Northern probe to detect sar3716.
scr3931	TGGAAAGCGAACGGGTGGCCCGGTT	Northern probe to detect sar3931.
svr5535	GCCGCTCCCGGCACCGGGGCCCTCG	Northern probe to detect svr5535.
sven_2734_sr1	CCAGGGTGCCCGACACGCCGTCCGC	Northern probe to detect a stable degradation product within the coding region of *sven2734*.
5S_rRNA	CCCTGCAGTACCATCGGC	Northern probe to detect 5S rRNA.

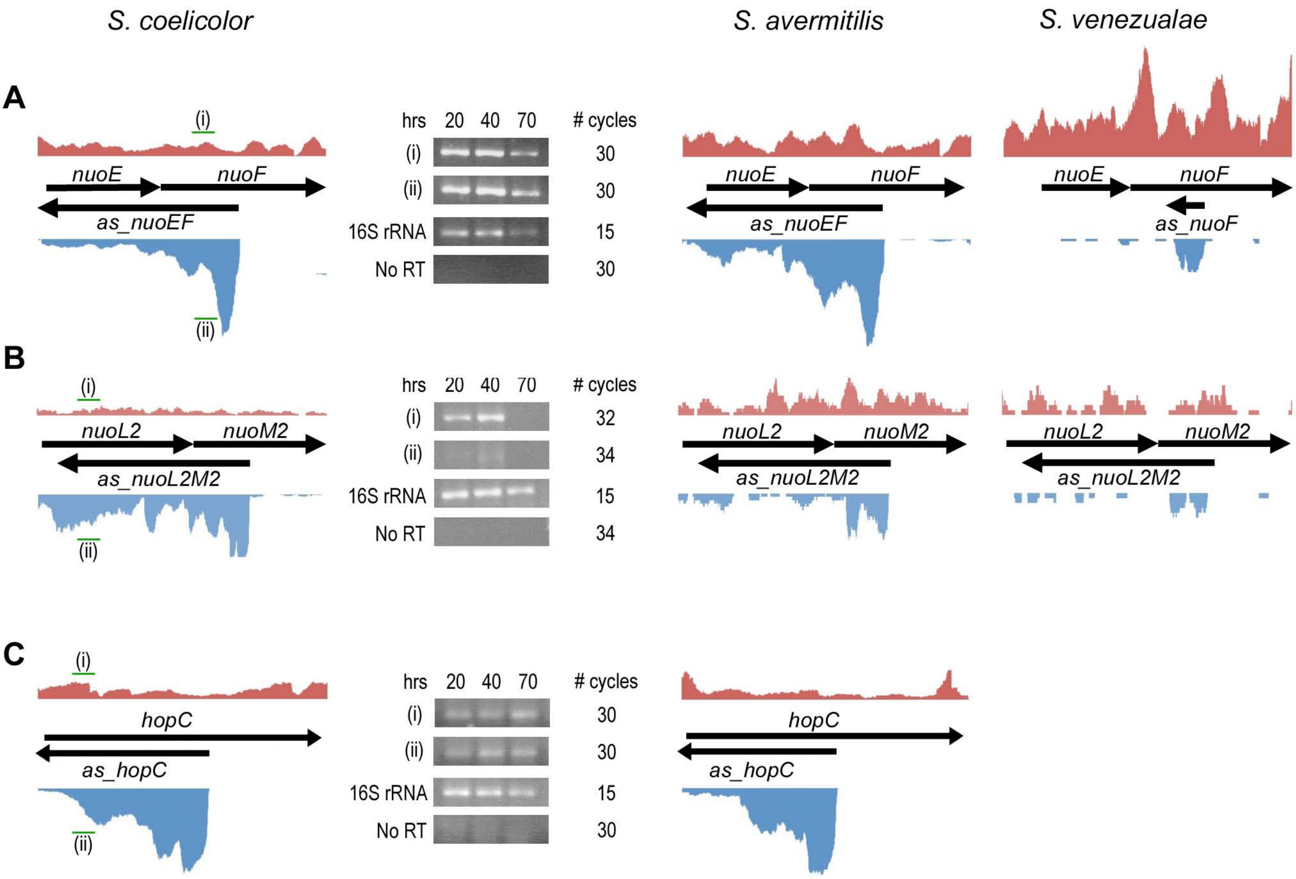

Figure S1: Expression profiles and semi-quantitative RT-PCR analysis of select antisense RNAs. A) Expression profiles (*S. coelicolor, S. avermitilis,* and *S. venezuelae*) and semi-quantitative RT-PCR analysis (*S. coelicolor*) of the antisense RNA expressed opposite *nuoE* and *nuoF*. B) Expression profiles (*S. coelicolor, S. avermitilis,* and *S. venezuelae*) and semi-quantitative RT-PCR analysis (*S. coelicolor*) of the antisense RNA expressed opposite *nuoL2* and *nuoM2*. C) Expression profiles (*S. coelicolor* and *S. avermitilis*) and semi-quantitative RT-PCR analysis (*S. coelicolor)* of the antisense RNA expressed opposite *hopC*. Relative sequence reads at each nucleotide position on the positive strand (top) are shown in red, while negative strand profiles are shown in blue. As expression levels of different genes varied over several orders of magnitude, the y-axis for each gene set was scaled independently. Semi-quantitative RT-PCR analyses examined the temporal expression of each sense and antisense gene (time of RNA extraction is indicated in hours). Reverse transcribed 16S rRNA was amplified as a control for RNA integrity and loading levels. Fragment (i) was reverse transcribed from transcripts originating from the positive strand, while (ii) was reverse transcribed from transcripts originating from the negative strand. The locations of these primers are indicated above or below the gene annotation.

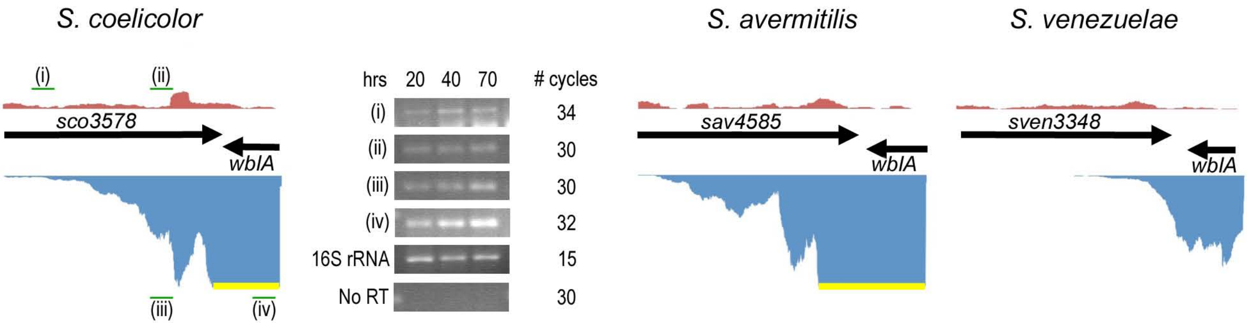

Figure S2: Expression profiles and semi-quantitative RT-PCR analysis of the *wblA* cutoRNA shared with its downstream gene in *S. coelicolor* (*sco3578*), *S. avermitilis* (*sav4585*) and *S. venezuelae* (*sven3348*). Relative sequence reads at each nucleotide position on the positive strand (top) are shown in red; negative strand profiles are shown in blue. When expression levels differed greatly for the positive and negative strand within a gene pair, the profile of the more highly expressed gene was cut off with a yellow line to ensure expression from the more lowly expressed gene could still be visualized. Semi-quantitative RT-PCR analyses over a developmental time course (time of RNA harvest is shown in hours), was conducted to assess cutoRNA expression. Reverse transcribed 16S rRNA was amplified to ensure RNA integrity and to serve as a loading control. Products (i) and (ii) were reverse transcribed from positive strand-derived transcripts, while (iii) and (iv) were reverse transcribed from transcripts originating from the negative strand. Primer locations are indicated above or below the gene annotation.
